# One-pot activation–alkynylation–cyclization synthesis of 1,5-diacyl-5-hydroxypyrazolines in a consecutive three-component fashion

**DOI:** 10.3762/bjoc.15.136

**Published:** 2019-06-19

**Authors:** Christina Görgen, Katharina Boden, Guido J Reiss, Walter Frank, Thomas J J Müller

**Affiliations:** 1 Heinrich-Heine Universität Düsseldorf, Institut für Organische Chemie und Makromolekulare Chemie, Universitätsstraße 1, D-40225 Düsseldorf, Germany; 2 Heinrich-Heine Universität Düsseldorf, Institut für Anorganische Chemie und Makromolekulare Chemie, Universitätsstraße 1, D-40225 Düsseldorf, Germany

**Keywords:** activation, alkynylation, C–C coupling, copper, cyclization, multicomponent reactions

## Abstract

A consecutive three-component activation–alkynylation–cyclization reaction of (hetero)aryl glyoxylic acids, oxalyl chloride, arylacetylenes, and hydrazides efficiently forms 1,5-diacyl-5-hydroxypyrazolines in moderate to good yields. The structures were unambiguously corroborated by comprehensive NMR spectroscopy and X-ray structure analyses of selected derivatives.

## Introduction

**Figure 1:**
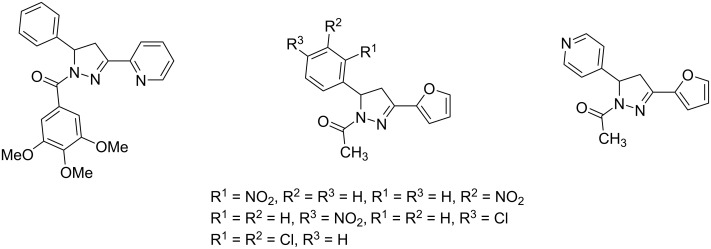
Selected anticancer active 3,5-diaryl-1-acylpyrazoline (left) and xanthine oxygenase inhibitors (center and right).

Pyrazoles [1–2] and pyrazolines [3–5] are privileged 1,2-diazole derivatives in a broad range of application, both in life and materials sciences. While the former are fully conjugated and can be considered as heteroaromatic 6π-systems with interesting properties as crop-protecting agents [6–7], as pharmaceutically active ingredients [8–11], as ligands [12–13], and as chromophores [14–16], the partially unsaturated 2*H*-pyrazolines have particularly attracted attention for instance as antibacterial [17], anti-inflammatory [18], antidiabetic [19], and antidepressive [20] agents. Especially, 1-acylpyrazolines have shown nanomolar in vitro activities against chloroquine-sensitive and resistant strains of *Plasmodium falciparum* and can therefore be considered for the treatment of malaria [21]. Furthermore, similar derivatives have shown micromolar and submicromolar activity against 60 selected cancer cell lines, presumably by inhibition of microtubuli formation in cancer cells [22]. More specifically, a series of 60 1,3-diaryl-1-acylpyrazolines was tested as xanthine oxygenase inhibitors that can be efficacious against articular gout, cancer, and inflammation, with IC_50_ values of four derivatives in the range of 5.3–15.2 μM (Figure 1) [23].

**Figure 2:**
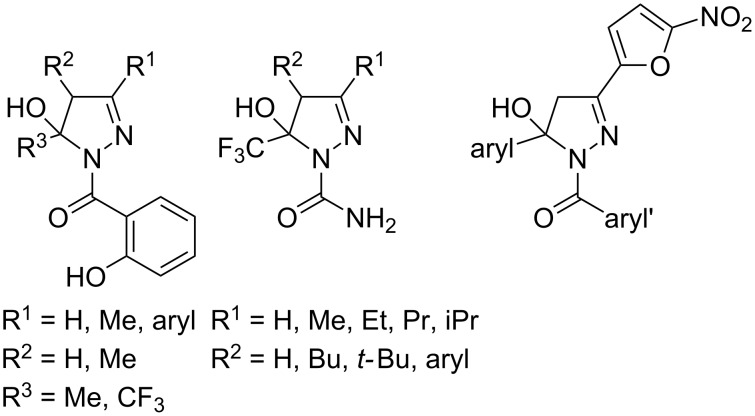
Selected 1-acyl-5-hydroxypyrazolines with analgesic (left, center) and antibacterial activity (center and right).

1-Acyl-5-hydroxypyrazolines have been shown to be analgesics with a slightly improved pain-relieving efficacy than Aspirin^®^ [24–25], and 5-nitro-2-furyl-substituted derivatives are active antibacterials against the strains *S. aureus*, *A. aerogenes*, *E. coli* and *B. subtilis* (Figure 2) [26–27].

In addition, 1-acyl-5-hydroxypyrazolines are bidentate ligands for zinc complexes and by virtue of being ring tautomers of β-enolhydrazones they can also act as tridentate ligands for nickel [28] and tin [29–30] complexes. In contrast, dimethylzinc forms dimeric complexes where the 1-acyl-5-hydroxypyrazoline acts as a bidentate ligand [31]. Upon treatment with TMEDA mononuclear complexes with concomitant ring opening to give a seven-membered bidentate chelate are generated.

**Scheme 1:**
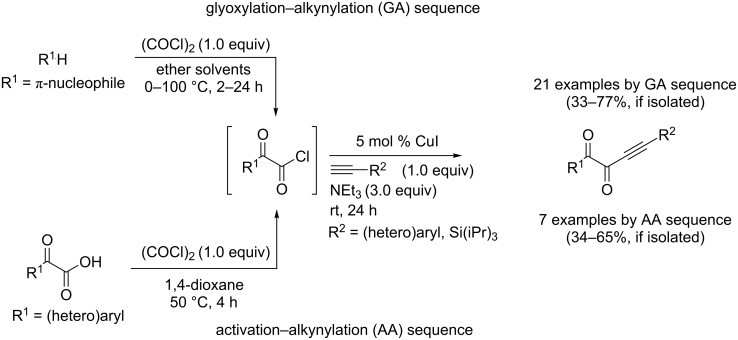
Glyoxylation–alkynylation (GA) and activation–alkynylation (AA) synthesis of alkynediones in a one-pot fashion.

Although numerous syntheses of pyrazolines [3–5] in general and 1-acyl-5-hydroxypyrazolines [24–26] specifically have been published employing a cyclizing addition of an acylhydrazone to the carbonyl group as a ring-forming reaction [32–40], their diversity-oriented one-pot synthesis in a multicomponent approach has remained unexplored to date. In the course of our program directed to develop multicomponent syntheses of heterocycles by transition-metal catalysis [41–42] we conceptualized catalytic entries to alkynones and alkynediones as suitable intermediates in addition–cyclocondensation syntheses of numerous heterocycles, which can indeed be prepared by consecutive multicomponent reactions [43–47]. Particularly interesting are alkynediones, because, as densely functionalized trielectrophiles, the alkyne, ynone and dicarbonyl functionalities can be selectively addressed. We have established two complementary one-pot pathways to alkynediones, a glyoxylation–alkynylation (GA) [48] and an activation–alkynylation (AA) [49] sequence, which both take advantage of a copper-catalyzed alkynylation of the intermediary formed (hetero)arylglyoxyl chloride (Scheme 1). The alkynediones can be subsequently transformed, still in the same reaction vessel, to quinoxalines [48,50–52], pyrimidines [48–49], and 5-acylpyrazoles [48–49]. The latter 5-acylpyrazole arose after work-up from the three-component AA–cyclocondensation synthesis employing Boc-hydrazine as a binucleophilic hydrazide substrate. Based on our attempts to isolate potential 1,5-diacylpyrazole precursors we discovered that 1,5-diacyl-5-hydroxypyrazolines are the intermediary products. Here, we report on the novel three-component AA–condensation–cyclization synthesis of 1,5-diacyl-5-hydroxypyrazolines.

## Results and Discussion

**Scheme 2:**
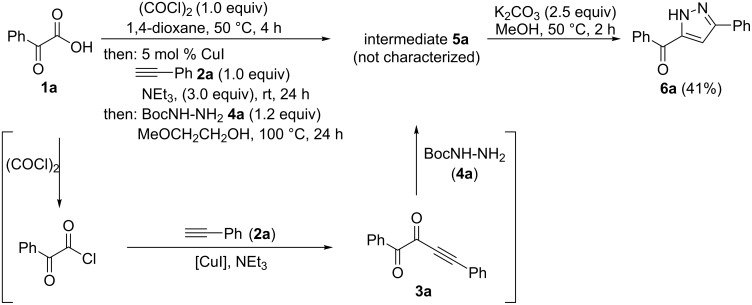
Consecutive three-component synthesis to give 5-benzoyl-3-phenyl-1*H*-pyrazole (**6a**) after alkaline deacylation of intermediate **5a**.

In our initial study [49], the three-component AA–cyclocondensation synthesis, starting from phenylglyoxylic acid (**1a**), phenylacetylene (**2a**), and Boc-hydrazide (**4a**) through the formation of 1,4-diphenylbut-3-yne-1,2-dione (**3a**), with subsequent *N*-deacylation as the consequence of basic work-up (Scheme 2), furnished 5-benzoyl-3-phenyl-1*H*-pyrazole (**6a**) in 41% isolated yield.

**Figure 3:**
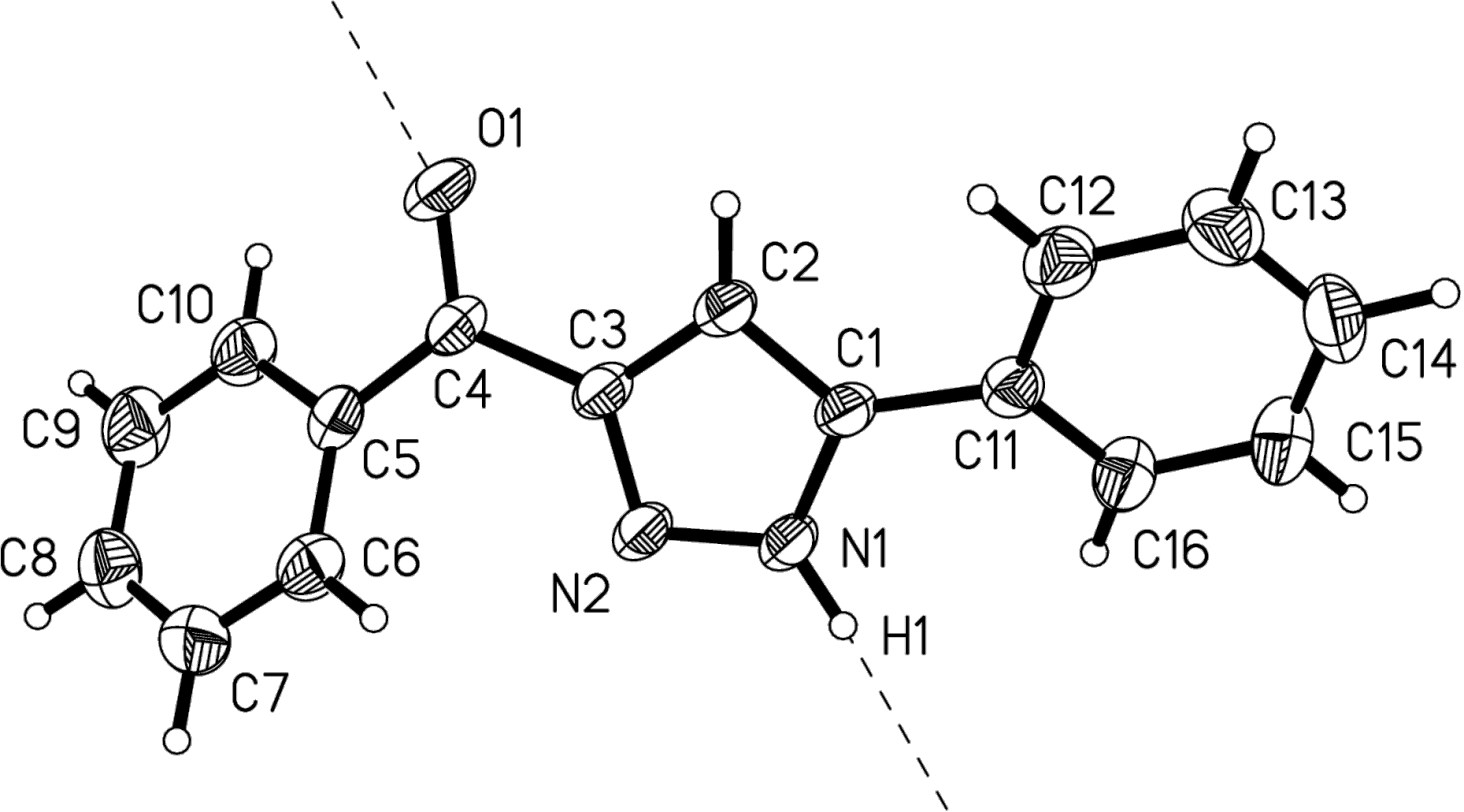
ORTEP plot of 5-benzoyl-3-phenyl-1*H*-pyrazole (**6a**) (thermal ellipsoids at 30% probability); the direction of intermolecular N−H···O hydrogen bonding is indicated by dashed lines.

In addition to spectroscopic assignment the structure of **6a** has now been corroborated by an X-ray structure analysis showing infinite chains of molecules **6a** formed by intermolecular hydrogen bonding between the pyrazole N1 and the carbonyl O1 (Figure 3) [53].

**Scheme 3:**
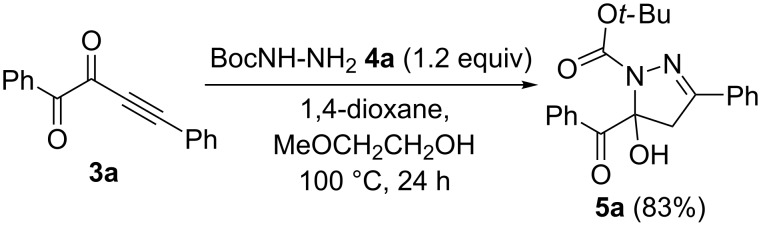
Cyclization of 1,4-diphenylbut-3-yne-1,2-dione (**3a**) and Boc-hydrazine (**4a**) to give intermediate **5a**.

The first assumption was that the tentative intermediate **5a** could be a 1,5-diacylpyrazole. However, upon performing the terminal cyclization step starting from 1,4-diphenylbut-3-yne-1,2-dione (**3a**) and Boc-hydrazine (**4a**) under identical conditions 1-Boc-5-benzoyl-5-hydroxypyrazoline was isolated in 83% yield (Scheme 3).

**Figure 4:**
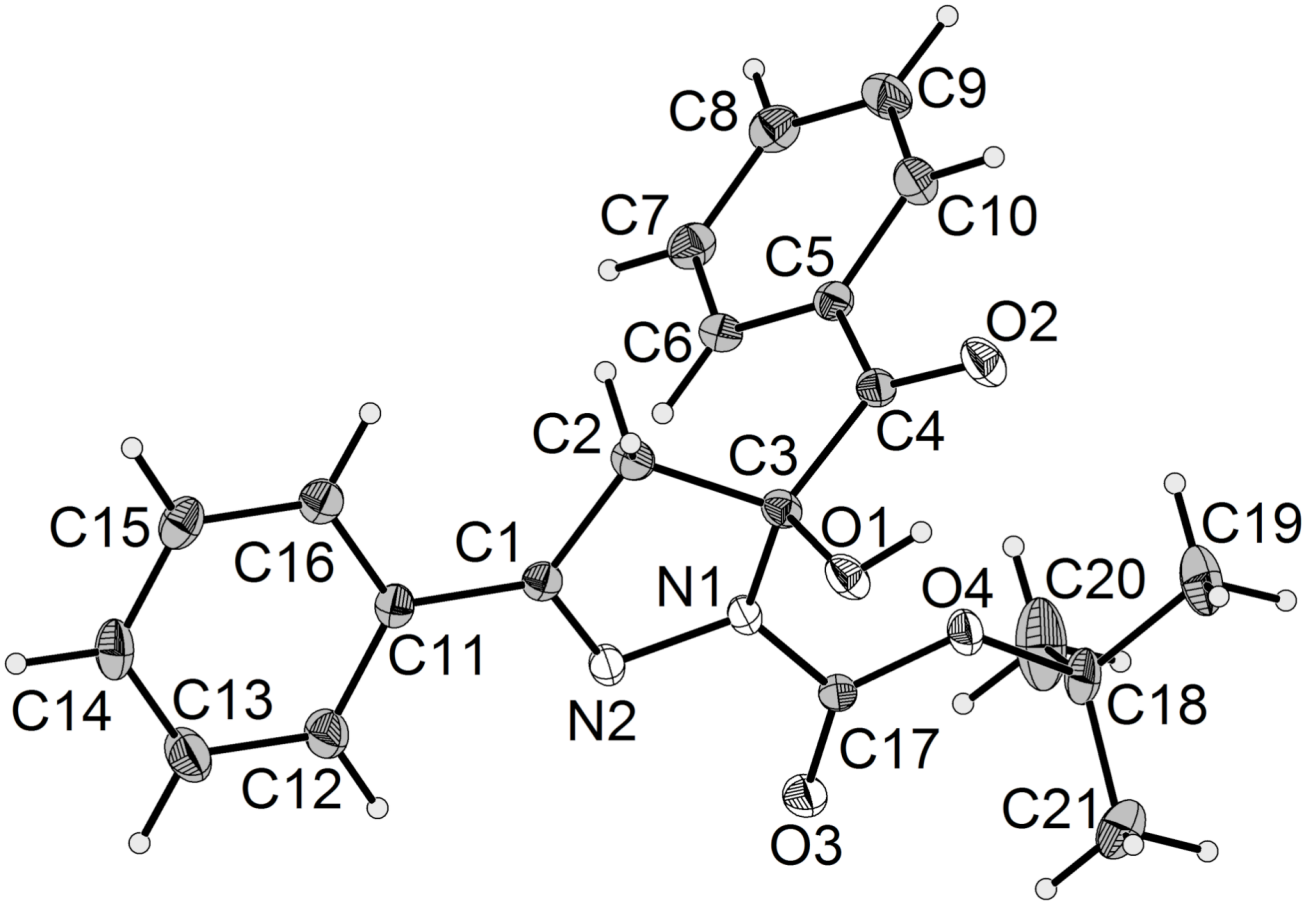
Ellipsoid plot of 1-Boc-5-benzoyl-5-hydroxypyrazoline **5a**.

The molecular structure was additionally corroborated by X-ray structure analysis showing that the assignment of intermediate **5a** was not a fully unsaturated pyrazole (Figure 4) [53].

**Scheme 4:**
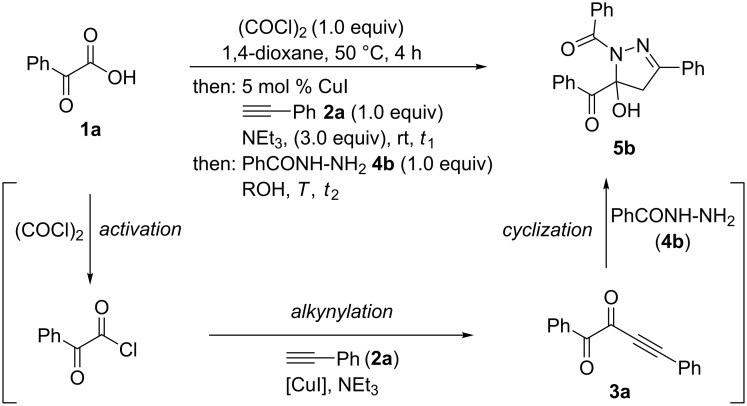
Model reaction for optimizing the activation–alkynylation–cyclization synthesis of 1,5-diacyl-5-hydroxypyrazoline **5b**.

Therefore, we set out to optimize the one-pot synthesis of 1,5-diacyl-5-hydroxypyrazolines by choosing the model reaction of phenylglyoxylic acid (**1a**), phenylacetylene (**2a**), and benzoyl hydrazide (**4b**) giving 1,5-diacyl-5-hydroxypyrazoline **5b**, where the reaction times *t*_1_ and *t*_2_, as well as the conditions of the cyclization step needed to be optimized (Scheme 4).

A quick optimization screening of the activation–alkynylation synthesis of 1,4-diphenylbut-3-yne-1,2-dione (**3a**) revealed that the use of KOH dried triethylamine instead of the initial preconditioning (Na/benzophenone dried) led to a reduction of the reaction time *t*_1_ from 24 to 15 h (see Supporting Information File 1, Table S1). In addition, the concentration could be doubled and the obtained yield of diphenylbut-3-yne-1,2-dione (**3a**) increased from 63 to 76%.

The terminal cyclization step, consisting of a Michael addition of benzoyl hydrazide (**4b**) to diphenylbut-3-yne-1,2-dione (**3a**) followed by a cyclizing addition of the central hydrazide nitrogen atom to the carbonyl group, was monitored by GC–MS and optimized with respect to temperature *T*, reaction time *t*_2_, and the alcohol additive (Table 1).

**Table 1:** Optimization of the cyclization step of 1,5-diacyl-5-hydroxypyrazoline **5b**.^a^

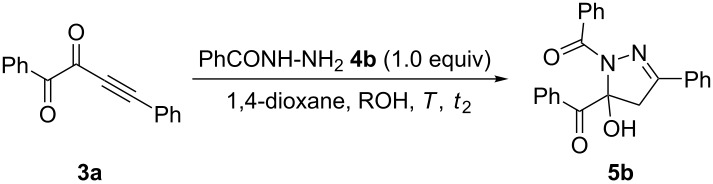				

entry	ROH [mL]	*T* [°C]	*t*_2_ [min]	1,5-diacyl-5-hydroxypyrazoline **5b** (%)^b^

1^c,d^	2-methoxyethanol (0.2)	100	60	incomplete conversion^e^ (n.i.)
2^c,d^	2-methoxyethanol (0.2)	150	60	incomplete conversion^e^ (n.i.)
3^d,f^	2-methoxyethanol (0.2)	150	60	complete conversion^e^ (n.i.)
4^d,g^	2-methoxyethanol (0.2)	150	60	complete conversion^e^ (n.i.)
5^d,h^	2-methoxyethanol (0.2)	150	60	complete conversion^e^ (n.i.)
6^d,i^	2-methoxyethanol (0.2)	150	60	incomplete conversion^e^ (n.i.)
7^d,h^	2-methoxyethanol (0.2)	150	30	complete conversion^e^ (n.i.)
8^d,h^	2-methoxyethanol (0.2)	150	15	complete conversion^e^ (n.i.)
9^d,h^	2-methoxyethanol (0.2)	150	5	incomplete conversion^e^ (n.i.)
10^d,h^	2-methoxyethanol (0.2)	100	10	incomplete conversion^e^ (n.i.)
11^d,h^	2-methoxyethanol (0.2)	125	10	incomplete conversion^e^ (n.i.)
**12**^d,h,j^	**2-methoxyethanol (0.2)**	**175**	**5**	**full conversion**^e^** (94)**
13^d,h,j^	ethylene glycol (0.2)	175	5	full conversion^e^ (96)
14^d,h,j^	ethanol (0.2)	175	5	full conversion^e^ (87)
**15**^k^	**2-methoxyethanol (0.2)**	**175**	**5**	**full conversion**^e^** (90)**
16^h,l,j^	2-methoxyethanol (0.2)	175	5	full conversion^e^ (93)

^a^*c*_0_(**3a**) = 0.17 M; 1,4-dioxane (1.0 mL). ^b^Isolated yield (n.i. = not isolated). ^c^*c*_0_(**4b**) = 0.17 M. ^d^Dielectric heating in a microwave cavity (*T* is the set temperature and *t*_2_ is the hold time). ^e^As monitored by GC–MS. ^f^*c*_0_(**4b**) = 0.25 M. ^g^*c*_0_(**4b**) = 0.21 M. ^h^*c*_0_(**4b**) = 0.20 M. ^i^*c*_0_(**4b**) = 0.18 M. ^j^On a 1.00 mmol scale (**3a**). ^k^On a 1.00 mmol scale (**3a**), *c*_0_(**3a**) = 0.34 M; *c*_0_(**4b**) = 0.40 M. 1,4-Dioxane (1.0 mL). ^l^Conductive heating in an oil bath at preheated temperature *T*.

A ratio of 1.2 equiv of hydrazide **4b** to 1.0 equiv of **3a** turned to be optimal for achieving full conversion (Table 1, entries 7–16) and at a reaction temperature of 175 °C the reaction time of 5 min was identified to achieve full conversion with very good to excellent yields of isolated 1,5-diacyl-5-hydroxypyrazoline **5b** (Table 1, entries 12–16). Although ethylene glycol as a cosolvent (Table 1, entry 13) gave slightly higher yields and ethanol furnished slightly lower yields (Table 1, entry 14), 2-methoxyethanol not only gave high yields of **5b**, but also proved to be practical with respect to work-up. Upon comparison between dielectric and conductive heating the reaction in the microwave cavity gave no detectable difference in reaction time and yield. All these optimized conditions were therefore directly employed in the consecutive one-pot sequence. However, some adjustments in the final step were necessary because an increase of pressure was detected under dielectric heating. Therefore, the consecutive process was optimized with respect to the terminal step (Table 2).

**Table 2:** Optimization of the consecutive three-component synthesis of 1,5-diacyl-5-hydroxypyrazoline **5b**.

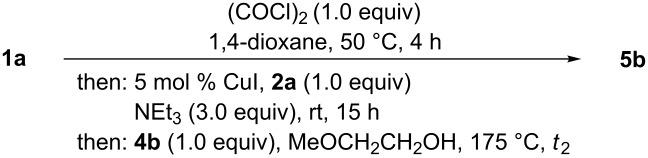			

entry	*c*_0_(**1a**)	*t*_2_ [min]	1,5-diacyl-5-hydroxypyrazoline **5b**, yield [%]^a^

1^b^	0.4 M	5	37
2^b^	0.25 M	5	32
3^b^	0.25 M	10	35
4^c^	0.4 M	10	no product formation^d^
5^e,f^	0.4 M	5	no product formation^d^
6^e^	0.4 M	10	64
7^e^	0.4 M	20	69
**8**^e^	**0.4 M**	**30**	**78**
9^e^	0.4 M	45	79

^a^Isolated yield. ^b^Dielectric heating in a microwave cavity (*T* is set to 175 °C and *t*_2_ is the hold time). ^c^Dielectric heating in a microwave cavity (*T* is set to 150 °C and *t*_2_ is the hold time). ^d^As monitored by GC–MS. ^e^Conductive heating in an oil bath at preheated temperature *T* = 175 °C. ^f^2.00 equiv of NEt_3_ were added.

In the sequence dielectric heating gave considerably lower yields (Table 2, entries 1–3) than in the separated process (Table 1, entries 12–15). However, conductive heating, which already gave comparable results in the terminal cyclization step (Table 1, entry 16), is obviously better suited to achieve full conversion and, ultimately, slightly longer heating also gives rise to good yields (Table 2, entries 6–9).

Taking into account the combined yield of 71% for both individually performed steps (ynedione formation with 76% and cyclization with 94%) is slightly lower than that of the one-pot sequence with 78% (Table 2, entry 8), the consecutive three-component process clearly is superior. With four bond-forming steps (activation, alkynylation, Michael addition, and cyclization) the average yield per bond-forming step accounts to 94%.

**Scheme 5:**
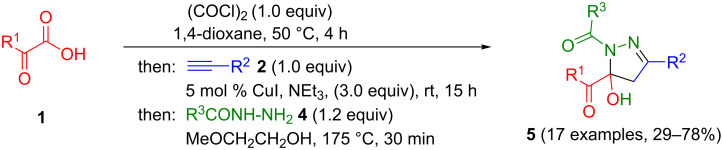
One-pot activation–alkynylation–cyclization synthesis of 1,5-diacyl-5-hydroxypyrazolines **5**.

With the optimized conditions of the consecutive three-component synthesis in hand (hetero)arylglyoxylic acids **1**, oxalyl chloride, arylacetylenes **2**, and hydrazides **4** were reacted in 1,4-dioxane and in the presence of catalytic amounts of copper(I) iodide in a one-pot activation–alkynylation–cyclization sequence to give 1,5-diacyl-5-hydroxypyrazoline **5** after flash chromatography on silica gel in moderate to good yields (Scheme 5, Table 3).

**Table 3:** Consecutive three-component synthesis of 1,5-diacyl-5-hydroxypyrazolines **5**.

entry	glyoxylic acid R^1^COCO_2_H **1**	alkyne R^2^C≡CH **2**	hydrazide R^3^CONHNH_2_ **4**	1,5-diacyl-5-hydroxypyrazoline **5** yield

1	R^1^ = Ph (**1a**)	R^2^ = Ph (**2a**)	R^3^ = Ph (**4b**)	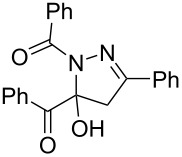 **5b** (78%)
2^a^	**1a**	**2a**	R^3^ = *p*-MeC_6_H_4_ (**4c**)	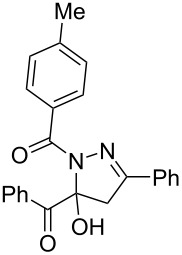 **5c** (55%)
3	**1a**	**2a**	R^3^ = *p*-BrC_6_H_4_ (**4d**)	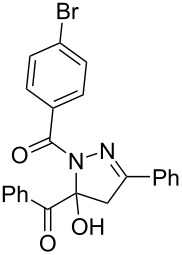 **5d** (41%)
4	**1a**	**2a**	R^3^ = 2-thienyl (**4e**)	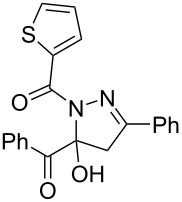 **5e** (67%)
5	**1a**	**2a**	R^3^ = 2-furyl (**4f**)	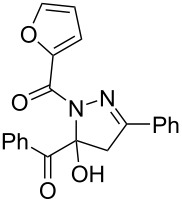 **5f** (67%)
6	**1a**	**2a**	R^3^ = PhCH_2_ (**4g**)	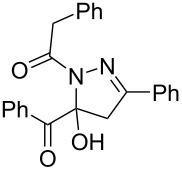 **5g** (59%)
7	**1a**	**2a**	R^3^ = iPr (**4h**)	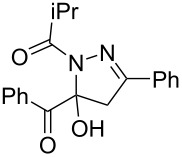 **5h** (66%)
8	**1a**	**2a**	R^3^ = cyclopropyl (**4i**)	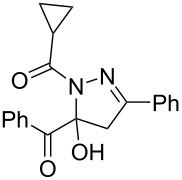 **5i** (69%)
9	**1a**	**2a**	R^3^ = *t-*Bu (**4j**)	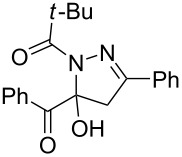 **5j** (58%)
10	**1a**	**2a**	R^3^ = *n-*Pr (**4k**)	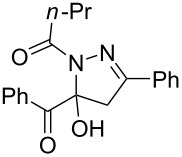 **5k** (33%)
11	**1a**	R^2^ = *p*-MeOC_6_H_4_ (**2b**)	**4b**	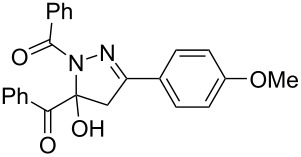 **5l** (55%)
12	**1a**	R^2^ = *p*-*t-*BuC_6_H_4_ (**2c**)	**4b**	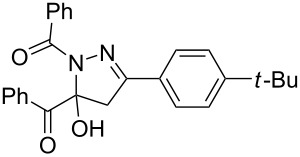 **5m** (69%)
13	**1a**	R^2^ = *p*-FC_6_H_4_ (**2d**)	**4b**	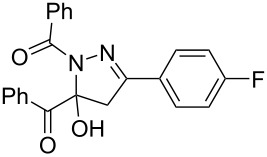 **5n** (66%)
14	**1a**	R^2^ = *p*-NCC_6_H_4_ (**2e**)	**4b**	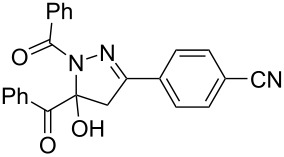 **5o** (29%)
15	R^1^ = 2,4,6-Me_3_C_6_H_2_ (**1b**)	**2a**	**4b**	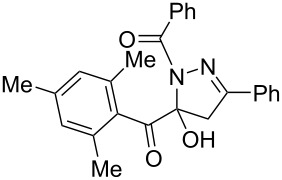 **5p** (47%)
16	R^1^ = 2-thienyl (**1c**)	**2a**	**4b**	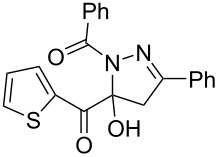 **5q** (73%)
17	**1b**	**2b**	**4e**	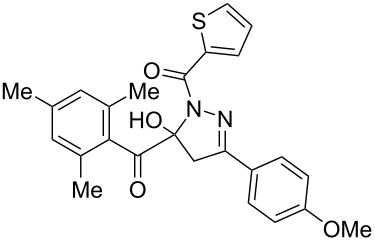 **5r** (38%)

^a^Reaction time *t*_2_ = 20 min.

**Figure 5:**
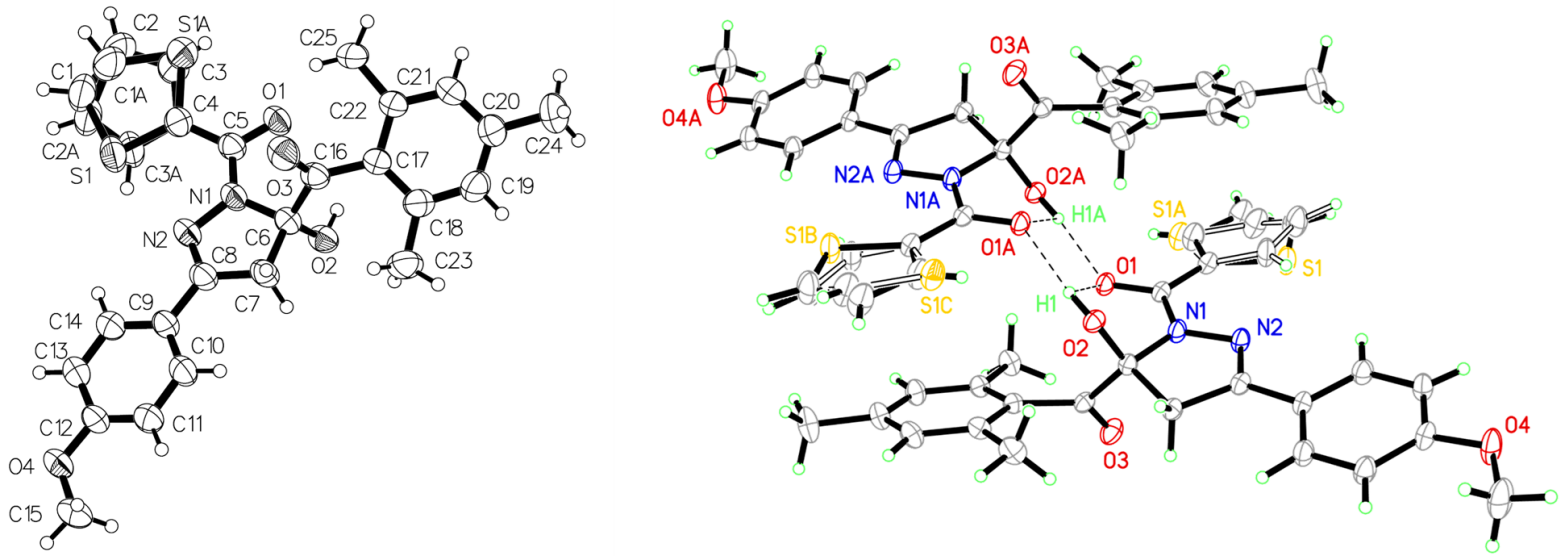
ORTEP plot and dimer of compound **5r** (thermal ellipsoids at 30% probability).

The structures of the 1,5-diacyl-5-hydroxypyrazolines **5** were unambiguously assigned by ^1^H and ^13^C NMR spectroscopy, in selected cases by NOESY, HSQC, and HMBC experiments, as well as by EI mass spectrometry and the elemental composition was confirmed by combustion analyses. Additionally, the structure was corroborated by an X-ray structure analysis of compound **5r** showing dimers held together by inter- and intramolecular hydrogen bonding (Figure 5) [53].

The three-component synthesis allows addressing three points of diversity and especially for the hydrazide substrate **4** all different types of (hetero)aromatic, aliphatic, and alicyclic substituents R^3^ are well tolerated in the sequence (Table 3, entries 1–10). The alkynes **2** can bear electron-donating and electron-withdrawing substituents R^2^ (Table 3, entries 1, 11–14), however, for the electron-poor cyano substituent a somewhat lower yield of the title compound is obtained (Table 3, entry 14). Finally, the substituents R^1^ of the glyoxylic acids **1** can be aromatic, heteroaromatic and even sterically demanding (Table 3, entries 1, 15–17).

All attempts to dehydrate 1,5-diacyl-5-hydroxypyrazoline **5b** under alkaline or Brønsted acidic conditions were accompanied by simultaneous deacylation of substituent R^3^ finally furnishing 5-(hetero)aroyl-3-(hetero)aryl-1*H*-pyrazole **6a** (for attempted dehydrative aromatization, see Supporting Information File 1, Table S5), as already reported for alkaline deprotection–aromatization [49]. However, compound **5b** is stable against water and weakly basic conditions. This indicates that 1,5-diacyl-5-hydroxypyrazolines might act as acyl transferring agents under certain conditions.

## Conclusion

In summary we could elucidate that the consecutive three-component activation–alkynylation–cyclization sequence of (hetero)arylglyoxylic acids, oxalyl chloride, arylacetylenes, and hydrazides does not form aromatic pyrazoles, but rather 1,5-diacyl-5-hydroxypyrazolines, i.e., the aromatizing elimination of water does not occur under these neutral conditions. This novel one-pot synthesis of 1,5-diacyl-5-hydroxypyrazolines is concise, highly efficient and diversity-oriented. The deacylating aromatization of the title compounds under weakly alkaline or acidic conditions indicates acyl-transfer ability. Furthermore, the peculiar reactivity of the ynedione intermediate calls for more sophisticated cyclizing processes, eventually in a one-pot fashion. Further studies exploring the dense electrophilic reactivity of ynediones in consecutive multicomponent reactions are still underway.

## Experimental

**Typical procedure for the three-component synthesis of compound 5b:** In an oven-dried Schlenk flask equipped with a magnetic stirring bar and screw cap were placed glyoxylic acid **1a** (150 mg, 1.00 mmol) and dry 1,4-dioxane (2.5 mL) under argon. Then, oxalyl chloride (0.09 mL, 1.00 mmol) was added dropwise at room temperature (external water bath) and the reaction mixture was stirred at 50 °C (preheated oil bath) for 4 h. After the mixture had cooled to room temperature, CuI (10 mg, 0.05 mmol), phenylacetylene (**2a**, 0.11 mL, 1.00 mmol), and dry triethylamine (0.42 mL, 3.00 mmol) were successively added. Stirring at room temperature (external water bath) was continued for 15 h. Then, phenylhydrazide (**3b**, 163 mg, 1.20 mmol) and 2-methoxyethanol (1.0 mL) were added and the reaction mixture was stirred at 175 °C (preheated oil bath) for 30 min. After cooling to room temperature deionized water (5 mL) was added and the mixture was extracted with dichloromethane (4 × 5 mL). The combined organic phases were dried with anhydrous sodium sulfate and the solvents were removed in vacuo. The crude product was adsorbed on celite^©^ and purified by flash chromatography on silica gel (petroleum ether 40–60 °C/ethyl acetate 5:1) to give analytically pure 1,5-dibenzoyl-5-hydroxy-3-phenylpyrazoline (**5b**, 291 mg, 78%) as colorless solid. *R_f_* = 0.15 (petroleum ether/ethyl acetate 5:1, detected with a hand-held UV lamp at 254 and 365 nm). Mp 152 °C; ^1^H NMR (CDCl_3_, 300 MHz) δ 3.54 (d, *J* = 18.5 Hz, 1H), 3.76 (d, *J* = 18.5 Hz, 1H), 5.60–6.08 (br, 1H), 7.36–7.62 (m, 9H), 7.72–7.83 (m, 2H), 7.90–8.05 (m, 4H); ^13^C NMR (CDCl_3_, 75 MHz) δ 45.6 (CH_2_), 92.2 (C_quat_), 126.9 (CH), 127.8 (CH), 128.9 (CH)*, 129.0 (CH), 130.2 (CH), 130.7 (C_quat_), 130.9 (CH), 131.7 (CH), 131.8 (C_quat_), 132.9 (C_quat_), 133.9 (CH), 153.1 (C_quat_), 166.7 (C_quat_), 193.4 (C_quat_); *broadened signal; EIMS (*m*/*z*): 352 ([M − H_2_O])^+^, 2), 266 (11), 265 ([M − PhCO]^+^, 59), 248 ([M − PhCO − H_2_O]^+^, 20), 105 (PhCO^+^, 100), 77 (C_6_H_5_^+^, 34); IR (ATR), 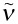 [cm^−1^]: 3333 (w), 1697 (m), 1626 (m), 1612 (m), 1566 (w), 1450 (m), 1427 (m), 1339 (m), 1315 (w), 1254 (w), 1202 (m), 1180 (m), 1113 (m), 1057 (w), 1028 (w), 922 (w), 895 (w), 866 (m), 845 (w), 791 (w), 762 (m), 708 (s), 689 (s), 669 (m), 627 (w); anal. calcd for C_23_H_18_N_2_O_3_ (370.4): C, 74.58; H, 4.90; N, 7.56; found: C, 74.67; H, 5.07; N, 7.79.

## Supporting Information

For experimental details of the optimization studies on intermediate **3a**, on the cyclization step of **3a** and **4b** (compound **5b**), on the consecutive three-component synthesis of compound **5b**, experimental details of general procedure of the consecutive three-component synthesis and analytical data of 1,5-diacyl-5-hydroxypyrazolines **5**, experimental details on the attempted dehydrative aromatization of compound **5b**, and NMR spectra of the compounds **5**, and for summaries on the crystal structure analyses of **5a**, **5r**, and **6a** see Supporting Information File 1.

File 1 Experimental details, copies of NMR spectra and crystallographic data.

## Statement

The reported results have been summarized in the inaugural dissertation "Diversitätsorientierte katalytische Ein-Topf-Synthesen von ausgewählten Azolderivaten" by Dr. Christina Boersch, Heinrich Heine University Düsseldorf, 2014. Dr. Christina Görgen (née Boersch) is the first author of this article.
