# Supplementary material for: One-pot activation–alkynylation–cyclization synthesis of 1,5-diacyl-5-hydroxypyrazolines in a consecutive three-component fashion
Source: Beilstein J Org Chem. 2019 Jun 19;15:1360–70. doi: 10.3762/bjoc.15.136 (PMC6604727; doi:10.3762/bjoc.15.136)
Supplement: File 1 — Experimental details, copies of NMR spectra and crystallographic data. [file Beilstein_J_Org_Chem-15-1360-s001.pdf]

BEILSTEIN JOURNAL OF ORGANIC CHEMISTRY

Supporting Information

for

One-pot activation–alkynylation–cyclization synthesis of 1,5-diacyl-5-hydroxypyrazolines in a consecutive three-component fashion

Christina Görgen, Katharina Boden, Guido J. Reiss, Walter Frank and Thomas J. J. Müller

Beilstein J. Org. Chem. **2019,** *15,* 1360–1370. doi:10.3762/bjoc.15.136

Experimental details, copies of NMR spectra and crystallographic data

License and Terms: This is a supporting information file under the terms of the Creative Commons Attribution License (http://creativecommons.org/licenses/by/4.0). Please note that the reuse, redistribution and reproduction in particular requires that the authors and source are credited.
The license is subject to the *Beilstein Journal of Organic Chemistry* terms and conditions: (https://www.beilstein-journals.org/bjoc)

1 **General considerations** S3
2 **Optimization studies** S4
2.1 Optimization of 1,4-diphenylbut-3-yn-1,2-dione (**3a**) S4
2.2 Synthesis of *tert*-butyl 5-benzoyl-5-hydroxy-3-phenyl-4,5-dihydro-1*H*-pyrazole-1-carboxylate (**5a**) S5
2.3 Optimization of *tert*-butyl 5-benzoyl-5-hydroxy-3-phenyl-4,5-dihydro-1*H*-pyrazole-1-carboxylate (**5b**) S6
2.4 Optimization of consecutive three-component synthesis of *tert*-butyl 5-benzoyl-5-hydroxy-3-phenyl-4,5-dihydro-1*H*-pyrazole-1-carboxylate (**5b**) S8
3 **Three-component synthesis of 1,5-diacyl-5-hydroxypyrazolines 5** S9
3.1 General procedure (GP) S9
3.2 (5-Hydroxy-3-phenyl-4,5-dihydro-1*H*-pyrazol-1,5-diyl)bis(phenylmethanone) (**5b**) S10
3.3 (5-Benzoyl-5-hydroxy-3-phenyl-4,5-dihydro-1*H*-pyrazol-1-yl)(4-tolyl)methanone (**5c**) S11
3.4 (5-Benzoyl-5-hydroxy-3-phenyl-4,5-dihydro-1*H*-pyrazol-1-yl)(4-bromophenyl)methanone (**5d**) S11
3.5 (5-Benzoyl-5-hydroxy-3-phenyl-4,5-dihydro-1*H*-pyrazol-1-yl)(thien-2-yl)methanone (**5e**) S12
3.6 (5-Benzoyl-5-hydroxy-3-phenyl-4,5-dihydro-1*H*-pyrazol-1-yl)(furan-2-yl)methanone (**5f**) S12
3.7 1-(5-Benzoyl-5-hydroxy-3-phenyl-4,5-dihydro-1*H*-pyrazol-1-yl)-2-phenylethan-1-one (**5g**) S13
3.8 1-(5-Benzoyl-5-hydroxy-3-phenyl-4,5-dihydro-1*H*-pyrazol-1-yl)-2-methylpropan-1-one (**5h**) S13
3.9 (5-Benzoyl-5-hydroxy-3-phenyl-4,5-dihydro-1*H*-pyrazol-1-yl)(cyclopropyl)-methanone (**5i**) S14
3.10 1-(5-Benzoyl-5-hydroxy-3-phenyl-4,5-dihydro-1*H*-pyrazol-1-yl)-2,2-dimethylpropan-1-one (**5j**) S14
3.11 1-(5-Benzoyl-5-hydroxy-3-phenyl-4,5-dihydro-1*H*-pyrazol-1-yl)butan-1-one (**5k**) S15
3.12 (5-Hydroxy-3-(4-methoxyphenyl)-4,5-dihydro-1*H*-pyrazol-1,5-diyl)bis(phenylmethanone) (**5l**) S15
3.13 (3-(4-(*tert*-Butyl)phenyl)-5-hydroxy-4,5-dihydro-1*H*-pyrazol-1,5-diyl)bis(phenylmethanone) (**5m**) S16
3.14 (3-(4-Fluorophenyl)-5-hydroxy-4,5-dihydro-1*H*-pyrazol-1,5-diyl)bis(phenylmethanone) (**5n**) S16
3.15 4-(1,5-Dibenzoyl-5-hydroxy-4,5-dihydro-1*H*-pyrazol-3-yl)benzonitrile (**5o**) S17
3.16 (1-Benzoyl-5-hydroxy-3-phenyl-4,5-dihydro-1*H*-pyrazol-5-yl)(mesityl)methanone (**5p**) S17
3.17 (1-Benzoyl-5-hydroxy-3-phenyl-4,5-dihydro-1*H*-pyrazol-5-yl)(thien-2-yl)methanone (**5q**) S18
3.18 (5-Hydroxy-3-(4-methoxyphenyl)-1-(thiophen-2-carbonyl)-4,5-dihydro-1*H*-pyrazol-5-yl)(mesityl)methanone (**5r**) S18
4 **Attempted aromatization of 1,5-diacyl-5-hydroxypyrazoline 5b** S19
5 **^{1}H and ^{13}C NMR spectra** S20
5.1 *tert*-Butyl 5-benzoyl-5-hydroxy-3-phenyl-4,5-dihydro-1*H*-pyrazole-1-carboxylate (**5a**) S20
5.2 (5-Hydroxy-3-phenyl-4,5-dihydro-1*H*-pyrazol-1,5-diyl)bis(phenylmethanone) (**5b**) S22
5.3 (5-Benzoyl-5-hydroxy-3-phenyl-4,5-dihydro-1*H*-pyrazol-1-yl)(4-tolyl)methanone (**5c**) S23
5.4 (5-Benzoyl-5-hydroxy-3-phenyl-4,5-dihydro-1*H*-pyrazol-1-yl)(4-bromophenyl)methanone (**5d**) S25
5.5 (5-Benzoyl-5-hydroxy-3-phenyl-4,5-dihydro-1*H*-pyrazol-1-yl)(thien-2-yl)methanone (**5e**) S27

5.6 (5-Benzoyl-5-hydroxy-3-phenyl-4,5-dihydro-1*H*-pyrazol-1-yl)(furan-2-yl)methanone (**5f**) S28
5.7 1-(5-Benzoyl-5-hydroxy-3-phenyl-4,5-dihydro-1*H*-pyrazol-1-yl)-2-phenylethan-1-one (**5g**) S29
5.8 1-(5-Benzoyl-5-hydroxy-3-phenyl-4,5-dihydro-1*H*-pyrazol-1-yl)-2-methylpropan-1-one (**5h**) S30
5.9 (5-Benzoyl-5-hydroxy-3-phenyl-4,5-dihydro-1*H*-pyrazol-1-yl)(cyclopropyl)methanone (**5i**) S31
5.10 1-(5-Benzoyl-5-hydroxy-3-phenyl-4,5-dihydro-1*H*-pyrazol-1-yl)-2,2-dimethylpropan-1-one (**5j**) S33
5.11 1-(5-Benzoyl-5-hydroxy-3-phenyl-4,5-dihydro-1*H*-pyrazol-1-yl)butan-1-one (**5k**) S34
5.12 (5-Hydroxy-3-(4-methoxyphenyl)-4,5-dihydro-1*H*-pyrazol-1,5-diyl)bis(phenylmethanone) (**5l**) S36
5.13 (3-(4-(*tert*-Butyl)phenyl)-5-hydroxy-4,5-dihydro-1*H*-pyrazol-1,5-diyl)bis(phenylmethanone) (**5m**) S38
5.14 (3-(4-Fluorophenyl)-5-hydroxy-4,5-dihydro-1*H*-pyrazol-1,5-diyl)bis(phenylmethanone) (**5n**) S40
5.15 4-(1,5-Dibenzoyl-5-hydroxy-4,5-dihydro-1*H*-pyrazol-3-yl)benzonitrile (**5o**) S42
5.16 (1-Benzoyl-5-hydroxy-3-phenyl-4,5-dihydro-1*H*-pyrazol-5-yl)(mesityl)methanone (**5p**) S44
5.17 (1-Benzoyl-5-hydroxy-3-phenyl-4,5-dihydro-1*H*-pyrazol-5-yl)(thien-2-yl)methanone (**5q**) S46
5.18 (5-Hydroxy-3-(4-methoxyphenyl)-1-(thiophen-2-carbonyl)-4,5-dihydro-1*H*-pyrazol-5-yl) (mesityl)methanone (**5r**) S47

6 Crystal structures compounds 5a, 5r, and 6a S49

6.1 Crystal structure of *tert*-butyl 5-benzoyl-5-hydroxy-3-phenyl-4,5-dihydro-1*H*-pyrazole-1-carboxylate (**5a**) S50
6.2 Crystal structure of (5-hydroxy-3-(4-methoxyphenyl)-1-(thiophen-2-carbonyl)-4,5-dihydro-1*H*-pyrazol-5-yl) (mesityl)methanone (**5r**) S51
6.3 Crystal structure of phenyl(3-phenyl-1H-pyrazol-5-yl)methanone (**6a**) S52

1 General considerations

All reactions were performed in oven-dried Schlenk vessels via septum and syringe technique under argon. 1,4-Dioxane was dried with the solvent drying and purification system *MBraun MB-SPS-800*. Triethylamine was predried with KOH pellets and then dried with sodium/benzophenone, distilled and stored in a Schlenk flask with KOH pellets under argon.

Phenylglyoxylic acid (**1a**) (98%, Merck/Alfa Aesar), mesitylglyoxylic acid (**1b**) (99%, *ABCR*), 2-thienylglyoxylic acid (**1c**) (98%, *Alfa Aesar*), phenylacetylene (**2a**) (98+%, Alfa Aesar), 4-methoxyphenylacetylene (**2b**) (99%, *Alfa Aesar*), 4-*tert*-butylphenylacetylene (**2c**) (96%, *Acros Organics*), *p*-fluorophenylacetylene (**2d**) (99%, *Alfa Aesar*), 4-ethynylbenzonitrile (**2e**) (94%, *Sigma Aldrich*), *tert*-butyl hydrazinecarboxylate (**4a**) (97%, *Sigma Aldrich*), 4-bromobenzohydrazide (**4d**) (98%, *Acros Organics*), butyrohydrazide (**4k**) (95%, *Alfa Aesar*), oxalylchloride (>98%, *Merck*), copper(I)iodide (*Sigma Aldrich*), and 2-methoxyethanol (*Merck*) were purchased and used without further purification. Benzohydrazide (**4b**), 4-methylbenzohydrazide (**4c**), thiophene-2-carbohydrazide (**4e**), furan-2-carbohydrazide (**4f**), 2-phenylacetohydrazide (**4g**), isobutyrohydrazide (**4h**), cyclopropane-carbohydrazide (**4i**), pivalohydrazide (**4j**), and butyrohydrazide (**4k**) were prepared according to the literature.[1]
Flash chromatography was performed with silica gel 60 (0.040–0.063 mm) (*Fluka*) at 2 bar (compressed air). Prior to chromatography the crude products were adsorbed onto *Celite*® 545 (0.02–0.10 mm) (*Merck* or *Karl Roth*). The reactions were monitored by thin layer chromatography with TLC silica gel 60 F_{254} aluminium foils (*Merck* or *Macherey-Nagel*) and detection was performed by a handheld UV lamp at λ_{exc} = 254 or 365 nm or developing the chromatogram with aqueous $KMnO_4$ solution.

^{1}H, ^{13}C, and 135-DEPT NMR spectra were measured in $CDCl_3$ or $CDCl_3$/tetramethylsilane on *Bruker Avance DRX-500*, *Bruker Avance III-300* or *Bruker Avance DRX-200* spectrometers. The resonances of $CHCl_3$/$CDCl_3$ ($CHCl_3$: ^{1}H δ 7.26, $CDCl_3$: ^{13}C δ 77.0) were set as internal standards (if no $SiMe_4$ was added). HSQC and HMBC spectra were recorded on a *Bruker Avance III-300*. Electron ionization (EI) mass spectra were recorded on a *Finnigan MAT 8200* spectrometer. GC–MS analysis was performed with the GC/MS systems *GC2010* and *GC/MS-QP2010S* (*Shimadzu*). IR Spectra were recorded on a *Bruker Vector 22 FT-IR* (KBr Pellets) or *Shimadzu IRAffinity* (ATR). The intensities were labelled as s (strong), m (middle), w (weak). Combustion analyses were determined on a *Perkin Elmer Series II Analyser 2400* (C,H,N-Analyse) in the microanalytic laboratory of the Institut für Pharmazeutische und Medizinische Chemie, Heinrich-Heine-Universität Düsseldorf. Melting points (uncorrected) were determined with melting point microscope (*Reichert Thermovar*), a *PeakTech 6000A* DC *power adaptor*, and digital thermometer *D2400* (*Norma*).

X-ray structure analyses were performed in the group of Prof. Dr. *W. Frank*, Institut für Anorganische Chemie und Strukturchemie, Heinrich-Heine-Universität Düsseldorf, on a *Stoe IPDS* (opus), an Xcalibur EOS (no) and an *Oxford 4-Circle Diffractometer* with an *EOS plane detector* (exp).

---

[1] Kaushik, D.; Khan, S. A.; Chawla, G.; Kumar, S. *Eur. J. Med. Chem.* **2010**, *45*, 3943-3949. DOI: 10.1016/j.ejmech.2010.05.049

2 Optimization studies

2.1 Optimization of 1,4-diphenylbut-3-yn-1,2-dione (3a)

In an oven-dried Schlenk flask with magnetic stir bar and screw cap were placed phenylglyoxylic acid (**1a**, 150 mg, 1.00 mmol or 765 mg, 5.00 mmol) and dry 1,4-dioxane (2.5 mL or 25 mL) under argon (degassing by flushing with argon through a syringe needle for 5 min). Then, oxalyl chloride (0.09 mL, 1.00 mmol or 0.49 mL, 5.00 mmol) was added dropwise at room temperature (external water bath) by syringe through the septum to the reaction mixture and stirring was continued for 15 min. Then, the reaction mixture was stirred at 50 °C (preheated oil bath) for 4 h. After the mixture had cooled to room temperature CuI (10 mg, 0.05 mmol or 49 mg, 0.25 mmol), phenylacetylene (**2a**) (0.11 mL, 1.00 mmol or 0.58 mL, 5.00 mmol), and dry triethylamine (0.42 mL, 3.00 mmol or 2.1 mL, 15.0 mmol) were successively added. Stirring at room temperature (external water bath) was continued for 15 to 24 h (for details see Table S1). After complete conversion (monitored by TLC) and cooling to room temperature deionized water (5 mL) was added and the mixture was extracted with dichloromethane (4 × 10 mL). The combined organic phases were dried (anhydrous sodium sulfate) and the solvents were removed in vacuo. The crude product was adsorbed on celite© and purified by flash chromatography on silica gel (petroleum ether 40–60 °C/ethyl acetate 50:1) to give analytically pure 1,4-diphenylbut-3-yn-1,2-dione (**3a**) as yellow oil.

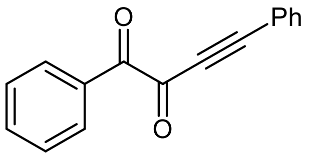

$C_{16}H_{10}O_2$

[234.25]

^{1}H NMR ($CDCl_3$, 500 MHz): δ 7.39-7.43 (m, 2 H), 7.48-7.56 (m, 3 H), 7.63-7.70 (m, 3 H), 8.07-8.10 (m, 2 H). ^{13}C NMR ($CDCl_3$, 125 MHz): δ 87.0 (C_{quat}), 99.1 (C_{quat}), 119.1 (C_{quat}), 128.7 (CH), 128.9 (CH), 130.5 (CH), 131.5 (C_{quat}), 131.7 (CH), 133.6 (CH), 134.9 (CH), 178.5 (C_{quat}), 188.4 (C_{quat}). EI+MS (*m/z* (%)): 234 (M^+, 0.4), 206 ($(M-CO)^+$, 3), 178 ($(M-C_2O_2)^+$, 31), 129 ($(M-C_7H_5O)^+$, 71), 105 ($C_7H_5O^+$, 100), 85 (11), 77 ($C_6H_5^+$, 38), 75 (11), 71 (15), 57 (13). IR: $\tilde{\nu}$ [cm^{-1}]: 3065 (w), 2927 (w), 2191 (s), 1656 (s), 1595 (m), 1489 (w), 1449 (m), 1249 (m), 1182 (w), 1108 (s), 1025 (w), 1000 (w), 924 (m), 816 (w), 778 (m), 759 (m), 738 (m), 685 (s), 611 (w), 538 (w). Anal. calcd. for $C_{16}H_{10}O_2$ (234.3): C 82.04, H 4.30; Found: C 82.13, H 4.31.
GC-MS (*m/z* (%)): R_t = 14.87 min. 234 (M^+, 6), 178 ($(M-C_2O_2)^+$, 21), 129 ($(M-C_7H_5O)^+$, 57), 105 ($C_7H_5O^+$, 100), 77 ($C_6H_5^+$, 92), 75 (40), 74 (17), 51 (66), 50 (20).

Table S1: Optimization of the activation–alkynylation synthesis of 1,4-diphenylbut-3-yne-1,2-dione (**3a**).

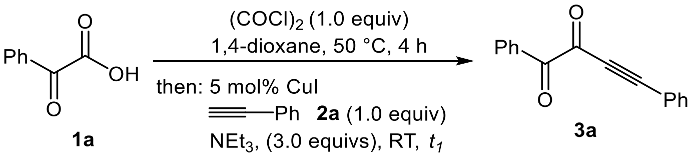

Entry	scale	c_0 (**1a**)	t_1	1,4-diphenylbut-3-yne-1,2-dione (**3a**)
1[1]	5.00 mmol	0.2 M	24 h	61%
2[2]	5.00 mmol	0.2 M	24 h	63%
3[2]	1.00 mmol	0.2 M	24 h	65%
4[2]	1.00 mmol	0.2 M	20 h	65%
5[2]	1.00 mmol	0.2 M	15 h	65%
**6[2]**	**1.00 mmol**	**0.4 M**	**15 h**	**76%**
7[2,3]	1.00 mmol	0.4 M	15 h	70%

[1]Triethylamine dried with sodium. [2]Triethylamine (purest, Appli Chem GmbH, 99%), dried with KOH. [3]Performed in a microwave vessel.

2.2 Synthesis of *tert*-butyl 5-benzoyl-5-hydroxy-3-phenyl-4,5-dihydro-1*H*-pyrazole-1-carboxylate (5a)

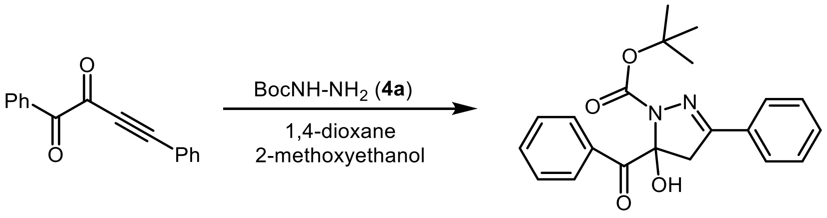

In a dry screw-cap Schlenk vessel with a magnetic stirring bar 1,4-diphenylbut-3-yne-1,2-dione (**3a**, 238 mg, 1.02 mmol) was dissolved in 1,4-dioxane (5.1 mL) under argon. Then, *tert*-butyl carbazate (**4a**, 136 mg, 1.02 mmol) and 2-methoxyethanol (1.02 mL) were added. The reaction mixture was heated at 100 °C (preheated oil bath) for 24 h. After cooling to room temperature the solvents were removed in vacuo and the crude product was adsorbed on celite® and purified by flash chromatography on silica gel (petroleum ether/ethyl acetate 20:1) to give *tert*-butyl 5-benzoyl-5-hydroxy-3-phenyl-4,5-dihydro-1*H*-pyrazole-1-carboxylate (**5a**, 310 mg, 83%) as colorless solid, Mp 155 °C; R_f (petroleum ether/ethyl acetate 20:1): 0.05.

^{1}H NMR ($CDCl_3$, 300 MHz): δ 1.23 (s, 7 H), 1.46 (s, 2 H), 3.48 (d, J = 18.3 Hz, 1 H), 3.76 (d, J = 18.3 Hz, 1 H), 5.63 (br, 1 H), 7.37-7.49 (m, 5 H), 7.53-7.66 (m, 1 H), 7.72-7.87 (m, 2 H), 7.87-

7.95 (m, 2 H). ^{13}C NMR ($CDCl_3$, 75 MHz): δ 27.7 (CH_3), 47.7 (CH_2), 83.2 (C_{quat}), 90.5 (C_{quat}), 126.9 (CH), 128.7 (CH), 129.1 (CH), 129.2 (CH), 130.4 (CH), 130.8 (C_{quat}), 131.1 (C_{quat}), 134.4 (CH), 149.7 (C_{quat}), 150.9 (C_{quat}), 194.2 (C_{quat}). MS (EI), *m/z*: 261 ($(M – PhCO)^+$, 5), 248 ($(M – {}^tBuOCO_2H)^+$, 16), 205 (9), 162 (11), 161 ($(M – PhCO – CH_2{=}CMe_2 – CO_2)^+$, 100), 105 ($PhCO^+$, 23), 77 ($C_6H_5^+$, 16), 57 ($C_4H_9^+$, 15). IR (ATR), $\tilde{\nu}$ [cm^{-1}]: 3030 (w), 1721 (s), 1649 (m), 1622 (w), 1533 (s), 1493 (w), 1410 (m), 1383 (w), 1364 (w), 1341 (s), 1294 (m), 1267 (m), 1246 (s), 1233 (s), 1188 (m), 1153 (s), 1134 (m), 1072 (w), 1042 (s), 993 (w), 982 (w), 932 (w), 872 (s), 816 (m), 756 (s), 691 (s), 610 (m). Anal. calcd. for $C_{21}H_{22}N_2O_4$ (366.4): C 68.84, H 6.05, N 7.65; Found: C 68.87, H 6.08, N 7.66.

2.3 Optimization of (5-hydroxy-3-phenyl-4,5-dihydro-1*H*-pyrazole-1,5-diyl)bis(phenylmethanone) (5b)

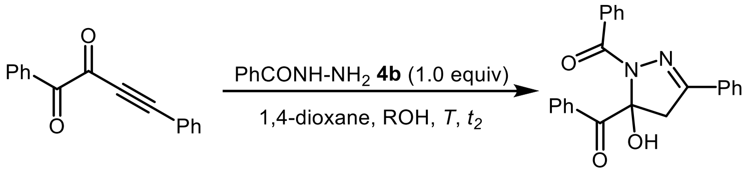

3a **5b**

In a dry screw-cap microwave vessel with a magnetic stirring bar ynedione **3a** (47 mg, 0.2 mmol), benzohydrazide (**4b**), 1,4-dioxane (1.0 mL) and 2-methoxyethanol (0.2 mL) were added and the reaction mixture was placed in the microwave cavity or the preheated oil bath at the temperature and for the time indicated (for experimental details, see Table S2). The conversion was monitored by GC–MS. Only for full conversion and after cooling to room temperature and work up (vide supra) the residue was chromatographed on silica gel (petroleum ether/ethyl acetate 5:1) and isolated as colorless solid, Mp 152 °C; R_f (petroleum ether/ethyl acetate 5:1): 0.15 (vide infra for spectroscopic and analytical data).

GC-MS (m/z (%)): R_t: 17,30 min. 248 ($(M\text{-}C_7H_5O\text{-}H_2O)^+$, 34), 115 (10), 112 (14), 105 ($C_7H_5O^+$, 48), 89 (11), 77 ($C_6H_5^+$, 100), 51 (50), 50 (13). R_t: 20,17 min. 248 ($(M\text{-}C_7H_5O\text{-}H_2O)^+$, 22), 247 (13), 145 (44), 105 ($C_7H_5O^+$, 13), 104 (100), 103 (27), 102 (10), 90 (12), 89 (21), 78 (11), 77 ($C_6H_5^+$, 63), 76 (32), 68 (19), 63 (19), 51 (36), 50 (17). R_t: 22,43 min. 352 ($(M\text{-}H_2O)^+$, 8), 248 ($(M\text{-}C_7H_5O\text{-}H_2O)^+$, 1), 105 ($C_7H_5O^+$, 100), 77 ($C_6H_5^+$, 71), 51 (18). R_t: 24,25 min. 105 ($C_7H_5O^+$, 100), 77 ($C_6H_5^+$, 45), 51 (19). R_t: 25,55 min. 105 ($C_7H_5O^+$, 100), 77 ($C_6H_5^+$, 52), 51 (14).

Table S2: Optimization of the cyclization step of 1,5-diacyl-5-hydroxypyrazoline **5b**.[a]

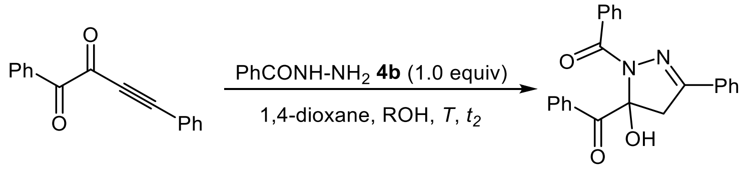

3a **5b**

Entry	ROH	**4b**	T	t_2	1,5-diacyl-5-hydroxypyrazoline **5b**	
	[mL]	[mg] (mmol)	[°C]	[min]	GC-Conversion	[mg] (%)[b]
1[c]	2-methoxyethanol (0.2)	27.2 (0.20)	100	60	incomplete conversion[d]	(n.i.)
2[c]	2-methoxyethanol (0.2)	27.2 (0.20)	150	60	incomplete conversion[d]	(n.i.)
3[c]	2-methoxyethanol (0.2)	41.0 (0.30)	150	60	complete conversion[e]	(n.i.)
4[c]	2-methoxyethanol (0.2)	34.0 (0.25)	150	60	complete conversion[d]	(n.i.)
5[c]	2-methoxyethanol (0.2)	33.0 (0.24)	150	60	complete conversion[d]	(n.i.)
6[c]	2-methoxyethanol (0.2)	30.0 (0.22)	150	60	incomplete conversion[d]	(n.i.)
7[c]	2-methoxyethanol (0.2)	33.0 (0.24)	150	30	complete conversion[d]	(n.i.)
8[c]	2-methoxyethanol (0.2)	33.0 (0.24)	150	15	complete conversion[d]	(n.i.)
9[c]	2-methoxyethanol (0.2)	33.0 (0.24)	150	5	incomplete conversion[d]	(n.i.)
10[c]	2-methoxyethanol (0.2)	33.0 (0.24)	100	10	incomplete conversion[d]	(n.i.)
11[c]	2-methoxyethanol (0.2)	33.0 (0.24)	125	10	incomplete conversion[d]	(n.i.)
**12[c,e]**	**2-methoxyethanol (0.2)**	136.4 (1.20)	**175**	**5**	**full conversion[d]**	**348 (94)**
13[c,e]	ethylene glycol (0.2)	136.4 (1.20)	175	5	full conversion[d]	356 (96)
14[c,e]	ethanol (0.2)	136.4 (1.20)	175	5	full conversion[d]	322 (87)
**15[f]**	**2-methoxyethanol (0.2)**	136.4 (1.20)	**175**	**5**	**full conversion[d]**	**333 (90)**
16[e,g]	2-methoxyethanol (0.2)	136.4 (1.20)	175	5	full conversion[d]	345 (93)

[a] c_0(**3a**) = 0.17 M; 1,4-dioxane (1.0 mL). [b] Isolated yield (n.i. = not isolated). [c] Dielectric heating in a microwave cavity (T is the set temperature and t_2 is the hold time). [d] As monitored by GC–MS. [e] On a 1.00 mmol scale (**3a**). [f] On a 1.00 mmol scale (**3a**), c_0(**3a**) = 0.34 M; c_0(**4b**) = 0.40 M. 1,4-dioxane (1.0 mL). [g] Conductive heating in an oil bath at preheated temperature T.

2.4 Optimization of consecutive three-component synthesis of (5-hydroxy-3-phenyl-4,5-dihydro-1*H*-pyrazole-1,5-diyl)bis(phenyl-methanone) (5b)

$$\mathbf{1a} \xrightarrow[\substack{\text{then: 5 mol\% CuI, } \mathbf{2a} \text{ (1.0 equiv)} \\ NEt_3\text{, (3.0 equivs), RT, } t_1 \\ \text{then: } \mathbf{4b} \text{ (1.0 equiv), } MeOCH_2CH_2OH\text{, 175 °C, } t_2}]{\substack{(COCl)_2 \text{ (1.0 equiv)} \\ \text{1,4-dioxane, 50 °C, 4 h}}} \mathbf{5b}$$

In an oven-dried Schlenk flask with magnetic stirring bar and screw cap were placed phenylglyoxylic acid (**1a**, 150 mg, 1.00 mmol) and dry 1,4-dioxane (2.5 mL) under argon. Then, oxalyl chloride (0.09 mL, 1.00 mmol) was added dropwise at room temperature (external water bath) to the solution. Then, the reaction mixture was stirred at 50 °C (preheated oil bath) for 4 h. After the mixture had cooled to room temperature CuI (10 mg, 0.05 mmol), phenylacetylene (**2a**, 0.11 mL, 1.00 mmol), and dry triethylamine (0.42 mL, 3.00 mmol) were successively added and stirring at room temperature (external water bath) was continued for 15 h. Then, hydrazide **4b** (136.4 mg, 1.20 mmol) and 2-methoxyethanol (1.0 mL) were added and the reaction mixture was placed in the microwave cavity or the preheated oil bath at the temperature and for the time indicated (for experimental details, see Table S3). Only for full conversion and after cooling to room temperature and work up (vide supra) the residue was chromatographed on silica gel (petroleum ether/ethyl acetate 5:1) and isolated as colorless solid, Mp 152 °C; R_f (petroleum ether/ethyl acetate 5:1): 0.15 (vide infra for spectroscopic and analytical data).

Table S3: Optimization of the consecutive three-component synthesis of 1,5-diacyl-5-hydroxypyrazoline **5b**.

$$\mathbf{1a} \xrightarrow[\substack{\text{then: 5 mol\% CuI, } \mathbf{2a} \text{ (1.0 equiv)} \\ NEt_3\text{, (3.0 equivs), RT, 15 h} \\ \text{then: } \mathbf{4b} \text{ (1.0 equiv), } MeOCH_2CH_2OH\text{, 175 °C, } t_2}]{\substack{(COCl)_2 \text{ (1.0 equiv)} \\ \text{1,4-dioxane, 50 °C, 4 h}}} \mathbf{5b}$$

Entry	c_0(**1a**)	t_2 [min]	1,5-diacyl-5-hydroxypyrazoline **5b**, yield [mg] (%)[a]
1[b]	0.4 M	5	137 (37)
2[b]	0.25 M	5	119 (32)
3[b]	0.25 M	10	130 (35)
4[c]	0.4 M	10	no product formation[d]
5[e,f]	0.4 M	5	no product formation[d]
6[e]	0.4 M	10	237 (64)
7[e]	0.4 M	20	255 (69)
**8[e]**	**0.4 M**	**30**	**289 (78)**
9[e]	0.4 M	45	292 (79)

[a] Isolated yield. [b] Dielectric heating in a microwave cavity (*T* is set to 175 °C and t_2 is the hold time). [c] Dielectric heating in a microwave cavity (*T* is set to 150 °C and t_2 is the hold time). [d] As monitored by GC-MS. [e] Conductive heating in an oil bath at preheated temperature *T* = 175 °C. [f] 2.00 equivs of NEt_3 were added.

3 Three-component synthesis of 1,5-diacyl-5-hydroxypyrazolines 5

3.1 General procedure (GP)

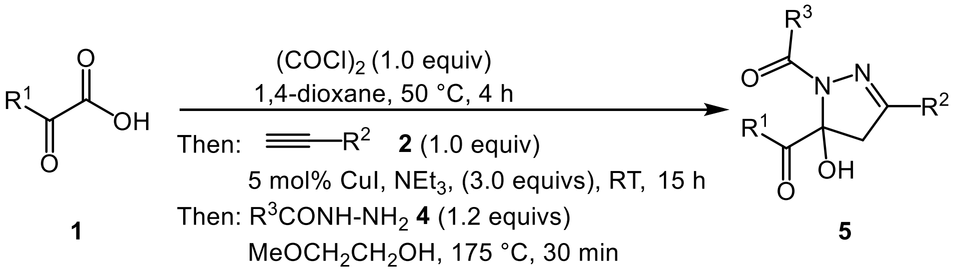

In an oven-dried Schlenk flask with magnetic stirring bar and screw cap were placed glyoxylic acid **1** (1.00 mmol) and dry 1,4-dioxane (2.5 mL) under argon (for experimental details see Table S4). Then, oxalyl chloride (0.09 mL, 1.00 mmol) was added dropwise at room temperature (external water bath) and the reaction mixture was stirred at 50 °C (preheated oil bath) for 4 h. After the mixture had cooled to room temperature CuI (10 mg, 0.05 mmol), alkyne **2** (1.00 mmol), and dry triethylamine (0.42 mL, 3.00 mmol) were successively added. Stirring at room temperature (external water bath) was continued for 15 h. Then, hydrazide **3** (1.20 mmol) and 2-methoxyethanol (1.0 mL) were added and the reaction mixture was stirred at 175 °C (preheated oil bath) for 30 min. After complete conversion (monitored by TLC) and cooling to room temperature deionized water (5 mL) was added and the mixture was extracted with dichloromethane (4 × 5 mL). The combined organic phases were dried with anhydrous sodium sulfate and the solvents were removed in vacuo. The crude product was adsorbed on celite© and purified by flash chromatography on silica gel (petrolether 40–60 °C/ethyl acetate) to give analytically pure 1,5-diacyl-5-hydroxypyrazolines **5**.

Table S4: Experimental details of the three-component synthesis of 1,5-diacyl-5-hydroxypyrazolines **5b–r**.

Entry	Glyoxylic acid **1**	Alkyne **2**	Hydrazide **4**	1,5-Diacyl-5-hydroxypyrazolines **5b–r**
1	150 mg (1.00 mmol) of **1a**	0.11 mL (1.00 mmol) of **2a**	163 mg (1.20 mmol) of **4b**	291 mg (78%) of **5b**
2	150 mg (1.00 mmol) of **1a**	0.11 mL (1.00 mmol) of **2a**	180 mg (1.20 mmol) of **4c**	209 mg (55%) of **5c**
3	150 mg (1.00 mmol) of **1a**	0.11 mL (1.00 mmol) of **2a**	232 mg (1.20 mmol) of **4d**	185 mg (41%) of **5d**
4	150 mg (1.00 mmol) of **1a**	0.11 mL (1.00 mmol) of **2a**	170 mg (1.20 mmol) of **4e**	268 mg (71%) of **5e**
5	150 mg (1.00 mmol) of **1a**	0.11 mL (1.00 mmol) of **2a**	151 mg (1.20 mmol) of **4f**	241 mg (67%) of **5f**
6	150 mg (1.00 mmol) of **1a**	0.11 mL (1.00 mmol) of **2a**	180 mg (1.20 mmol) of **4g**	228 mg (59%) of **5g**
7	150 mg (1.00 mmol) of **1a**	0.11 mL (1.00 mmol) of **2a**	123 mg (1.20 mmol) of **4h**	222 mg (66%) of **5h**

Entry	Glyoxylic acid **1**	Alkyne **2**	Hydrazide **4**	1,5-Diacyl-5-hydroxypyrazolines **5b–r**
8	150 mg (1.00 mmol) of **1a**	0.11 mL (1.00 mmol) of **2a**	120 mg (1.20 mmol) of **4i**	232 mg (69%) of **5i**
9	150 mg (1.00 mmol) of **1a**	0.11 mL (1.00 mmol) of **2a**	139 mg (1.20 mmol) of **4j**	203 mg (58%) of **5j**
10	150 mg (1.00 mmol) of **1a**	0.11 mL (1.00 mmol) of **2a**	129 mg (1.20 mmol) of **4k**	111 mg (33%) of **5k**
11	150 mg (1.00 mmol) of **1a**	0.13 mL (1.00 mmol) of **2b**	163 mg (1.20 mmol) of **4b**	220 mg (55%) of **5l**
12	150 mg (1.00 mmol) of **1a**	0.19 mL (1.00 mmol) of **2c**	163 mg (1.20 mmol) of **4b**	293 mg (69%) of **5m**
13	150 mg (1.00 mmol) of **1a**	121 mg (1.00 mmol) of **2d**	163 mg (1.20 mmol) of **4b**	256 mg (66%) of **5n**
14	150 mg (1.00 mmol) of **1a**	131 mg (1.00 mmol) of **2e**	163 mg (1.20 mmol) of **4b**	113 mg (29%) of **5o**
15	194 mg (1.00 mmol) of **1b**	0.11 mL (1.00 mmol) of **2a**	163 mg (1.20 mmol) of **4b**	194 mg (47%) of **5p**
16	159 mg (1.00 mmol) of **1c**	0.11 mL (1.00 mmol) of **2a**	163 mg (1.20 mmol) of **4b**	275 mg (73%) of **5q**
17	194 mg (1.00 mmol) of **1b**	0.13 mL (1.00 mmol) of **2b**	170 mg (1.20 mmol) of **4e**	172 mg (38%) of **5r**

3.2 (5-Hydroxy-3-phenyl-4,5-dihydro-1*H*-pyrazol-1,5-diyl)bis(phenylmethanone) (5b)

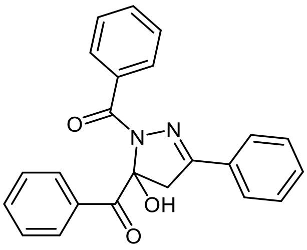

According to the GP and chromatography on silica gel (petroleum ether/ethyl acetate 5:1) compound **5b** (291 mg, 78%) was obtained as colorless solid, Mp 152 °C; R_f (petroleum ether/ethyl acetate 5:1): 0.15. ^{1}H NMR ($CDCl_3$, 300 MHz): δ 3.54 (d, J = 18.5 Hz, 1 H), 3.76 (d, J = 18.5 Hz, 1 H), 5.60-6.08 (br, 1 H), 7.36-7.62 (m, 9 H), 7.72-7.83 (m, 2 H), 7.90-8.05 (m, 4 H). ^{13}C NMR ($CDCl_3$, 75 MHz): δ 45.6 (CH_2), 92.2 (C_{quat}), 126.9 (CH), 127.8 (CH), 128.9 (CH)*, 129.0 (CH), 130.2 (CH), 130.7 (C_{quat}), 130.9 (CH), 131.7 (CH), 131.8 (C_{quat}), 132.9 (C_{quat}), 133.9 (CH), 153.1 (C_{quat}), 166.7 (C_{quat}), 193.4 (C_{quat}); *broadened signal. MS (EI), *m/z*: 352 ($(M – H_2O)^+$, 2), 266 (11), 265 ($(M – C_7H_5O)^+$, 59), 248 ($(M – C_7H_5O – H_2O)^+$, 20), 105 ($C_7H_5O^+$, 100), 77 ($C_6H_5^+$, 34). IR (ATR), $\tilde{\nu}$ [cm^{-1}]: 3333 (w), 1697 (m), 1626 (m), 1612 (m), 1566 (w), 1450 (m), 1427 (m), 1339 (m), 1315 (w), 1254 (w), 1202 (m), 1180 (m), 1113 (m), 1057 (w), 1028 (w), 922 (w), 895 (w), 866 (m), 845 (w), 791 (w), 762 (m), 708 (s), 689 (s), 669 (m), 627 (w). Anal. calcd. for $C_{23}H_{18}N_2O_3$ (370.4): C 74.58, H 4.90, N 7.56; Found: C 74.67, H 5.07, N 7.79.

3.3 (5-Benzoyl-5-hydroxy-3-phenyl-4,5-dihydro-1*H*-pyrazol-1-yl)(4-tolyl)methanone (5c)

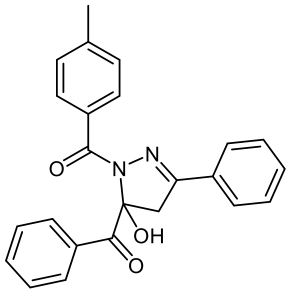

According to the GP and chromatography on silica gel (petroleum ether/ethyl acetate 5:1) compound **5c** (209 mg, 55%) was obtained as light yellow solid, Mp 147 °C; R_f (petroleum ether/ethyl acetate 5:1): 0.17. ^{1}H NMR ($CDCl_3$, 300 MHz): δ 2.41 (s, 3 H), 3.52 (d, *J* = 18.5 Hz, 1 H), 3.74 (d, *J* = 18.5 Hz, 1 H), 5.82 (br, 1 H), 7.25 (d, *J* = 8.4 Hz, 2 H), 7.36-7.50 (m, 5 H), 7.51-7.61 (m, 1 H), 7.70-7.84 (m, 2 H), 7.88-8.00 (m, 4 H). ^{13}C NMR ($CDCl_3$, 75 MHz): δ 21.7 (CH_3), 45.6 (CH_2), 92.4 (C_{quat}), 127.0 (CH), 128.6 (CH), 129.0 (CH)*, 129.2 (CH), 130.1 (C_{quat}), 130.5 (CH), 130.9 (CH), 131.0 (C_{quat}), 132.1 (C_{quat}), 134.0 (CH), 142.4 (C_{quat}), 153.0 (C_{quat}), 166.7 (C_{quat}), 193.7 (C_{quat}); *broadened signal. MS (EI), *m/z*: 366 ($(M - H_2O)^+$, 7), 279 ($(M - C_7H_5O)^+$, 44), 248 ($(M - C_7H_7O - H_2O)^+$, 7), 119 ($C_8H_7O^+$, 100), 105 ($C_7H_5O^+$, 9), 91 (21), 77 ($C_6H_5^+$, 7). IR (ATR), $\tilde{\nu}$ [cm^{-1}]: 3387 (w), 3362 (w), 1701 (s), 1632 (m), 1607 (m), 1597 (m), 1558 (w), 1512 (w), 1449 (m), 1422 (s), 1404 (m), 1358 (m), 1337 (s), 1273 (w), 1256 (w), 1204 (s), 1182 (s), 1111 (s), 1061 (m), 1026 (w), 1001 (w), 920 (m), 899 (w), 847 (w), 827 (w), 785 (m), 760 (m), 743 (s), 706 (s), 689 (s), 675 (m), 646 (w). Anal. calcd. for $C_{24}H_{20}N_2O_3$ (384.4): C 74.98, H 5.24, N 7.29; Found: C 75.19, H 5.42, N 7.26.

3.4 (5-Benzoyl-5-hydroxy-3-phenyl-4,5-dihydro-1*H*-pyrazol-1-yl)(4-bromophenyl)methanone (5d)

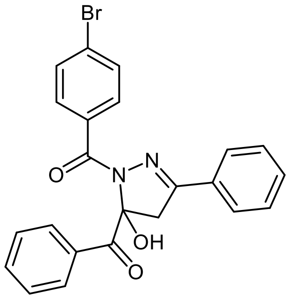

According to the GP and chromatography on silica gel (petroleum ether/ethyl acetate 5:1) compound **5d** (185 mg, 41%) was obtained as beige solid, Mp 164 °C; R_f (petroleum ether/ethyl acetate 5:1): 0.19. ^{1}H NMR ($CDCl_3$, 300 MHz): δ 3.54 (d, *J* = 18.5 Hz, 1 H), 3.75 (d, *J* = 18.5 Hz, 1 H), 4.7-6.4 (br, 1 H), 7.38-7.51 (m, 5 H), 7.53-7.61 (m, 3 H), 7.71-7.79 (m, 2 H), 7.84-7.97 (m, 4 H). ^{13}C NMR ($CDCl_3$, 75 MHz): δ 46.0 (CH_2), 92.6 (C_{quat}), 127.0 (C_{quat}), 127.3 (CH), 129.30 (CH), 129.32 (CH), 129.4 (CH), 130.9 (C_{quat}), 131.4 (CH), 131.5 (CH), 132.06 (C_{quat}), 132.10 (C_{quat}), 132.2 (CH), 134.5 (CH), 153.9 (C_{quat}), 166.0 (C_{quat}), 193.7 (C_{quat}). MS (EI), *m/z*: 432 ($(^{81}Br\text{-}M - H_2O)^+$, 0.5), 430 ($(^{79}Br\text{-}M - H_2O)^+$, 0.6), 345 ($(^{81}Br\text{-}M - C_7H_5O)^+$, 25), 343 ($(^{79}Br\text{-}M - C_7H_5O)^+$, 25), 303 (15), 301 (15), 249 (18), 248 ($(M - C_7H_4BrO - H_2O + H)^+$, 100), 247 (10), 185 (51), 183 (53), 171 (53), 157 ($C_6H_4{}^{81}Br^+$, 10), 155 ($C_6H_4{}^{79}Br^+$, 11), 105 ($C_7H_5O^+$, 42), 77 ($C_6H_5^+$, 32). IR (ATR), $\tilde{\nu}$ [cm^{-1}]: 3302 (w), 1705 (m), 1612 (m), 1585 (w), 1557 (w), 1439 (m), 1342 (m), 1256 (m), 1200 (w), 1182 (w), 1134 (w), 1109 (m), 1070 (w), 1036 (w), 1007 (m), 980 (w), 934 (w), 891 (w), 849 (w), 829 (m), 781 (w), 752 (m), 714 (m), 687 (s), 665 (s), 640 (m), 621 (m). Anal. calcd. for $C_{23}H_{17}BrN_2O_3$ (449.3): C 61.48, H 3.81, N 6.23; Found: C 61.25, H 4.00, N 6.08.

3.5 (5-Benzoyl-5-hydroxy-3-phenyl-4,5-dihydro-1*H*-pyrazol-1-yl)(thien-2-yl)methanone (5e)

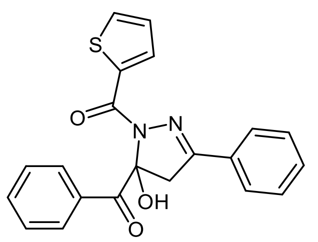

According to the GP and chromatography on silica gel (petroleum ether/ethyl acetate 5:1) compound **5e** (268 mg, 71%) was obtained as light yellow solid, Mp 162 °C; R_f (petroleum ether/ethyl acetate 5:1): 0.16. ^{1}H NMR ($CDCl_3$, 300 MHz): δ 3.54 (d, *J* = 18.4 Hz, 1 H), 3.77 (d, *J* = 18.5 Hz, 1 H), 5.60-6.15 (br, 1 H), 7.13 (dd, *J* = 5.0 Hz, *J* = 3.8 Hz, 1 H), 7.40 (t, *J* = 7.7 Hz, 2 H), 7.48-7.62 (m, 4 H), 7.67 (dd, *J* = 4.9 Hz, *J* = 1.3 Hz, 1 H), 7.83-8.00 (m, 4 H), 8.15 (dd, *J* = 3.9 Hz, *J* = 1.4 Hz, 1 H). ^{13}C NMR ($CDCl_3$, 75 MHz): δ 46.0 (CH_2), 91.9 (C_{quat}), 127.1 (CH), 127.2 (CH), 129.12 (CH), 129.14 (CH), 129.2 (CH), 130.8 (C_{quat}), 131.2 (CH), 131.7 (C_{quat}), 133.8 (C_{quat}), 134.2 (CH), 134.5 (CH), 135.8 (CH), 153.2 (C_{quat}), 159.7 (C_{quat}), 193.3 (C_{quat}). MS (EI), *m/z*: 358 ((M – $H_2O)^+$, 1), 272 (12), 271 ((M – $C_7H_5O)^+$, 71), 111 ($C_5H_3OS^+$, 100), 105 ($C_7H_5O^+$, 6), 77 ($C_6H_5^+$, 8). IR (ATR), $\tilde{\nu}$ [cm^{-1}]: 3316 (w), 2922 (m), 1695 (m), 1609 (m), 1595 (m), 1512 (m), 1437 (s), 1410 (m), 1327 (m), 1304 (w), 1275 (w), 1252 (w), 1206 (s), 1186 (m), 1115 (s), 1059 (w), 1043 (m), 937 (w), 920 (m), 901 (w), 858 (m), 826 (m), 762 (m), 729 (s), 704 (s), 689 (s), 677 (m), 623 (w). Anal. calcd. for $C_{21}H_{16}N_2O_3S$ (376.4): C 67.00, H 4.28, N 7.44; Found: C 67.21, H 4.45, N 7.19.

3.6 (5-Benzoyl-5-hydroxy-3-phenyl-4,5-dihydro-1*H*-pyrazol-1-yl)(furan-2-yl)methanone (5f)

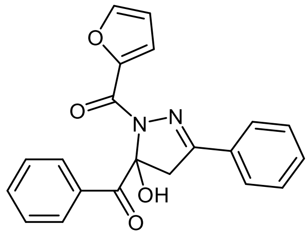

According to the GP and chromatography on silica gel (petroleum ether/ethyl acetate 4:1) compound **5f** (241 mg, 67%) was obtained as beige solid, Mp 183 °C; R_f (petroleum ether/ethyl acetate 4:1): 0.22. ^{1}H NMR ($CDCl_3$, 300 MHz): δ 3.51 (d, *J* = 18.5 Hz, 1 H), 3.74 (d, *J* = 18.5 Hz, 1 H), 5.78 (br, 1 H), 6.58 (dd, *J* = 3.5 Hz, *J* = 1.7 Hz, 1 H), 7.34-7.44 (m, 2 H), 7.45-7.59 (m, 4 H), 7.59-7.65 (m, 1 H), 7.74-7.79 (m, 1 H), 7.80-7.86 (m, 2 H), 7.86-7.94 (m, 2 H). ^{13}C NMR ($CDCl_3$, 75 MHz): δ 45.9 (CH_2), 92.4 (C_{quat}), 112.2 (CH), 120.9 (CH), 127.3 (CH), 129.3 (CH), 129.4 (CH), 129.5 (CH), 131.1 (C_{quat}), 131.4 (CH), 132.1 (C_{quat}), 134.4 (CH), 145.6 (C_{quat}), 146.7 (CH), 154.0 (C_{quat}), 156.5 (C_{quat}), 193.5 (C_{quat}). MS (EI), *m/z*: 342 ((M – $H_2O)^+$, 2), 256 ((M – $C_5H_3O_2)^+$, 16), 255 ((M – $C_7H_5O)^+$, 100), 105 ($C_7H_5O^+$, 8), 95 (85), 77 ($C_6H_5^+$, 11). IR (ATR), $\tilde{\nu}$ [cm^{-1}]: 1678 (m), 1603 (s), 1551 (m), 1466 (s), 1441 (s), 1339 (m), 1240 (m), 1223 (m), 1209 (m), 1018 (w), 928 (m), 851 (m), 816 (m), 795 (m), 768 (s), 748 (m), 710 (s), 692 (s), 667 (m), 633 (m). Anal. calcd. for $C_{21}H_{16}N_2O_4$ (360.4): C 69.99, H 4.48, N 7.77; Found: C 70.12, H 4.18, N 7.75.

3.7 1-(5-Benzoyl-5-hydroxy-3-phenyl-4,5-dihydro-1*H*-pyrazol-1-yl)-2-phenylethan-1-one (5g)

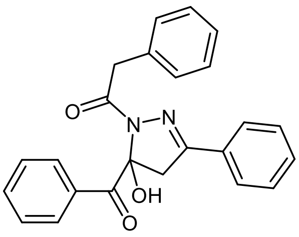

According to the GP and chromatography on silica gel (petroleum ether/ethyl acetate 5:1) compound **5g** (228 mg, 59%) was obtained as beige solid, Mp 110 °C; R_f (petroleum ether/ethyl acetate 5:1): 0.15. ^{1}H NMR ($CDCl_3$, 300 MHz): δ 3.37 (d, J = 18.5 Hz, 1 H), 3.58 (d, J = 18.5 Hz, 1 H), 3.87 (d, J = 14.0 Hz, 1 H), 4.09 (d, J = 14.0 Hz, 1 H), 5.61 (br, 1 H), 7.09-7.21 (m, 7 H), 7.35-7.45 (m, 4 H), 7.54-7.62 (m, 2 H), 7.67-7.76 (m, 2 H). ^{13}C NMR ($CDCl_3$, 75 MHz): δ 41.5 (CH_2), 46.9 (CH_2), 91.0 (C_{quat}), 127.15 (CH), 127.17 (CH), 128.8 (CH), 129.1 (CH), 129.26 (CH), 129.30 (CH), 129.8 (CH), 131.16 (C_{quat}), 131.22 (CH), 131.7 (C_{quat}), 134.2 (CH), 134.3 (C_{quat}), 152.8 (C_{quat}), 169.9 (C_{quat}), 193.7 (C_{quat}). MS (EI), *m/z*: 366 ($(M - H_2O)^+$, 1.2), 279 ($(M - C_7H_5O)^+$, 41), 162 (11), 161 ($(M - C_7H_5O - C_8H_7O + H)^+$, 100), 105 ($C_7H_5O^+$, 8), 91 ($C_7H_7^+$, 18), 77 ($C_6H_5^+$, 10). IR (ATR), $\tilde{\nu}$ [cm^{-1}]: 3406 (w), 2924 (w), 1692 (m), 1657 (s), 1593 (w), 1493 (w), 1427 (s), 1339 (m), 1323 (w), 1267 (w), 1238 (w), 1198 (m), 1179 (m), 1163 (m), 1111 (m), 1076 (w), 1049 (m), 1020 (w), 999 (w), 968 (w), 926 (w), 916 (w), 889 (w), 866 (w), 766 (s), 716 (s), 691 (s), 675 (m), 631 (w), 606 (w). Anal. calcd. for $C_{24}H_{20}N_2O_3$ (384.4): C 74.98, H 5.24, N 7.29; Found: C 74.87, H 5.48, N 7.11.

3.8 1-(5-Benzoyl-5-hydroxy-3-phenyl-4,5-dihydro-1*H*-pyrazol-1-yl)-2-methylpropan-1-one (5h)

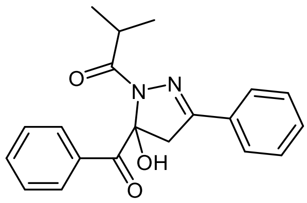

According to the GP and chromatography on silica gel (petroleum ether/ethyl acetate 6:1) compound **5h** (222 mg, 66%) was obtained as beige solid, Mp 87-89 °C; R_f (petroleum ether/ethyl acetate 6:1): 0.19. ^{1}H NMR ($CDCl_3$, 300 MHz): δ 1.05 (d, J = 7.0 Hz, 3 H), 1.17 (d, J = 6.9 Hz, 3 H), 3.42 (sept, J = 7.0 Hz, 1 H), 3.46 (d, J = 18.5 Hz, 1 H), 3.71 (d, J = 18.5 Hz, 1 H), 5.68 (br, 1 H), 7.35-7.50 (m, 5 H), 7.53-7.60 (tt, J = 7.4 Hz, J = 1.5 Hz, 1 H), 7.75-7.89 (m, 4 H). ^{13}C NMR ($CDCl_3$, 75 MHz): δ 18.1 (CH_3), 18.5 (CH_3), 32.0 (CH), 46.4 (CH_2), 90.9 (C_{quat}), 126.9 (CH), 128.9 (CH), 129.0 (CH), 129.2 (CH), 130.9 (CH), 131.1 (C_{quat}), 131.7 (C_{quat}), 134.1 (CH), 152.2 (C_{quat}), 176.0 (C_{quat}), 193.7 (C_{quat}). MS (EI), *m/z*: 318 ($(M - H_2O)^+$, 0.3), 248 ($(M - C_4H_7O - H_2O + H)^+$, 4), 231 ($(M - C_7H_5O)^+$, 39), 162 (11), 161 ($(M - C_4H_7O - C_7H_5O + H)^+$, 100), 105 ($C_7H_5O^+$, 11), 77 ($C_6H_5^+$, 11), 71 ($C_4H_7O^+$, 4). IR (ATR), $\tilde{\nu}$ [cm^{-1}]: 3402 (w), 2972 (w), 2924 (w), 2855 (w), 1738 (w), 1695 (s), 1657 (s), 1595 (w), 1468 (m), 1447 (m), 1423 (s), 1342 (m), 1281 (m), 1267 (m), 1223 (m), 1194 (s), 1180 (s), 1092 (m), 1043 (m), 1018 (m), 947 (m), 910 (m), 866 (m), 843 (m), 770 (s), 708 (s), 691 (s), 677 (s). Anal. calcd. for $C_{20}H_{20}N_2O_3$ (336.4): C 71.41, H 5.99, N 8.33; Found: C 71.61, H 6.17, N 8.09.

3.9 (5-Benzoyl-5-hydroxy-3-phenyl-4,5-dihydro-1*H*-pyrazol-1-yl)(cyclopropyl)-methanone (5i)

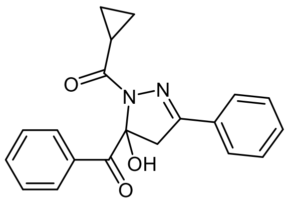

According to the GP and chromatography on silica gel (petroleum ether/ethyl acetate 6:1) compound **5i** (232 mg, 69%) was obtained as colorless solid, Mp 84 °C; R_f (petroleum ether/ethyl acetate 6:1): 0.15. ^{1}H NMR ($CDCl_3$, 300 MHz): δ 0.65-1.10 (m, 4 H), 2.55-2.70 (m, 1 H), 3.39 (d, J = 18.5 Hz, 1 H), 3.64 (d, J = 18.5 Hz, 1 H), 5.50 (br, 1 H), 7.28-7.44 (m, 5 H), 7.44-7.54 (m, 1 H), 7.67-7.76 (m, 2 H), 7.76-7.84 (m, 2 H). ^{13}C NMR ($CDCl_3$, 75 MHz): δ 8.7 (CH_2), 8.9 (CH_2), 11.9 (CH), 46.3 (CH_2), 90.7 (C_{quat}), 126.7 (CH), 128.79 (CH), 128.83 (CH), 129.0 (CH), 130.7 (CH), 130.9 (C_{quat}), 131.7 (C_{quat}), 133.9 (CH), 152.3 (C_{quat}), 172.8 (C_{quat}), 193.5 (C_{quat}). MS (EI), *m/z*: 316 ($(M - H_2O)^+$, 0.8), 248 ($(M - C_4H_5O - H_2O + H)^+$, 8), 229 ($(M - C_7H_5O)^+$, 65), 162 (11), 161 ($(M - C_4H_5O - C_7H_5O + H)^+$, 100), 105 ($C_7H_5O^+$, 11), 77 ($C_6H_5^+$, 13), 69 ($C_4H_5O^+$, 19). IR (ATR), $\tilde{\nu}$ [cm^{-1}]: 3393 (w), 1695 (m), 1645 (m), 1597 (w), 1429 (s), 1360 (m), 1285 (m), 1240 (m), 1200 (m), 1182 (m), 1103 (m), 1049 (m), 1018 (w), 953 (m), 916 (m), 887 (w), 866 (m), 787 (w), 760 (m), 733 (w), 706 (s), 691 (s), 675 (m), 604 (w). Anal. calcd. for $C_{20}H_{18}N_2O_3$ (334.4): C 71.84, H 5.43, N 8.38; Found: C 72.07, H 5.62, N 8.16.

3.10 1-(5-Benzoyl-5-hydroxy-3-phenyl-4,5-dihydro-1*H*-pyrazol-1-yl)-2,2-dimethylpropan-1-one (5j)

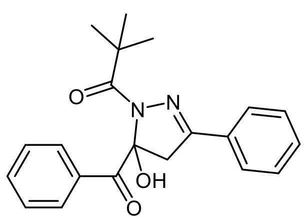

According to the GP and chromatography on silica gel (petroleum ether/ethyl acetate 4:1) compound **5j** (203 mg, 58%) was obtained as beige solid, Mp 133 °C; R_f (petroleum ether/ethyl acetate 4:1): 0.47. ^{1}H NMR ($CDCl_3$, 300 MHz): δ 1.34 (s, 9 H), 3.37 (d, J = 18.5 Hz, 1 H), 3.60 (d, J = 18.5 Hz, 1 H), 5.65 (s, 1 H), 7.35-7.60 (m, 6 H), 7.74-7.88 (m, 4 H). ^{13}C NMR ($CDCl_3$, 75 MHz): δ 26.8 (CH_3), 40.0 (CH_2), 45.1 (C_{quat}), 92.5 (C_{quat}), 127.1 (CH), 129.1 (CH), 129.3 (CH), 129.4 (CH), 131.0 (CH), 131.5 (C_{quat}), 132.1 (C_{quat}), 134.2 (CH), 151.7 (C_{quat}), 176.7 (C_{quat}), 194.0 (C_{quat}). MS (EI), *m/z*: 332 ($(M - H_2O)^+$, 0.4), 245 ($(M - C_7H_5O)^+$, 18), 238 (19), 161 (26), 105 ($C_7H_5O^+$, 100), 77 ($C_6H_5^+$, 21), 57 ($C_4H_9^+$, 14). IR (ATR), $\tilde{\nu}$ [cm^{-1}]: 2922 (w), 1732 (w), 1686 (s), 1645 (s), 1597 (w), 1485 (w), 1447 (m), 1408 (s), 1366 (m), 1323 (m), 1306 (w), 1265 (m), 1248 (m), 1223 (m), 1198 (m), 1184 (s), 1159 (m), 1130 (w), 1101 (m), 1065 (w), 1047 (m), 1032 (m), 1022 (m), 1001 (w), 947 (m), 926 (w), 899 (m), 881 (m), 866 (w), 806 (w), 787 (m), 766 (s), 746 (w), 708 (s), 692 (s), 677 (m), 638 (m). Anal. calcd. for $C_{21}H_{22}N_2O_3$ (350.4): C 71.98, H 6.33, N 7.99; Found: C 71.75, H 6.06, N 7.76.

3.11 1-(5-Benzoyl-5-hydroxy-3-phenyl-4,5-dihydro-1*H*-pyrazol-1-yl)butan-1-one (5k)

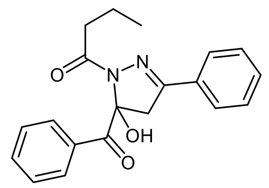

According to the GP and chromatography on silica gel (petroleum ether/ethyl acetate 7:1) compound **5k** (111 mg, 33%) was obtained as light orange solid, Mp 100 °C; R_f (petroleum ether/ethyl acetate 7:1): 0.15. ^{1}H NMR ($CDCl_3$, 300 MHz): δ 0.89 (t, *J* = 7.4 Hz, 3 H), 1.56-1.72 (m, 2 H), 2.69-2.77 (m, 2 H), 3.46 (d, *J* = 18.5 Hz, 1 H), 3.71 (d, *J* = 18.5 Hz, 1 H), 5.30-6.00 (br, 1 H), 7.36-7.50 (m, 5 H), 7.52-7.61 (m, 1 H), 7.75-7.81 (m, 2 H), 7.82-7.88 (m, 2 H). ^{13}C NMR ($CDCl_3$, 75 MHz): δ 14.1 (CH_3), 18.2 (CH_2), 36.3 (CH_2), 46.8 (CH_2), 91.1 (C_{quat}), 127.1 (CH), 129.18 (CH), 129.24 (CH), 129.4 (CH), 131.1 (CH), 131.3 (C_{quat}), 132.0 (C_{quat}), 134.3 (CH), 152.4 (C_{quat}), 172.6 (C_{quat}), 194.0 (C_{quat}). MS (EI), *m/z*: 318 ($(M - H_2O)^+$, 0.2), 248 ($(M - C_4H_7O - H_2O + H)^+$, 4), 231 ($(M - C_7H_5O)^+$, 36), 162 (11), 161 (100), 105 ($C_7H_5O^+$, 6), 77 ($C_6H_5^+$, 9). IR (ATR), $\tilde{\nu}$ [cm^{-1}]: 1686 (s), 1668 (s), 1595 (w), 1580 (w), 1447 (m), 1423 (s), 1402 (m), 1362 (w), 1329 (w), 1308 (w), 1271 (m), 1260 (m), 1236 (m), 1200 (m), 1184 (m), 1169 (w), 1099 (m), 1047 (m), 1018 (w), 1001 (w), 957 (w), 918 (w), 895 (w), 881 (s), 868 (w), 760 (s), 708 (s), 691 (s), 677 (w), 611 (w). Anal. calcd. for $C_{20}H_{20}N_2O_3$ (336.4): C 71.41, H 5.99, N 8.33; Found: C 71.42, H 5.82, N 8.28.

3.12 (5-Hydroxy-3-(4-methoxyphenyl)-4,5-dihydro-1*H*-pyrazol-1,5-diyl)bis(phenylmethanone) (5l)

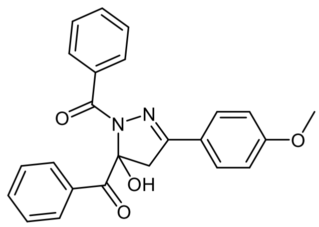

According to the GP and chromatography on silica gel (petroleum ether/ethyl acetate 5:1) compound **5l** (220 mg, 55%) was obtained as beige solid, Mp 48-50 °C; R_f (petroleum ether/ethyl acetate 5:1): 0.13. ^{1}H NMR ($CDCl_3$, 300 MHz): δ 3.50 (d, *J* = 18.4 Hz, 1 H), 3.72 (d, *J* = 18.4 Hz, 1 H), 3.86 (s, 3 H), 4.60-6.50 (br, 1 H), 6.91-7.02 (m, 2 H), 7.36-7.60 (m, 6 H), 7.66-7.76 (m, 2 H), 7.90-8.04 (m, 4 H). ^{13}C NMR ($CDCl_3$, 75 MHz): δ 46.0 (CH_2), 55.8 (CH_3), 92.5 (C_{quat}), 114.7 (CH), 123.7 (C_{quat}), 128.1 (CH), 128.9 (CH), 129.2 (CH), 129.4 (CH), 130.6 (CH), 132.0 (CH), 132.3 (C_{quat}), 133.4 (C_{quat}), 134.3 (CH), 153.3 (C_{quat}), 162.1 (C_{quat}), 166.9 (C_{quat}), 194.0 (C_{quat}). MS (EI), *m/z*: 382 ($(M - H_2O)^+$, 2), 296 (12), 295 ($(M - C_7H_5O)^+$, 65), 279 (10), 278 (51), 223 (16), 149 (17), 106 (10), 105 ($C_7H_5O^+$, 100), 77 ($C_6H_5^+$, 29), 43 (18). IR (ATR), $\tilde{\nu}$ [cm^{-1}]: 2926 (w), 1690 (w), 1636 (m), 1605 (m), 1576 (w), 1518 (w), 1493 (w), 1449 (m), 1427 (m), 1410 (m), 1331 (m), 1308 (m), 1252 (s), 1200 (m), 1177 (s), 1103 (m), 1059 (w), 1018 (m), 930 (w), 893 (w), 868 (m), 831 (m), 795 (m), 700 (s), 671 (m), 662 (w). Anal. calcd. for $C_{24}H_{20}N_2O_4$ (400.4): C 71.99, H 5.03, N 7.00; Found: C 71.88, H 5.31, N 6.71.

3.13 (3-(4-(*tert*-Butyl)phenyl)-5-hydroxy-4,5-dihydro-1*H*-pyrazol-1,5-diyl)bis(phenylmethanone) (5m)

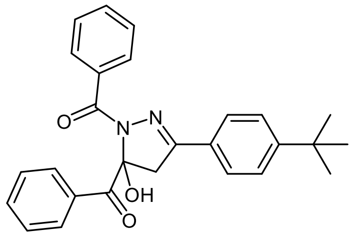

According to the GP and chromatography on silica gel (petroleum ether/ethyl acetate 7:1) compound **5m** (293 mg, 69%) was obtained as beige solid, Mp 64-68 °C; R_f (petroleum ether/ethyl acetate 7:1): 0.18. ^{1}H NMR ($CDCl_3$, 300 MHz): δ 1.35 (s, 9 H), 3.52 (d, J = 18.5 Hz, 1 H), 3.75 (d, J = 18.5 Hz, 1 H), 4.70-6.30 (br, 1 H), 7.36-7.59 (m, 8 H), 7.71 (dt, J = 8.7 Hz, J = 2.0 Hz, 2 H), 7.90-8.03 (m, 4 H). ^{13}C NMR ($CDCl_3$, 75 MHz): δ 31.5 (CH_3), 35.4 (C_{quat}), 46.0 (CH_2), 92.5 (C_{quat}), 126.2 (CH), 127.1 (CH), 128.1 (CH), 128.3 (C_{quat}), 129.2 (CH), 129.4 (CH), 130.6 (CH), 132.0 (CH), 132.2 (C_{quat}), 133.3 (C_{quat}), 134.3 (CH), 153.5 (C_{quat}), 154.9 (C_{quat}), 166.9 (C_{quat}), 193.9 (C_{quat}). MS (EI), *m/z*: 408 ((M – H_2O)$^+$, 2), 393 ((M – H_2O – CH_3)$^+$, 2), 322 (15), 321 ((M – C_7H_5O + H)$^+$, 63), 304 ((M – C_7H_5O – H_2O + H)$^+$, 13), 289 (33), 106 (11), 105 ($C_7H_5O^+$, 100), 77 ($C_6H_5^+$, 22), 43 (15). IR (ATR), $\tilde{\nu}$ [cm^{-1}]: 2961 (w), 1690 (m), 1636 (m), 1599 (w), 1576 (w), 1449 (m), 1423 (s), 1406 (m), 1362 (w), 1329 (m), 1308 (w), 1269 (m), 1246 (w), 1202 (m), 1182 (m), 1101 (m), 1057 (w), 1028 (w), 937 (w), 895 (w), 870 (m), 835 (m), 789 (w), 708 (s), 693 (s), 671 (m), 652 (w). Anal. calcd. for $C_{27}H_{26}N_2O_3$ (426.5): C 76.03, H 6.14, N 6.57; Found: C 76.13, H 6.39, N 6.32.

3.14 (3-(4-Fluorophenyl)-5-hydroxy-4,5-dihydro-1*H*-pyrazol-1,5-diyl)bis(phenylmethanone) (5n)

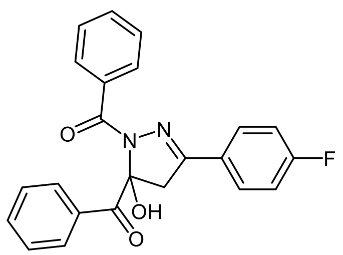

According to the GP and chromatography on silica gel (petroleum ether/ethyl acetate 5:1) compound **5n** (256 mg, 66%) was obtained as beige solid, Mp 162 °C; R_f (petroleum ether/ethyl acetate 5:1): 0.15. ^{1}H NMR ($CDCl_3$, 300 MHz): δ 3.50 (d, J = 18.4 Hz, 1 H), 3.73 (d, J = 18.4 Hz, 1 H), 5.84 (br, 1 H), 7.08-7.19 (m, 2 H), 7.37-7.48 (m, 4 H), 7.48-7.62 (m, 2 H), 7.70-7.80 (m, 2 H), 7.90-8.01 (m, 4 H). ^{13}C NMR ($CDCl_3$, 75 MHz): δ 46.0 (CH_2), 92.7 (C_{quat}), 116.5 (d, J = 22 Hz, CH), 127.4 (d, J = 3 Hz, C_{quat}), 128.2 (CH), 129.3 (CH), 129.37 (CH), 129.42 (CH), 130.5 (CH), 132.1 (CH), 132.2 (C_{quat}), 133.2 (C_{quat}), 134.4 (CH), 152.4 (C_{quat}), 164.6 (d, J = 252 Hz, C_{quat}), 167.1 (C_{quat}), 193.7 (C_{quat}). MS (EI), *m/z*: 370 ((M – H_2O)$^+$, 2), 284 (13), 283 ((M – C_7H_5O)$^+$, 73), 105 ($C_7H_5O^+$, 100), 77 ($C_6H_5^+$, 24). IR (ATR), $\tilde{\nu}$ [cm^{-1}]: 3341 (w), 1697 (m), 1626 (m), 1605 (m), 1578 (w), 1566 (w), 1516 (w), 1495 (w), 1450 (m), 1433 (m), 1410 (m), 1360 (w), 1335 (s), 1315 (w), 1302 (w), 1277 (w), 1258 (w), 1231 (w), 1204 (m), 1180 (m), 1157 (m), 1130 (w), 1115 (m), 1057 (w), 928 (w), 897 (w), 872 (m), 841 (s), 810 (w), 789 (w), 708 (s), 689 (m), 671 (m), 662 (m). Anal. calcd. for $C_{23}H_{17}FN_2O_3$ (388.4): C 71.13, H 4.41, N 7.21; Found: C 71.12, H 4.48, N 7.20.

3.15 4-(1,5-Dibenzoyl-5-hydroxy-4,5-dihydro-1*H*-pyrazol-3-yl)benzonitrile (5o)

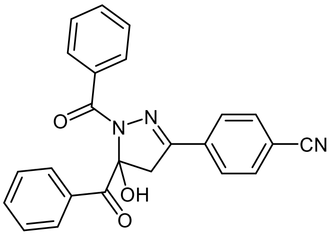

According to the GP and chromatography on silica gel (petroleum ether/ethyl acetate 4:1) compound **5o** (113 mg, 29%) was obtained as colorless solid, Mp 158 °C; R_f (petroleum ether/ethyl acetate 4:1): 0.28. ^{1}H NMR ($CDCl_3$, 300 MHz): δ 3.53 (d, J = 18.5 Hz, 1 H), 3.73 (d, J = 18.5 Hz, 1 H), 5.99 (br, 1 H), 7.38-7.49 (m, 4 H), 7.51-7.61 (m, 2 H), 7.68-7.75 (m, 2 H), 7.81-7.88 (m, 2 H), 7.89-7.99 (m, 4 H). ^{13}C NMR ($CDCl_3$, 75 MHz): δ 45.2 (CH_2), 92.7 (C_{quat}), 114.0 (C_{quat}), 118.2 (C_{quat}), 127.3 (CH), 127.9 (CH), 129.0 (CH), 129.1 (CH), 130.1 (CH), 131.8 (C_{quat}), 132.0 (CH), 132.5 (C_{quat}), 132.6 (CH), 134.2 (CH), 134.9 (C_{quat}), 151.0 (C_{quat}), 167.0 (C_{quat}), 193.0 (C_{quat}). MS (EI), *m/z*: 377 ((M – H_2O)$^+$, 1), 290 ((M – C_7H_5O)$^+$, 32), 274 (13), 273 ((M – C_7H_5O – H_2O + H)$^+$, 67), 260 (11), 259 (56), 232 (10), 231 (53), 230 (18), 216 (14), 115 (11), 105 ($C_7H_5O^+$, 100), 77 ($C_6H_5^+$, 38). IR (ATR), $\tilde{\nu}$ [cm^{-1}]: 3383 (w), 3061 (w), 2226 (w), 1668 (m), 1608 (m), 1591 (w), 1566 (m), 1450 (m), 1431 (m), 1402 (m), 1331 (m), 1317 (m), 1287 (w), 1260 (m), 1227 (m), 1188 (m), 1177 (w), 1159 (w), 1107 (m), 1069 (w), 1016 (w), 932 (w), 893 (w), 849 (m), 835 (m), 791 (w), 721 (m), 704 (s), 673 (s), 652 (m). Anal. calcd. for $C_{24}H_{17}N_3O_3$ (395.4): C 72.90, H 4.33, N 10.63; Found: C 72.99, H 4.52, N 10.90.

3.16 (1-Benzoyl-5-hydroxy-3-phenyl-4,5-dihydro-1*H*-pyrazol-5-yl)(mesityl)methanone (5p)

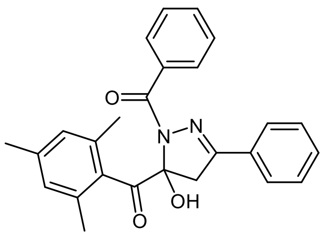

According to the GP and chromatography on silica gel (petroleum ether/ethyl acetate 15:1) compound **5p** (194 mg, 47%) was obtained as colorless solid, Mp 161 °C; R_f (petroleum ether/ethyl acetate 15:1): 0.23. ^{1}H NMR ($CDCl_3$, 300 MHz): δ 2.32 (s, 3 H), 2.35 (s, 6 H), 3.59 (d, J = 17.7 Hz, 1 H), 3.86 (d, J = 17.7 Hz, 1 H), 5.44 (br, 1 H), 6.91 (s, 2 H), 7.37-7.60 (m, 6 H), 7.68-7.78 (m, 2 H), 8.00-8.10 (m, 2 H). ^{13}C NMR ($CDCl_3$, 75 MHz): δ 20.9 (CH_3), 21.5 (CH_3), 46.8 (CH_2), 96.2 (C_{quat}), 127.2 (CH), 128.2 (CH), 129.1 (CH), 129.3 (CH), 130.7 (CH), 131.0 (CH), 131.1 (C_{quat}), 132.2 (CH), 133.2 (C_{quat}), 134.4 (C_{quat}), 136.9 (C_{quat}), 140.3 (C_{quat}), 153.7 (C_{quat}), 168.3 (C_{quat}), 208.1 (C_{quat}). MS (EI), *m/z*: 394 ((M – H_2O)$^+$, 4), 266 (17), 265 ((M – $C_{10}H_{11}O$)$^+$, 91), 147 ($C_{10}H_{11}O^+$, 17), 105 ($C_7H_5O^+$, 100), 77 ($C_6H_5^+$, 18). IR (ATR), $\tilde{\nu}$ [cm^{-1}]: 3319 (w), 1688 (m), 1616 (m), 1572 (m), 1437 (s), 1341 (s), 1315 (w), 1292 (w), 1254 (m), 1217 (w), 1202 (w), 1161 (w), 1121 (m), 1055 (w), 1016 (w), 889 (m), 848 (m), 806 (m), 795 (m), 758 (m), 727 (m), 710 (s), 685 (s), 677 (m), 638 (m), 629 (m). Anal. calcd. for $C_{26}H_{24}N_2O_3$ (412.5): C 75.71, H 5.86, N 6.79; Found: C 75.69, H 5.88, N 6.79.

3.17 (1-Benzoyl-5-hydroxy-3-phenyl-4,5-dihydro-1*H*-pyrazol-5-yl)(thien-2-yl)methanone (5q)

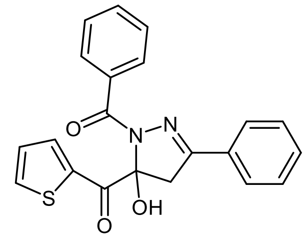

According to the GP and chromatography on silica gel (petroleum ether/ethyl acetate 5:1) compound **5q** (275 mg, 73%) was obtained as beige solid, Mp 171 °C; R_f (petroleum ether/ethyl acetate 5:1): 0.18. ^{1}H NMR ($CDCl_3$, 300 MHz): δ 3.55 (d, *J* = 18.5 Hz, 1 H), 3.73 (d, *J* = 18.5 Hz, 1 H), 5.66 (br, 1 H), 7.08 (dd, *J* = 4.9 Hz, *J* = 3.9 Hz, 1 H), 7.38-7.58 (m, 6 H), 7.67 (dd, *J* = 4.9 Hz, *J* = 1.1 Hz, 1 H), 7.71-7.79 (m, 3 H), 8.00-8.01 (m, 2 H). ^{13}C NMR ($CDCl_3$, 75 MHz): δ 46.3 (CH_2), 92.8 (C_{quat}), 127.2 (CH), 128.2 (CH), 128.8 (CH), 129.2 (CH), 130.7 (CH), 131.0 (C_{quat}), 131.3 (CH), 132.2 (CH), 133.2 (C_{quat}), 134.0 (CH), 135.6 (CH), 137.6 (C_{quat}), 153.5 (C_{quat}), 167.3 (C_{quat}), 187.4 (C_{quat}). MS (EI), *m/z*: 358 ((M – $H_2O)^+$, 1), 266 (11), 265 ((M – $C_7H_5O)^+$, 61), 185 (10), 111 (10), 105 ($C_7H_5O^+$, 100), 77 ($C_6H_5^+$, 24). IR (ATR), $\tilde{\nu}$ [cm^{-1}]: 1676 (m), 1626 (m), 1609 (m), 1566 (m), 1450 (m), 1429 (s), 1410 (s), 1339 (s), 1229 (m), 1206 (s), 1177 (m), 1111 (m), 1059 (m), 883 (m), 866 (m), 843 (w), 826 (m), 762 (m), 708 (s), 689 (s), 671 (m), 629 (m). Anal. calcd. for $C_{21}H_{16}N_2O_3S$ (376.4): C 67.00, H 4.28, N 7.44; Found: C 66.94, H 4.50, N 7.18.

3.18 (5-Hydroxy-3-(4-methoxyphenyl)-1-(thiophen-2-carbonyl)-4,5-dihydro-1*H*-pyrazol-5-yl) (mesityl)methanone (5r)

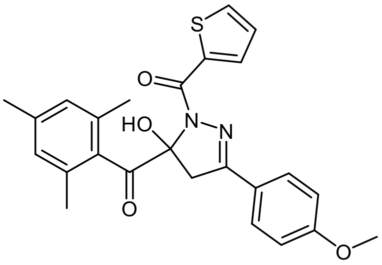

According to the GP and chromatography on silica gel (petroleum ether/ethyl acetate 10:1 to 5:1) compound **5r** (172 mg, 38%) was obtained as colorless solid, Mp 201-204 °C; R_f (petroleum ether/ethyl acetate 5:1): 0.24. ^{1}H NMR ($CDCl_3$, 300 MHz): δ 2.30 (s, 3 H), 2.33 (s, 6 H), 3.56 (d, *J* = 17.6 Hz, 1 H), 3.75-3.95 (m, 4 H), 5.34 (s, 1 H), 6.90 (br, 2 H), 6.98 (d, *J* = 8.7 Hz, 2 H), 7.10-7.19 (m, 1 H), 7.67 (d, *J* = 4.4 Hz, 1 H), 7.72-7.84 (m, 2 H), 8.18 (d, *J* = 2.9 Hz, 1 H). ^{13}C NMR ($CDCl_3$, 75 MHz): δ 20.5 (CH_3), 21.1 (CH_3), 46.4 (CH_2), 55.4 (CH_3), 95.4 (C_{quat}), 114.3 (CH), 123.3 (C_{quat}), 126.9 (CH), 128.7 (CH), 128.9 (CH), 133.8 (C_{quat}), 134.0 (C_{quat}), 134.2 (CH), 135.3 (CH), 136.5 (C_{quat}), 139.8 (C_{quat}), 153.1 (C_{quat}), 160.1 (C_{quat}), 161.7 (C_{quat}), 207.5 (C_{quat}). MS (EI), *m/z*: 430 ((M – $H_2O)^+$, 9), 319 ((M – C_5H_3OS – $H_2O)^+$, 20), 302 (18), 301 ((M – $C_{10}H_{11}O)^+$, 76), 291 (16), 147 ($C_{10}H_{11}O^+$, 17), 111 ($C_5H_3OS^+$, 100), 43 (16). IR (ATR), $\tilde{\nu}$ [cm^{-1}]: 3237 (w), 1686 (w), 1605 (s), 1591 (m), 1562 (w), 1516 (m), 1452 (s), 1420 (m), 1346 (m), 1329 (m), 1310 (m), 1252 (s), 1217 (m), 1207 (w), 1177 (m), 1161 (w), 1109 (s), 1063 (w), 1045 (m), 1038 (m), 1016 (m), 1005 (w), 941 (w), 908 (w), 881 (m), 841 (s), 816 (s), 791 (m), 733 (m), 712 (s), 681 (m), 633 (m). Anal. calcd. for $C_{25}H_{24}N_2O_4S$ (448.5): C 66.94, H 5.39, N 6.25, S 7.15; Found: C 67.19, H 5.60, N 6.02, S 7.11.

4 Attempted aromatization of 1,5-diacyl-5-hydroxypyrazoline 5b

A series of experiments was conducted to achieve a dehydrative aromatization of compound **5b** to give 1,5-diacylpyrazole **7**. In a Schlenk flask with magentic stirring bar and screw cap were placed 5-hydroxy-3-phenyl-4,5-dihydro-1*H*-pyrazol-1,5-diyl)bis(phenylmethanone) (**5b**, 37 mg, 0.1 mmol) and 1,4-dioxane (0.5 mL) under argon. Then, various additives were added to the reaction mixture and conditions were applied (for experimental details see Table S5). For workup deionized water (5 mL) was added and the mixture extracted with dichloromethane (4 × 5 mL). The combined organic phases were dried (anhydrous sodium sulfate) and the solvents were removed in vacuo. The residue was adsorbed on celite® and chromatographed on silica gel (petroleum ether/ethyl acetate 5:1) to give either **5b** or pyrazole **6a**. The spectroscopic data were in full agreement with the reference compounds.

Table S5: Attempted dehydrative aromatization of 5-hydroxypyrazoline **5b**.

Entry	Additive	Conditions	Result
1	H_2O (3.6 mg, 0.2 mmol)	rt, then 150 °C (oil bath), 47.5 h	no conversion[a]
2	CH_3CO_2H (0.1 mL, 0.2 mmol)	rt, then 150 °C (oil bath), 47.5 h	**6a** (n.i.)[b]
3	PTSA · H_2O (39 mg, 0.2 mmol)	rt, then 150 °C (oil bath), 47.5 h	**6a** (n.i.)[a]
4[d]	1 N HCl (0.25 mL, 0.25 mmol)	rt for 23 h, then 80 °C (oil bath), 7.5 h	**6a** (n.i.)[c]
5[e]	silica gel	110 °C, 18 h	no conversion[a]
6	K_2CO_3 (28 mg, 0.2 mmol)	1,4-dioxane, rt–150 °C, 47.5 h	mixture of products[a]
7[f]	pyridine (16 mg, 0.2 mmol)	CH_2Cl_2, 30 °C, 14 h	no conversion[a]

[a] TLC monitoring. [b] GC–MS monitoring. [c] Confirmed by MALDI-MS. [d] On a 0.25 mmol scale. [e] In toluene instead of 1,4-dioxane. [e] In dichloromethane instead of 1,4-dioxane.

Compound **5b** is either stable against the additives and conditions or dehydrative deacylation furnishes pyrazole **6a**.

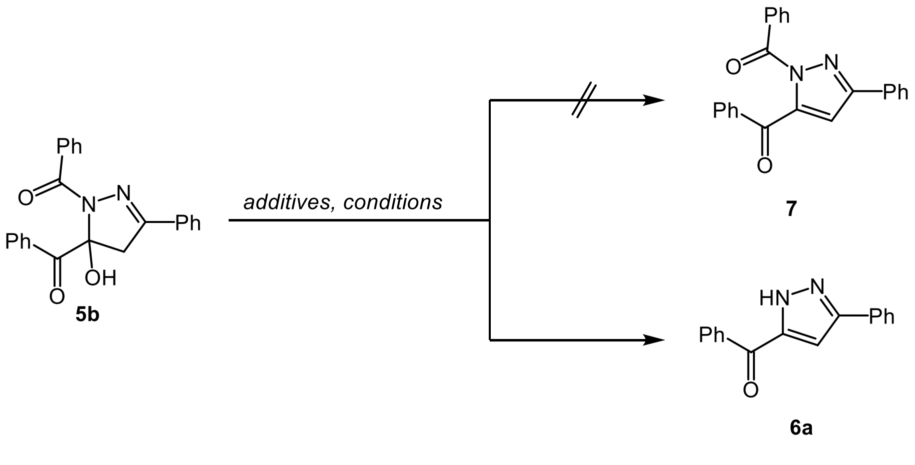

5 ^{1}H and ^{13}C NMR spectra

5.1 *tert*-Butyl 5-benzoyl-5-hydroxy-3-phenyl-4,5-dihydro-1*H*-pyrazole-1-carboxylate (5a)

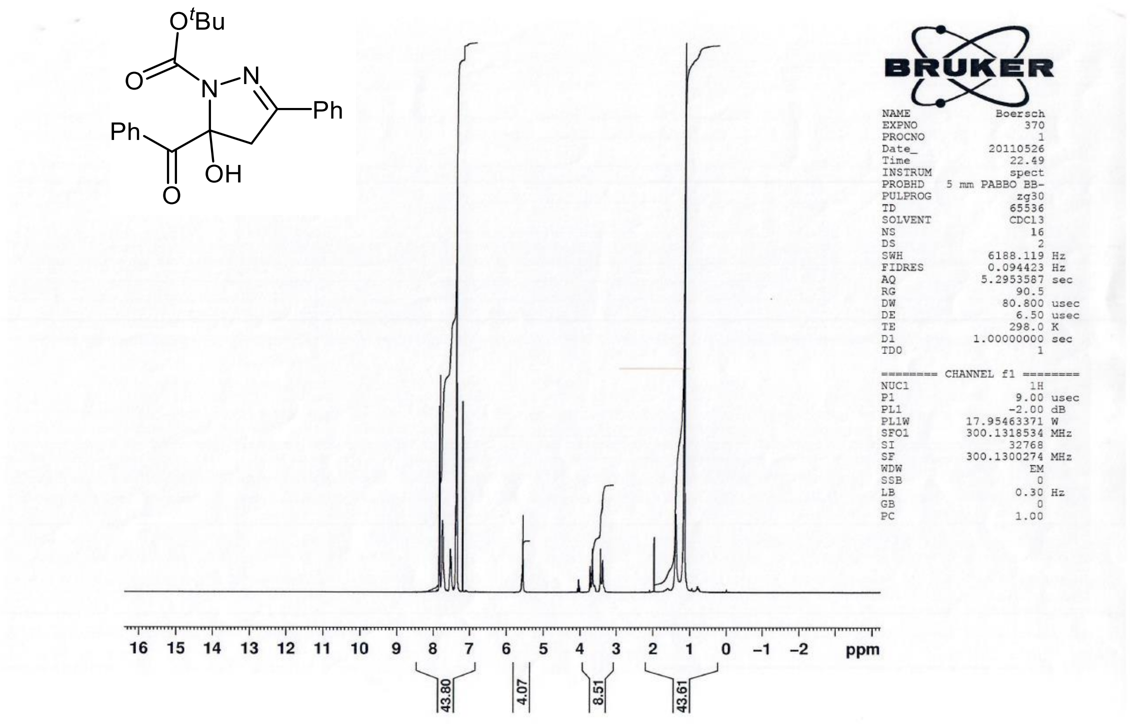

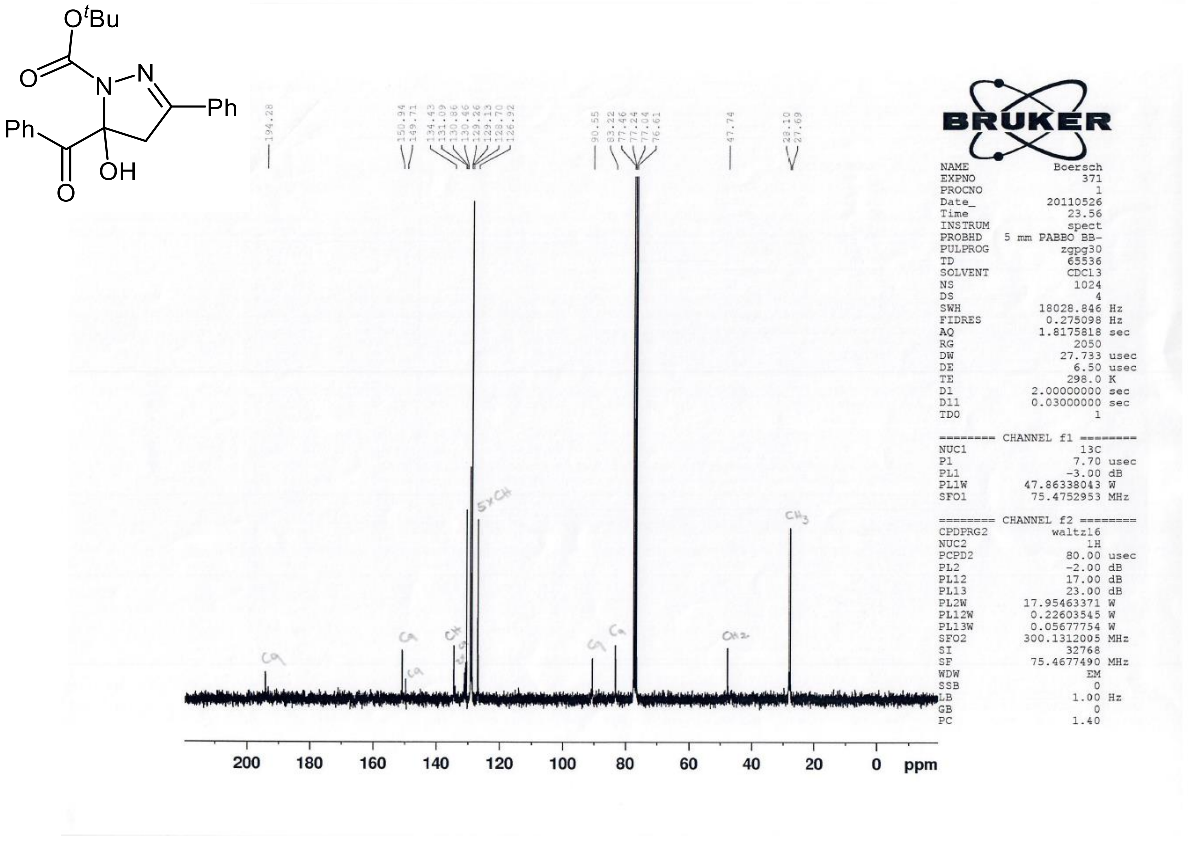

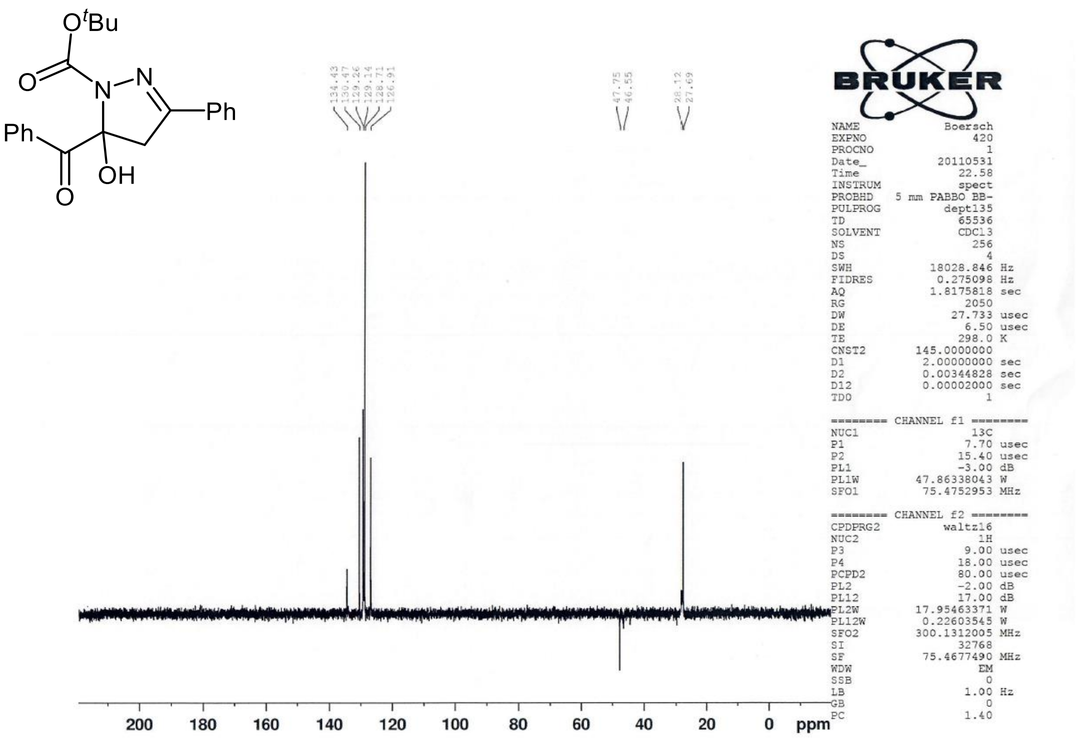

5.2 (5-Hydroxy-3-phenyl-4,5-dihydro-1*H*-pyrazol-1,5-diyl)bis(phenylmethanone) (5b)

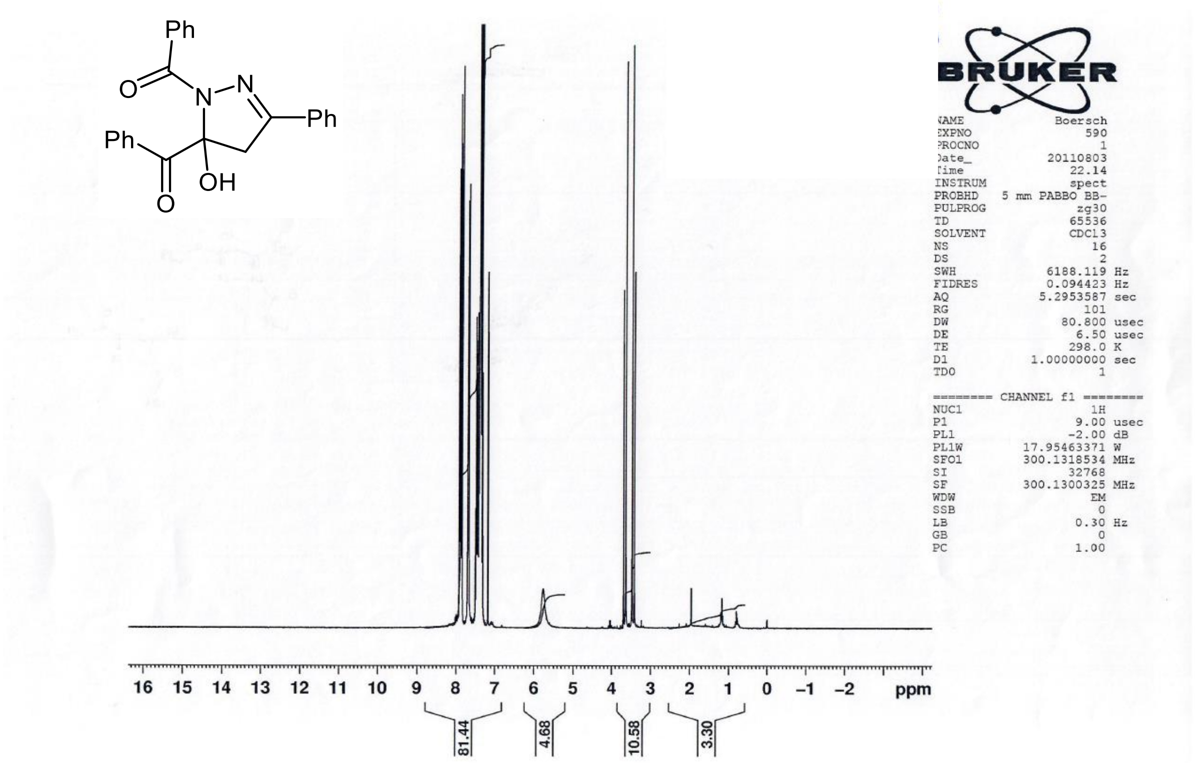

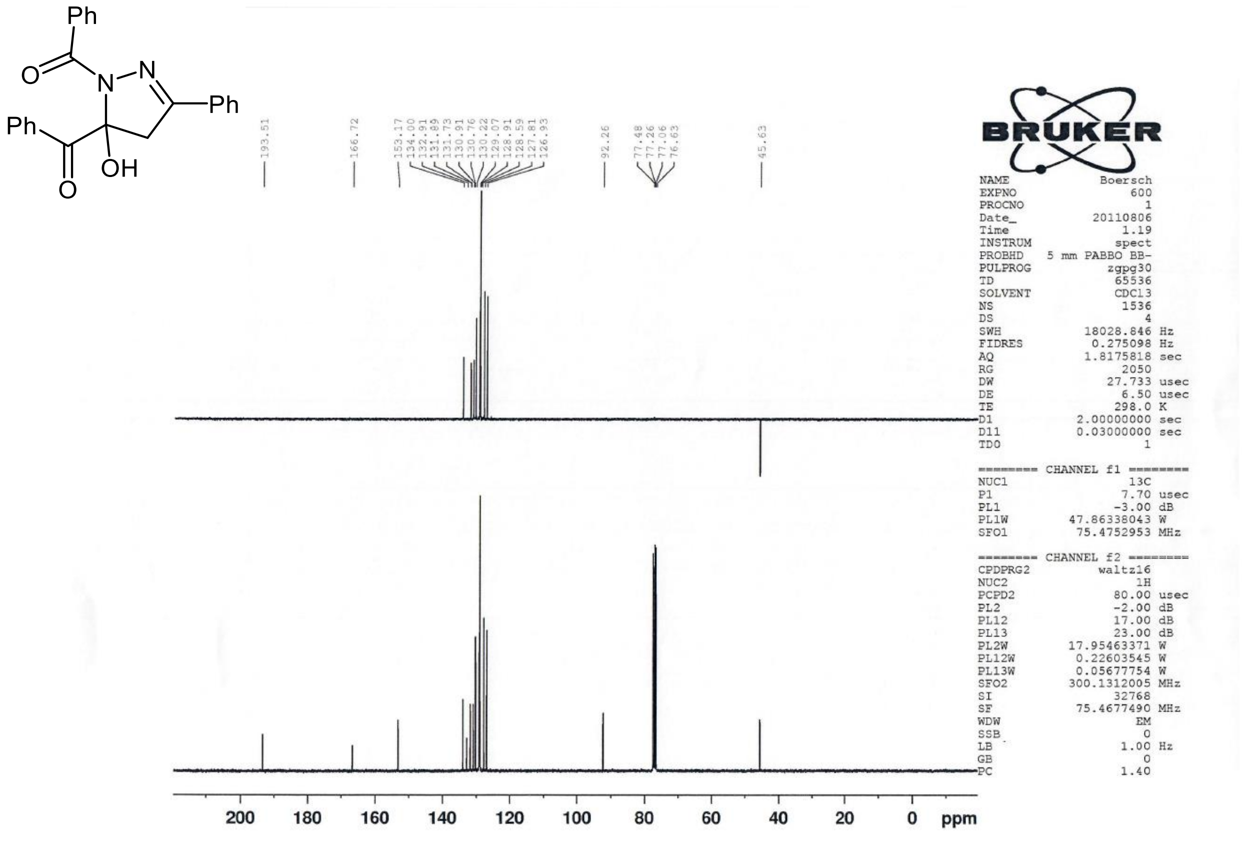

5.3 (5-Benzoyl-5-hydroxy-3-phenyl-4,5-dihydro-1*H*-pyrazol-1-yl)(4-tolyl)methanone (5c)

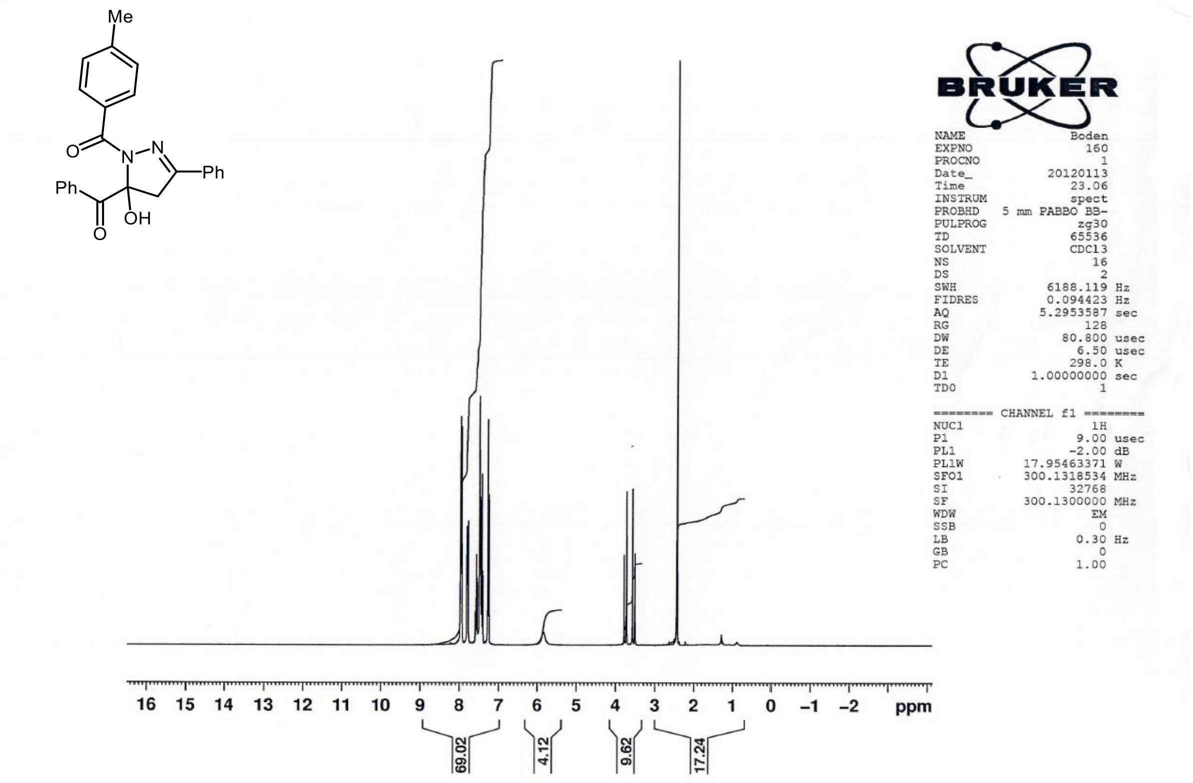

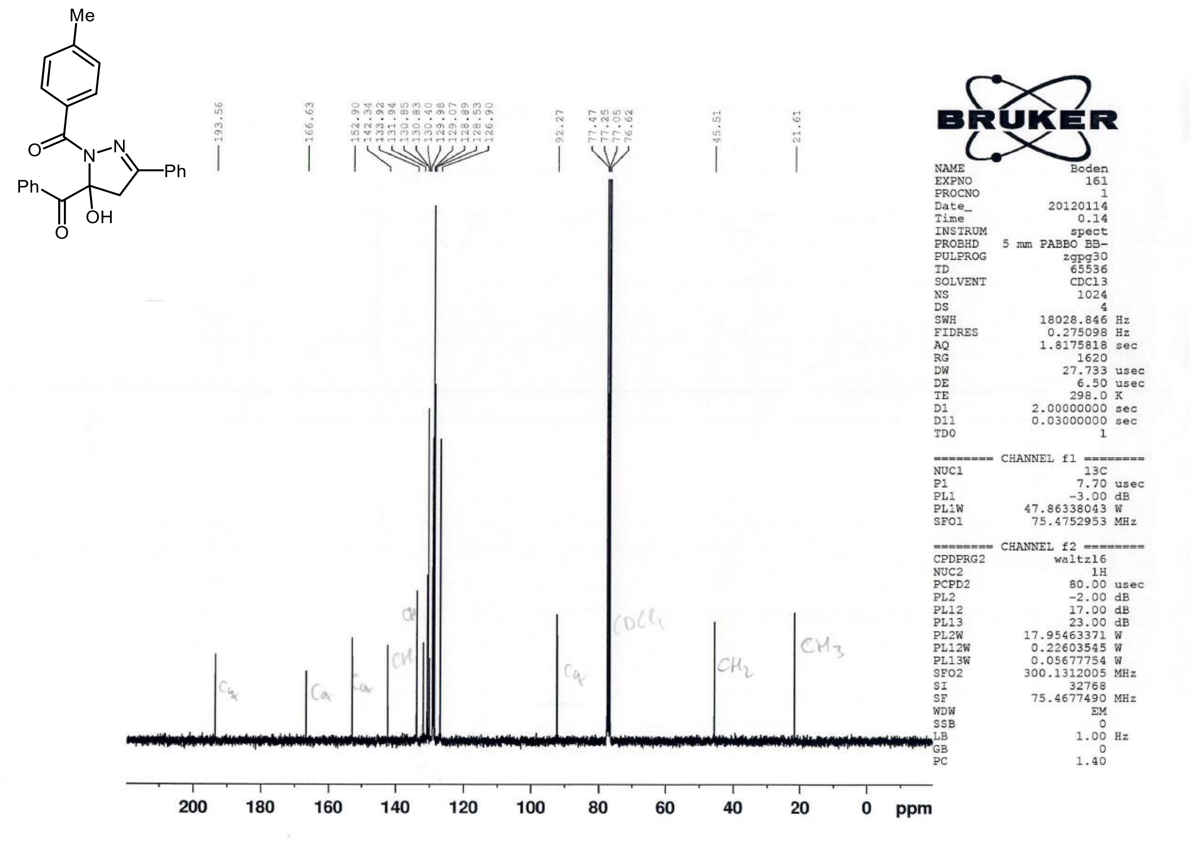

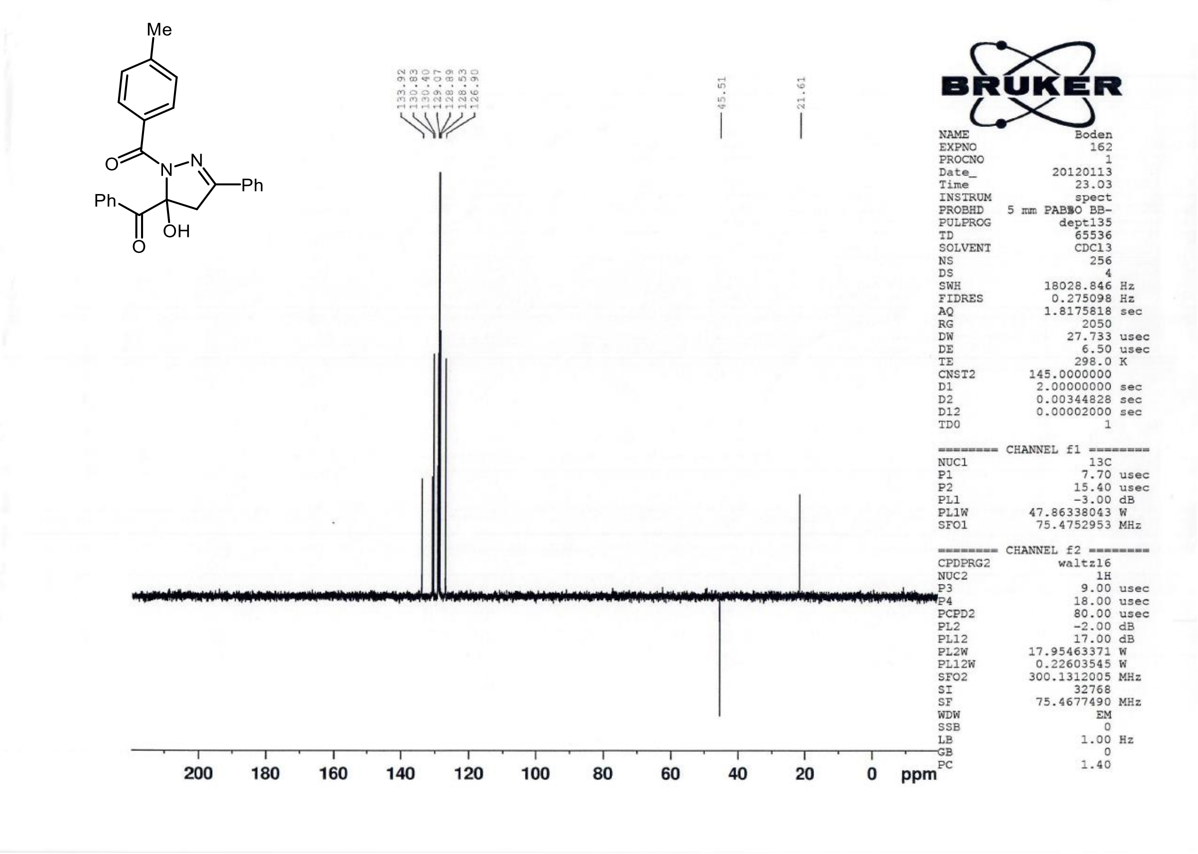
Me
O
N
N
Ph
Ph
O
OH
133.92
130.83
130.40
129.07
128.89
128.53
126.90
45.51
21.61
BRUKER
NAME Boden
EXPNO 162
PROCNO 1
Date_ 20120113
Time 23.03
INSTRUM spect
PROBHD 5 mm PABBO BB-
PULPROG dept135
TD 65536
SOLVENT CDCl3
NS 256
DS 4
SWH 18028.846 Hz
FIDRES 0.275098 Hz
AQ 1.8175818 sec
RG 2050
DW 27.733 usec
DE 6.50 usec
TE 298.0 K
CNST2 145.0000000
D1 2.00000000 sec
D2 0.00344828 sec
D12 0.00002000 sec
TD0 1
======== CHANNEL f1 ========
NUC1 13C
P1 7.70 usec
P2 15.40 usec
PL1 -3.00 dB
PL1W 47.86338043 W
SFO1 75.4752953 MHz
======== CHANNEL f2 ========
CPDPRG2 waltz16
NUC2 1H
P3 9.00 usec
P4 18.00 usec
PCPD2 80.00 usec
PL2 -2.00 dB
PL12 17.00 dB
PL2W 17.95463371 W
PL12W 0.22603545 W
SFO2 300.1312005 MHz
SI 32768
SF 75.4677490 MHz
WDW EM
SSB 0
LB 1.00 Hz
GB 0
PC 1.40
200 180 160 140 120 100 80 60 40 20 0 ppm

5.4 (5-Benzoyl-5-hydroxy-3-phenyl-4,5-dihydro-1*H*-pyrazol-1-yl)(4-bromophenyl)methanone (5d)

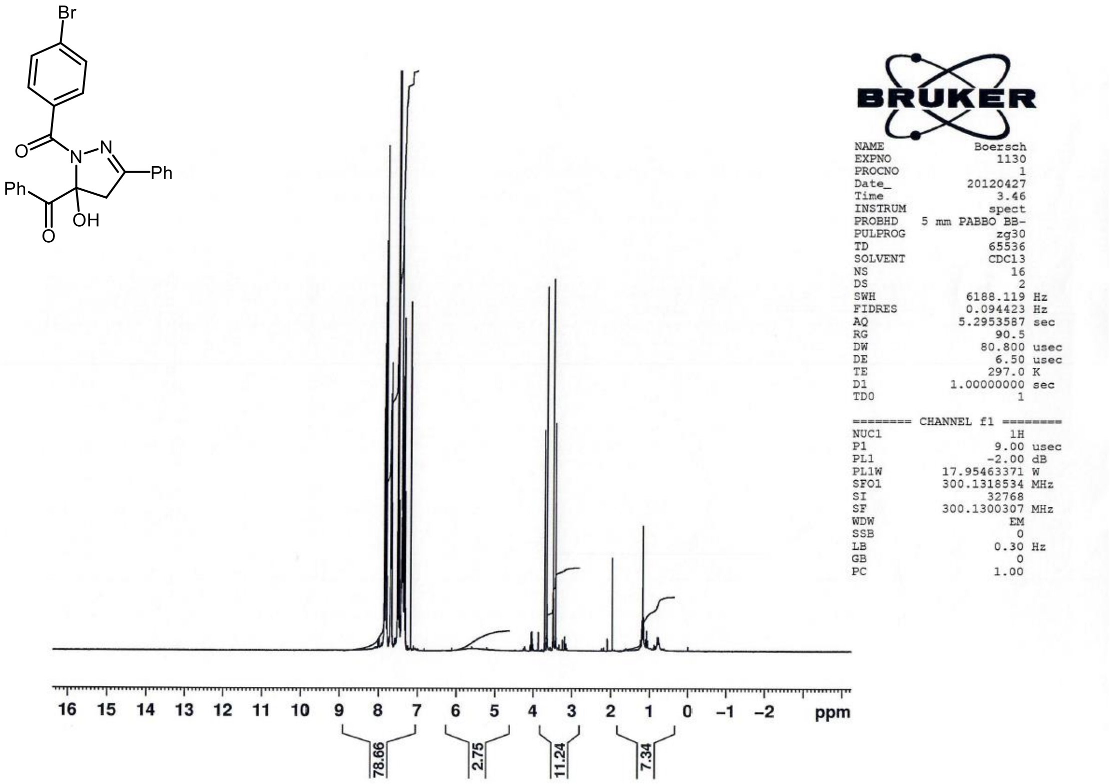

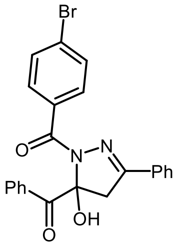

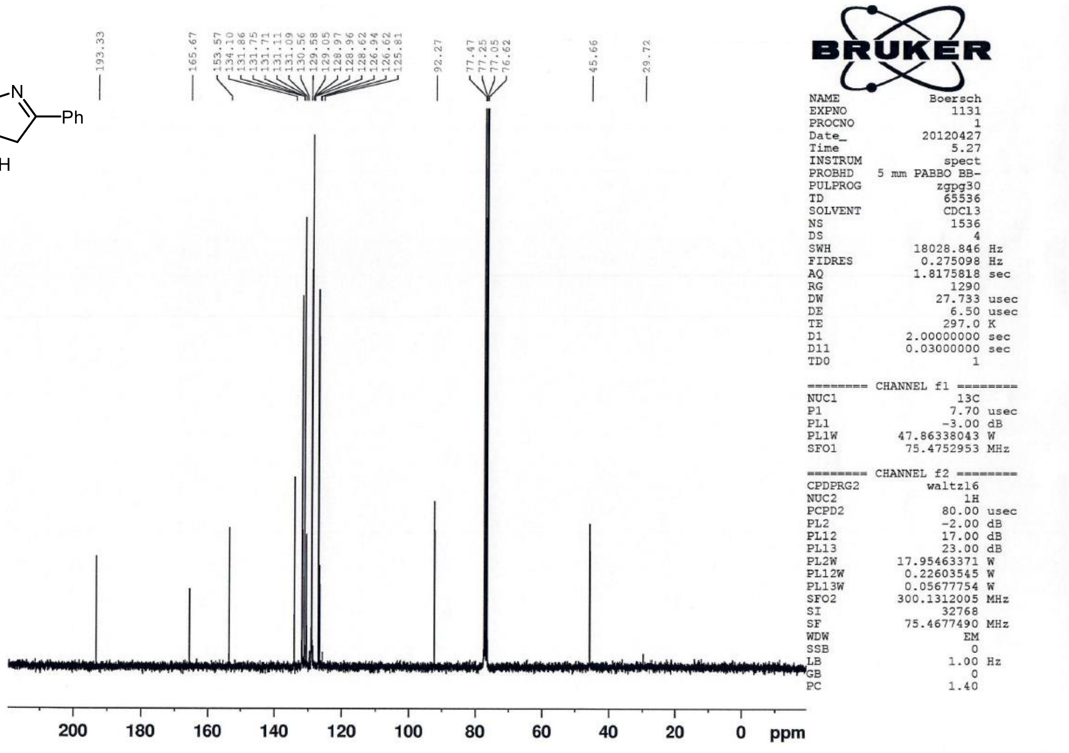

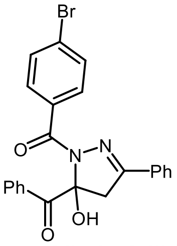
Br
O
N
N
Ph
Ph
O
OH

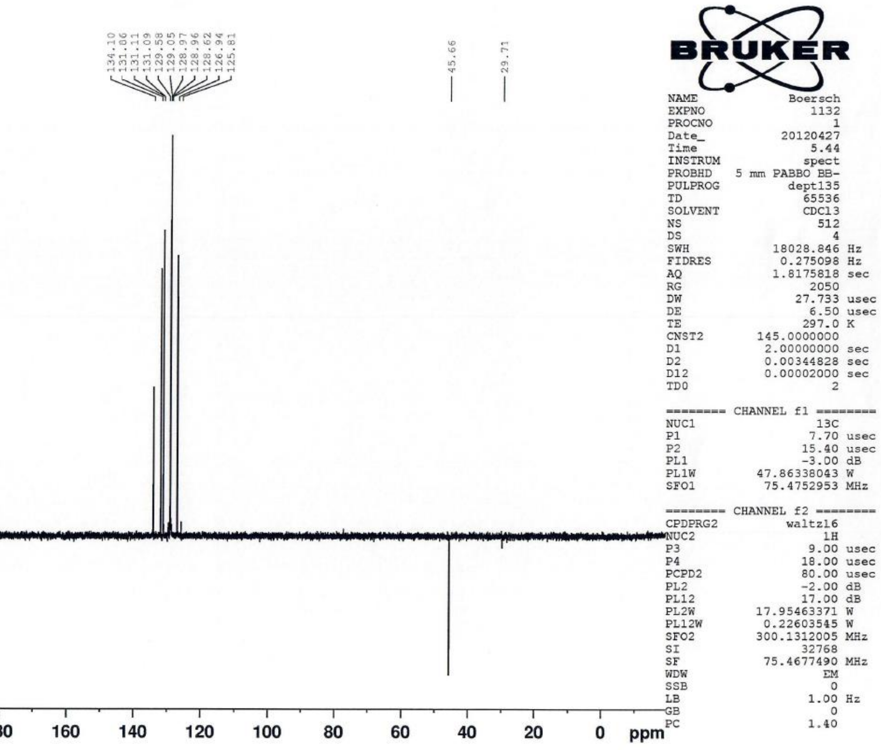
134.10
131.86
131.11
131.09
129.58
129.05
128.97
128.96
128.62
126.94
125.81
45.66
29.71
BRUKER
NAME Boersch
EXPNO 1132
PROCNO 1
Date_ 20120427
Time 5.44
INSTRUM spect
PROBHD 5 mm PABBO BB-
PULPROG dept135
TD 65536
SOLVENT CDCl3
NS 512
DS 4
SWH 18028.846 Hz
FIDRES 0.275098 Hz
AQ 1.8175818 sec
RG 2050
DW 27.733 usec
DE 6.50 usec
TE 297.0 K
CNST2 145.0000000
D1 2.00000000 sec
D2 0.00344828 sec
D12 0.00002000 sec
TD0 2
======== CHANNEL f1 ========
NUC1 13C
P1 7.70 usec
P2 15.40 usec
PL1 -3.00 dB
PL1W 47.86338043 W
SFO1 75.4752953 MHz
======== CHANNEL f2 ========
CPDPRG2 waltz16
NUC2 1H
P3 9.00 usec
P4 18.00 usec
PCPD2 80.00 usec
PL2 -2.00 dB
PL12 17.00 dB
PL2W 17.95463371 W
PL12W 0.22603545 W
SFO2 300.1312005 MHz
SI 32768
SF 75.4677490 MHz
WDW EM
SSB 0
LB 1.00 Hz
GB 0
PC 1.40
200 180 160 140 120 100 80 60 40 20 0 ppm

5.5 (5-Benzoyl-5-hydroxy-3-phenyl-4,5-dihydro-1*H*-pyrazol-1-yl)(thien-2-yl)methanone (5e)

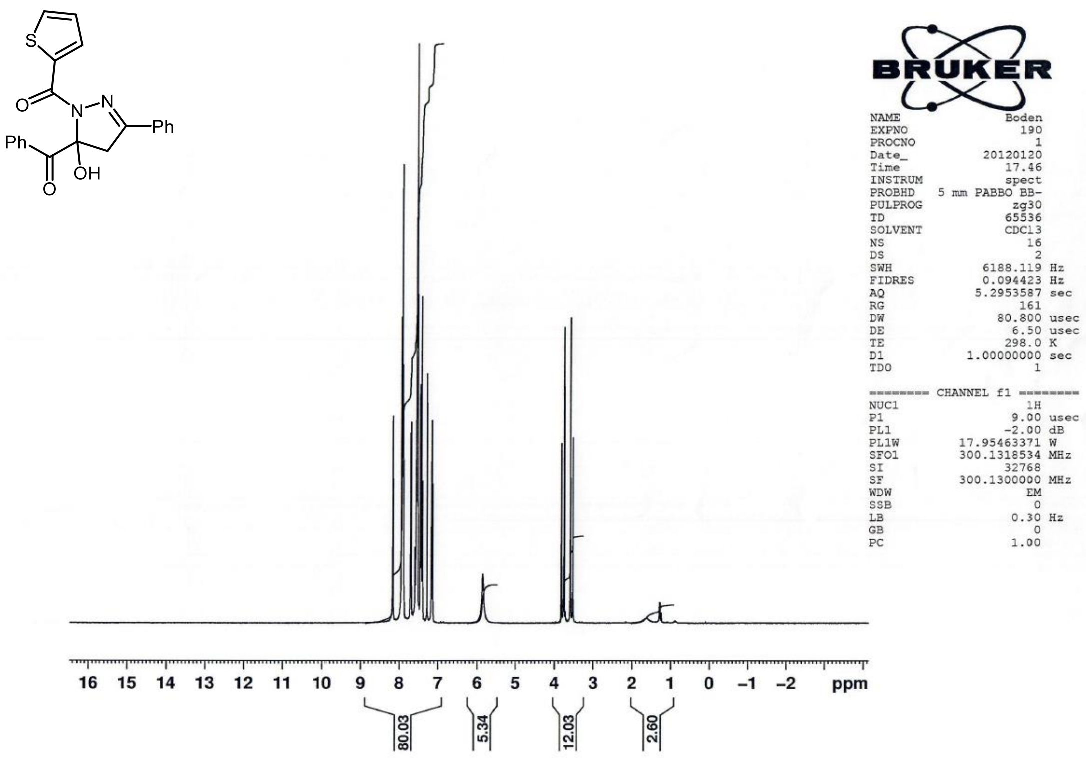

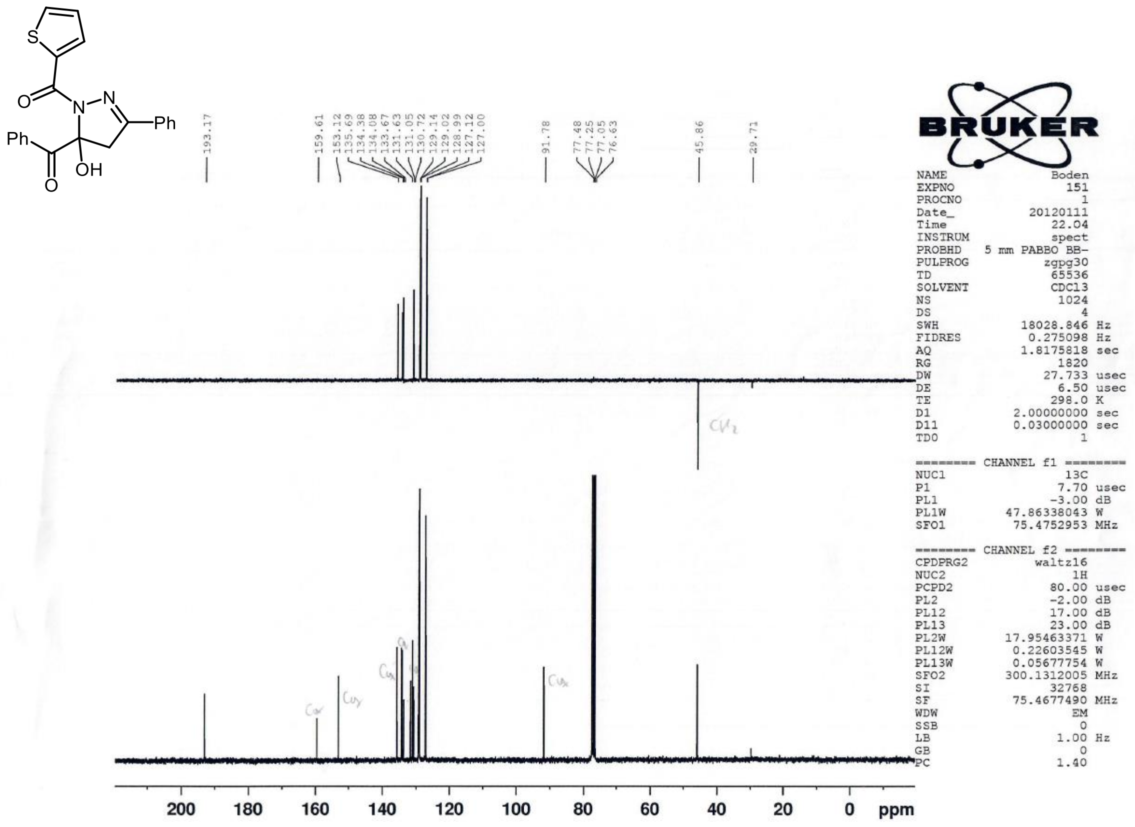

5.6 (5-Benzoyl-5-hydroxy-3-phenyl-4,5-dihydro-1*H*-pyrazol-1-yl)(furan-2-yl)methanone (5f)

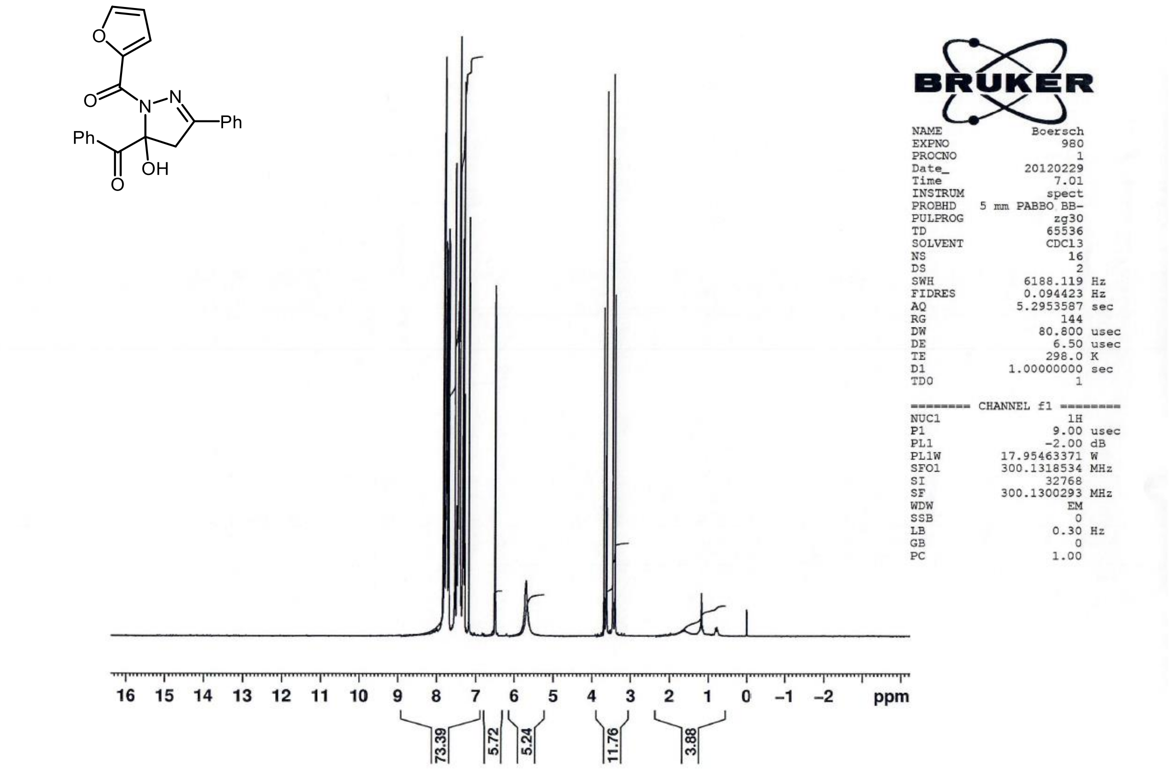

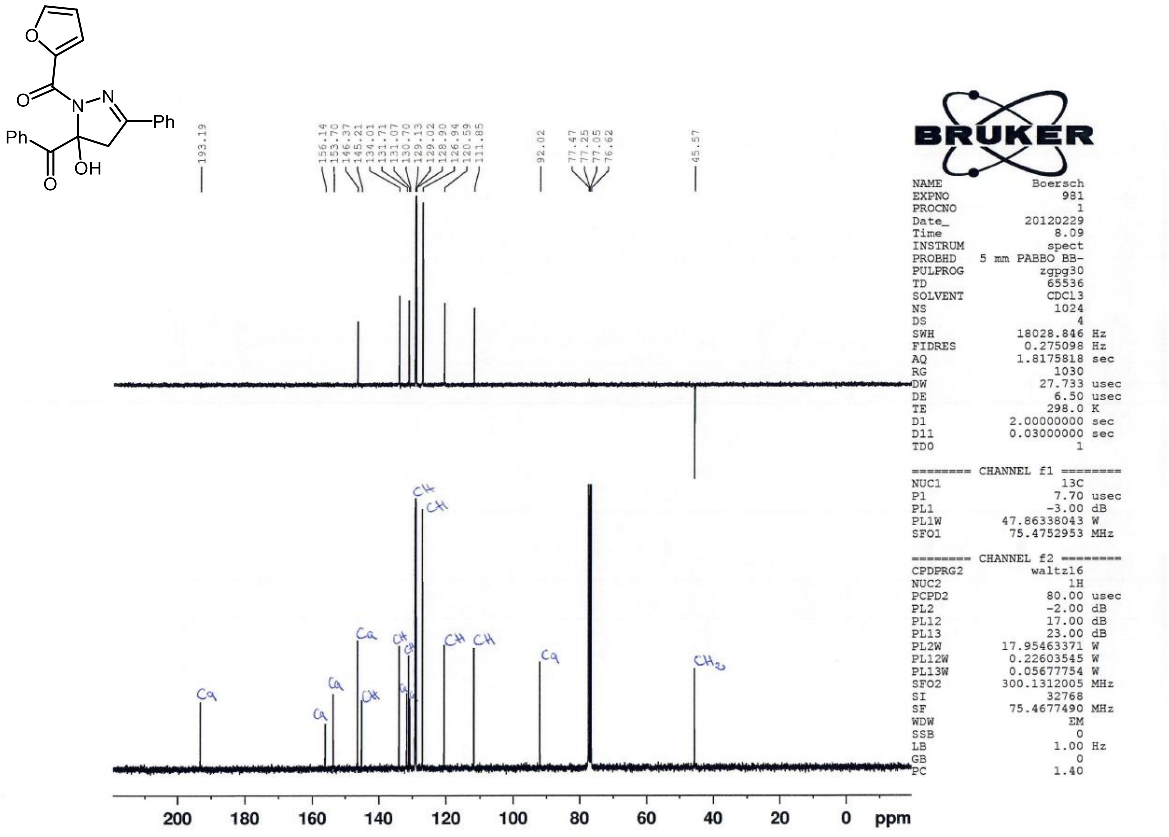

5.7 1-(5-Benzoyl-5-hydroxy-3-phenyl-4,5-dihydro-1*H*-pyrazol-1-yl)-2-phenylethan-1-one (5g)

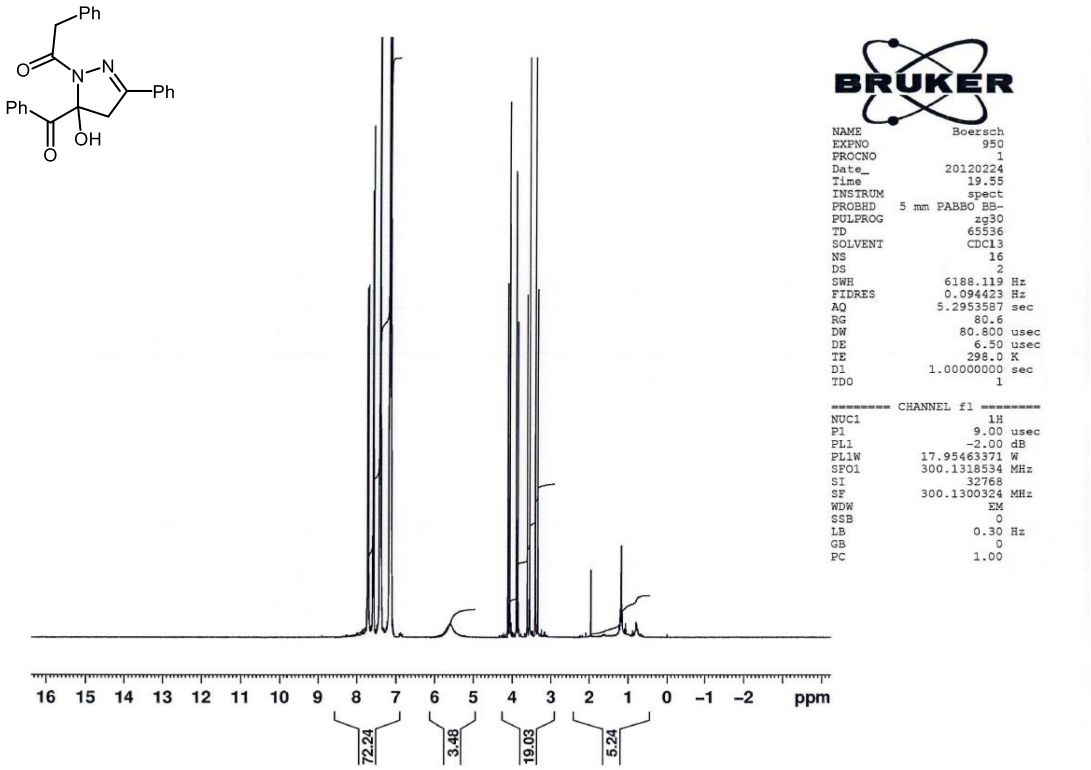

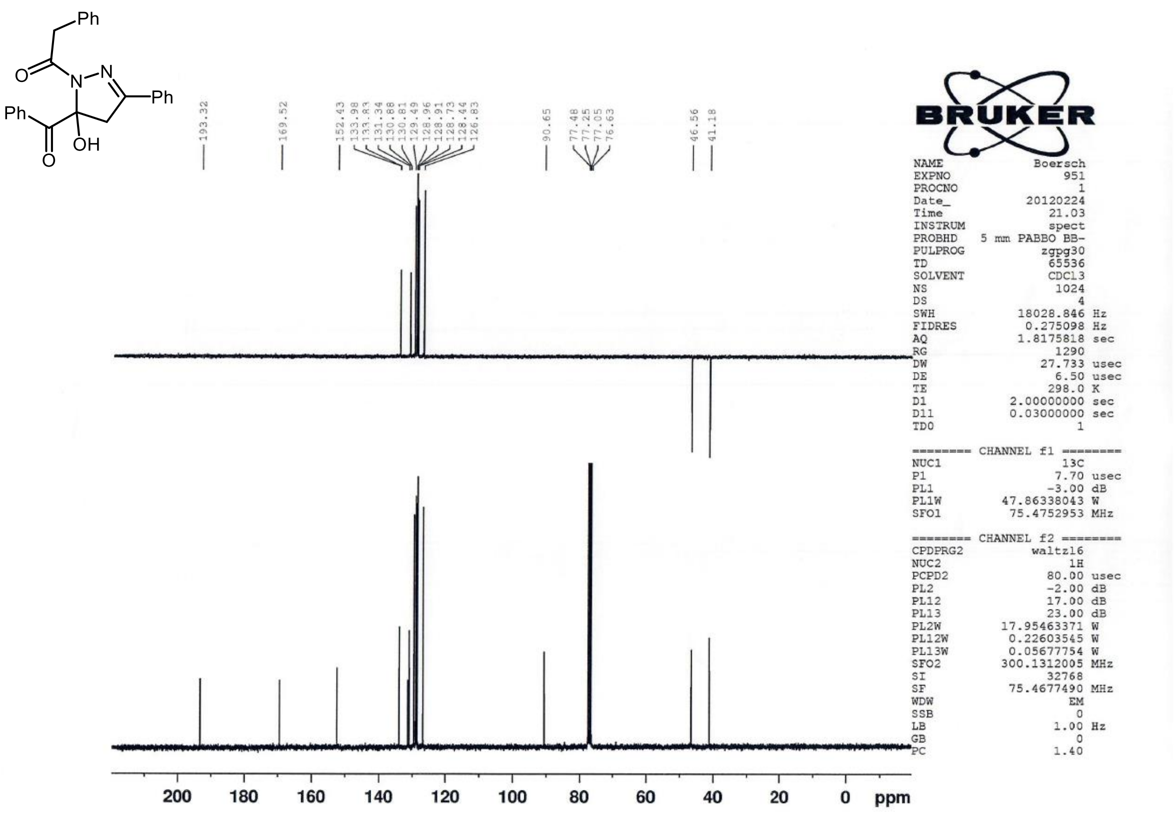

5.8 1-(5-Benzoyl-5-hydroxy-3-phenyl-4,5-dihydro-1*H*-pyrazol-1-yl)-2-methylpropan-1-one (5h)

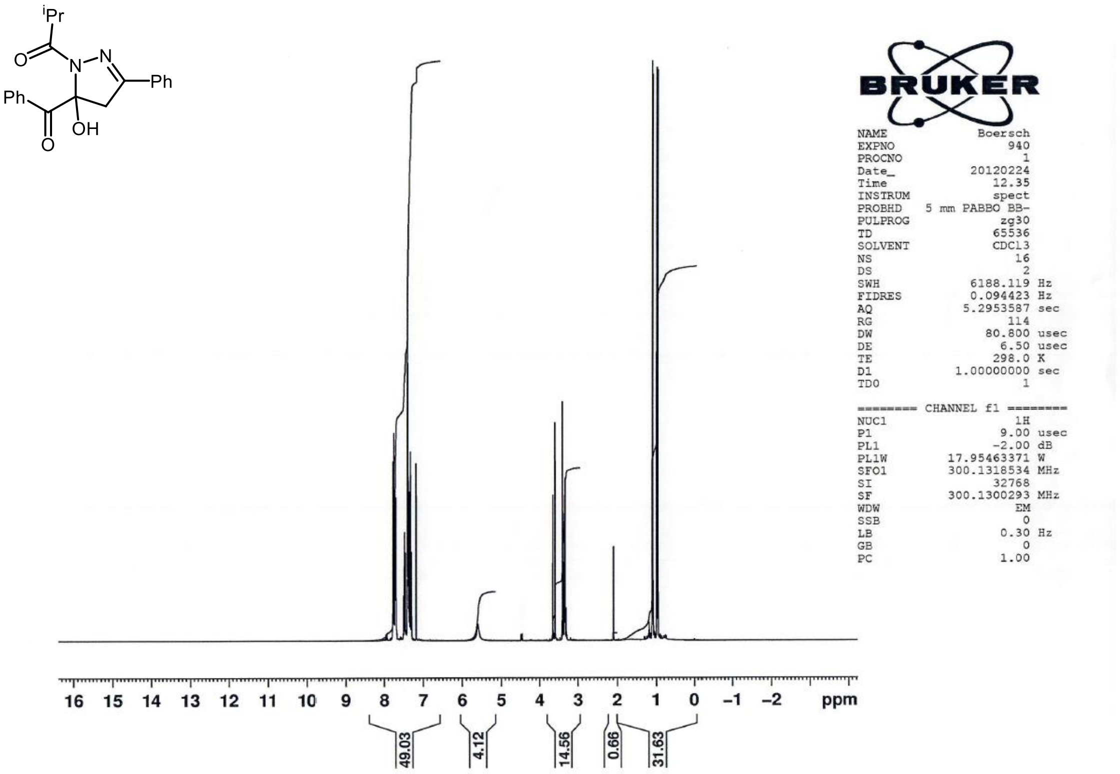

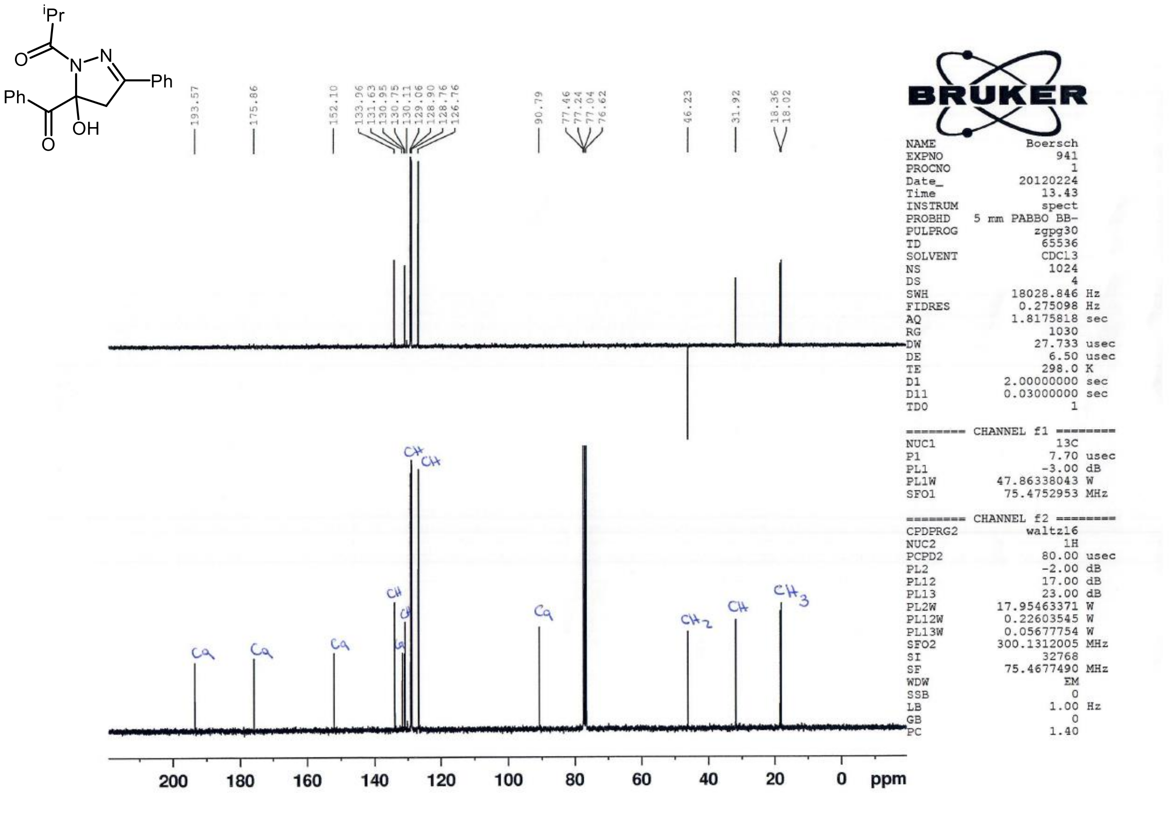

5.9 (5-Benzoyl-5-hydroxy-3-phenyl-4,5-dihydro-1*H*-pyrazol-1-yl)(cyclopropyl)-methanone (5i)

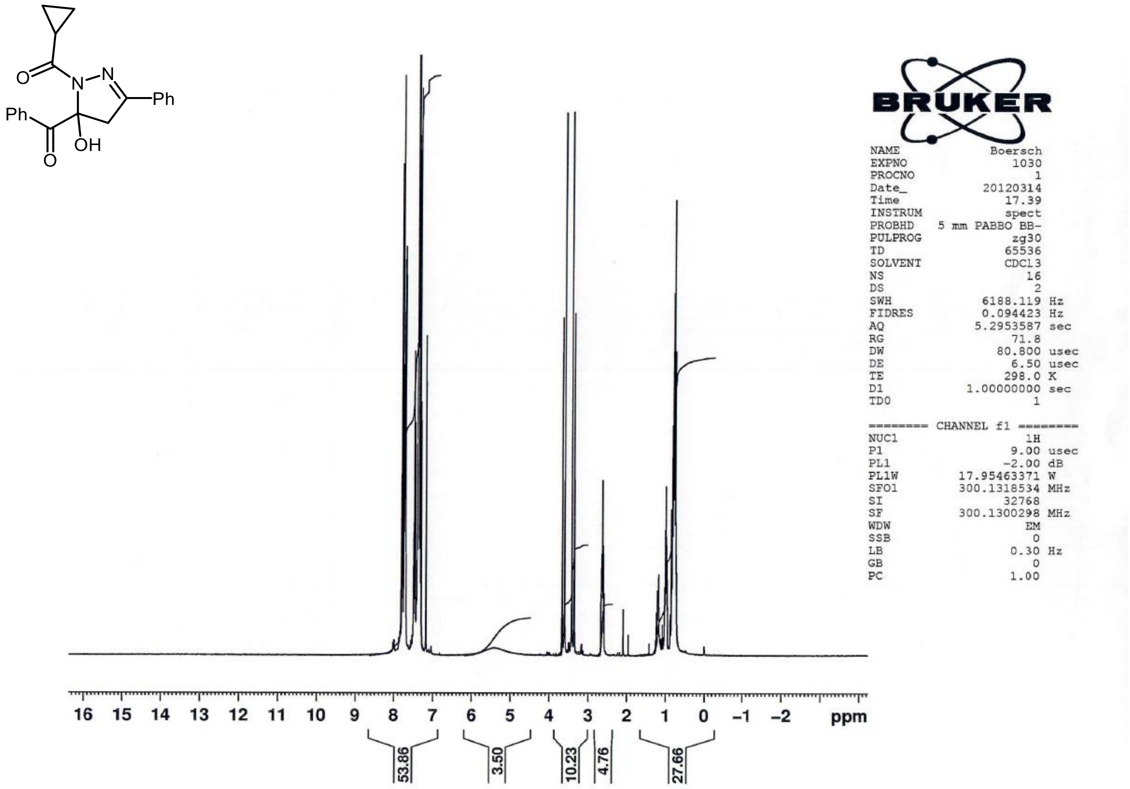

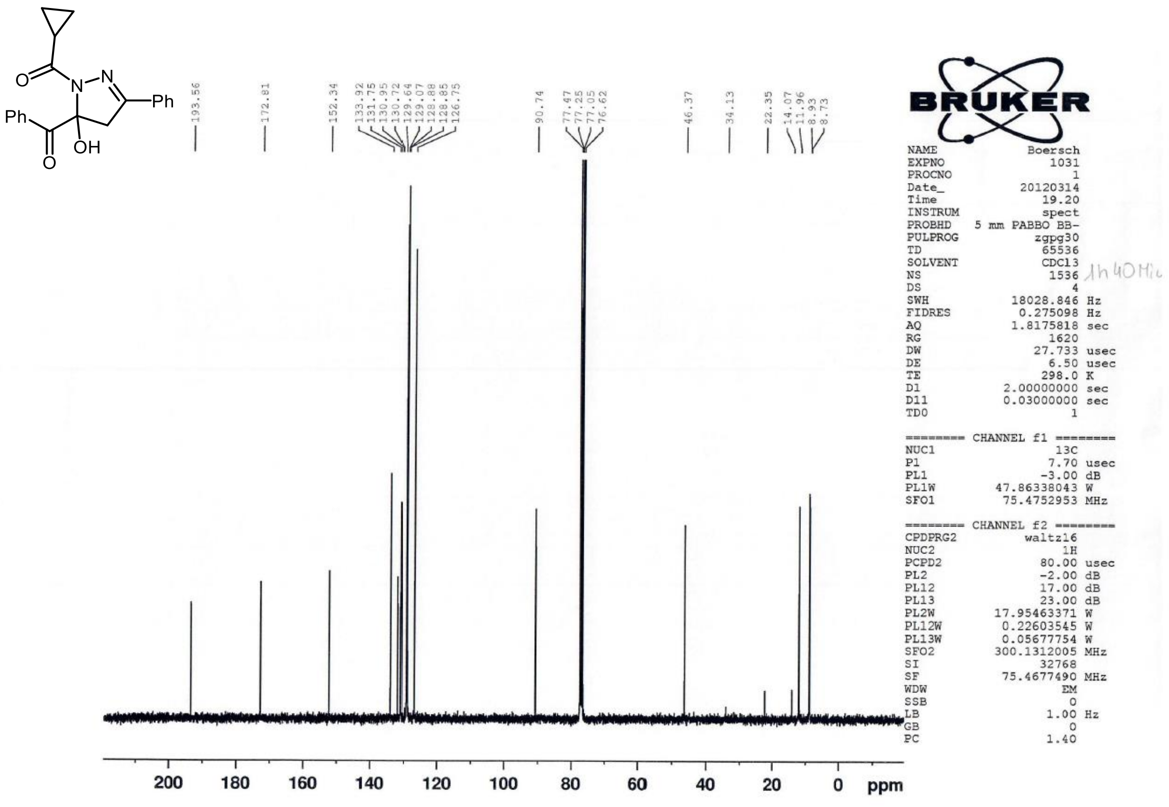

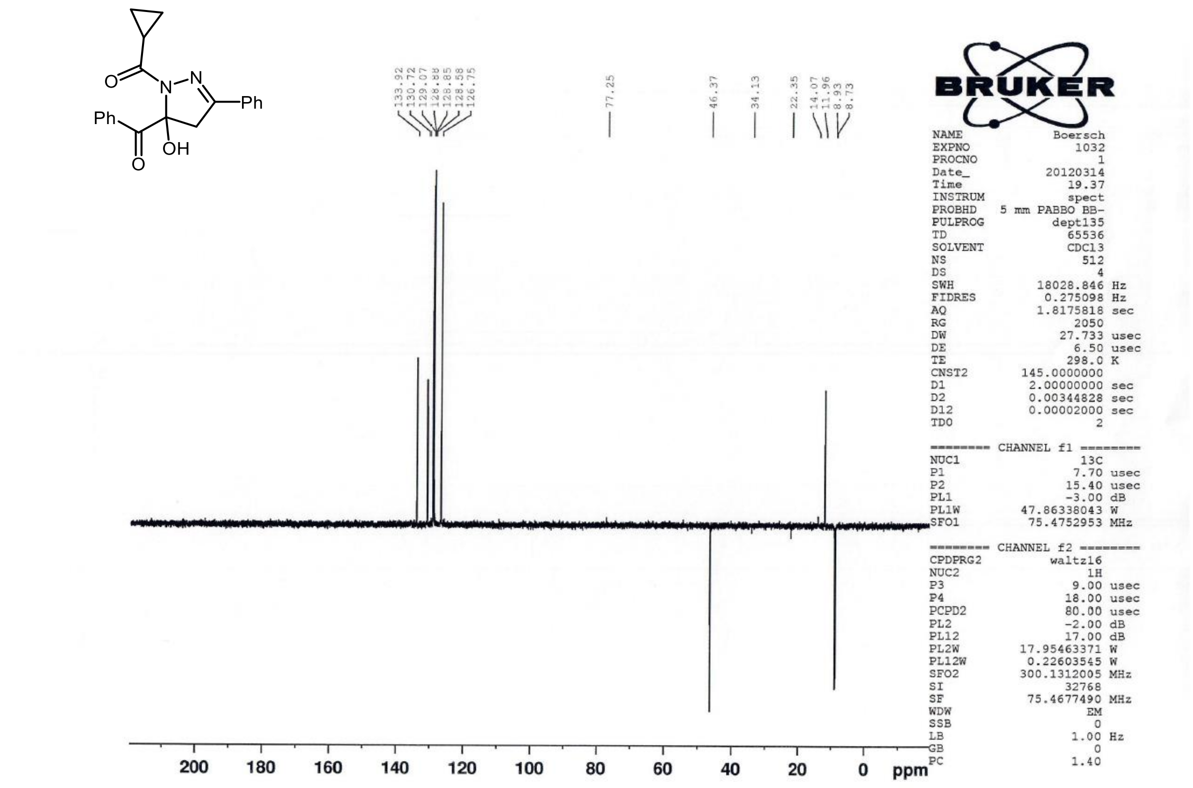

O
N
N
Ph
Ph
O
OH
133.92
130.72
129.07
128.88
128.85
128.58
126.75
77.25
46.37
34.13
22.35
14.07
11.96
8.93
8.73
BRUKER
NAME Boersch
EXPNO 1032
PROCNO 1
Date_ 20120314
Time 19.37
INSTRUM spect
PROBHD 5 mm PABBO BB-
PULPROG dept135
TD 65536
SOLVENT CDCl3
NS 512
DS 4
SWH 18028.846 Hz
FIDRES 0.275098 Hz
AQ 1.8175818 sec
RG 2050
DW 27.733 usec
DE 6.50 usec
TE 298.0 K
CNST2 145.0000000
D1 2.00000000 sec
D2 0.00344828 sec
D12 0.00002000 sec
TD0 2
======== CHANNEL f1 ========
NUC1 13C
P1 7.70 usec
P2 15.40 usec
PL1 -3.00 dB
PL1W 47.86338043 W
SFO1 75.4752953 MHz
======== CHANNEL f2 ========
CPDPRG2 waltz16
NUC2 1H
P3 9.00 usec
P4 18.00 usec
PCPD2 80.00 usec
PL2 -2.00 dB
PL12 17.00 dB
PL2W 17.95463371 W
PL12W 0.22603545 W
SFO2 300.1312005 MHz
SI 32768
SF 75.4677490 MHz
WDW EM
SSB 0
LB 1.00 Hz
GB 0
PC 1.40
200
180
160
140
120
100
80
60
40
20
0
ppm

5.10 1-(5-Benzoyl-5-hydroxy-3-phenyl-4,5-dihydro-1*H*-pyrazol-1-yl)-2,2-dimethylpropan-1-one (5j)

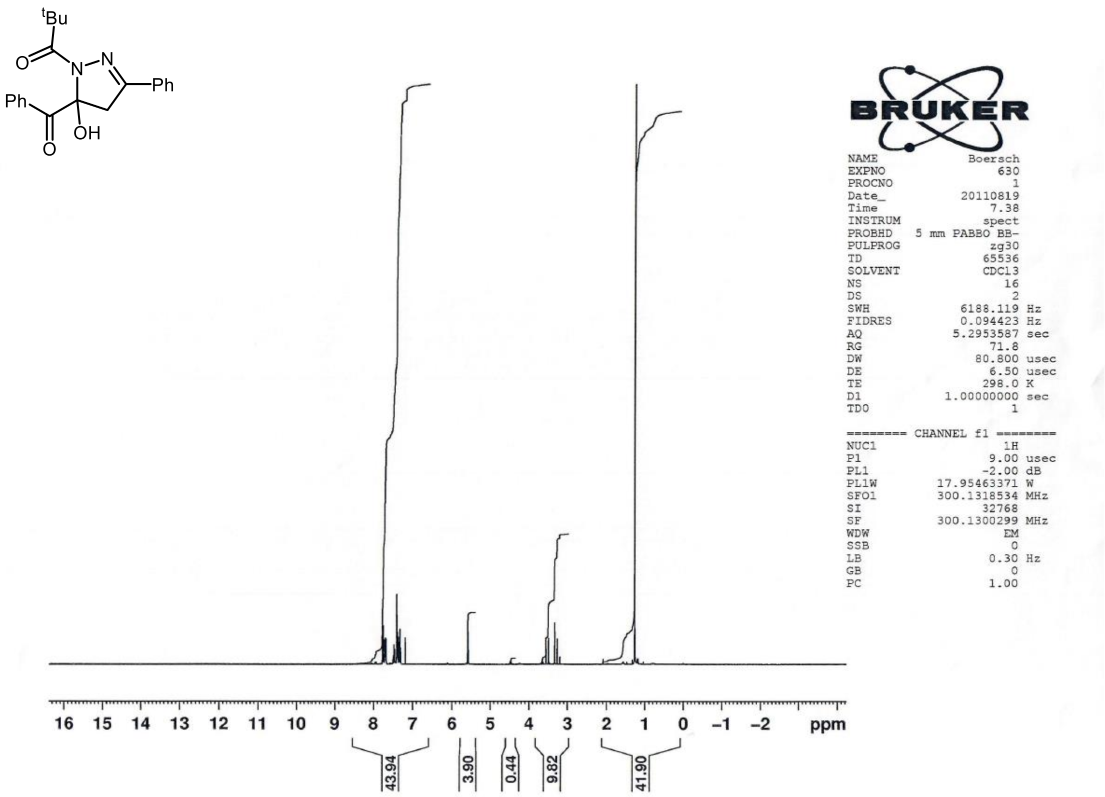

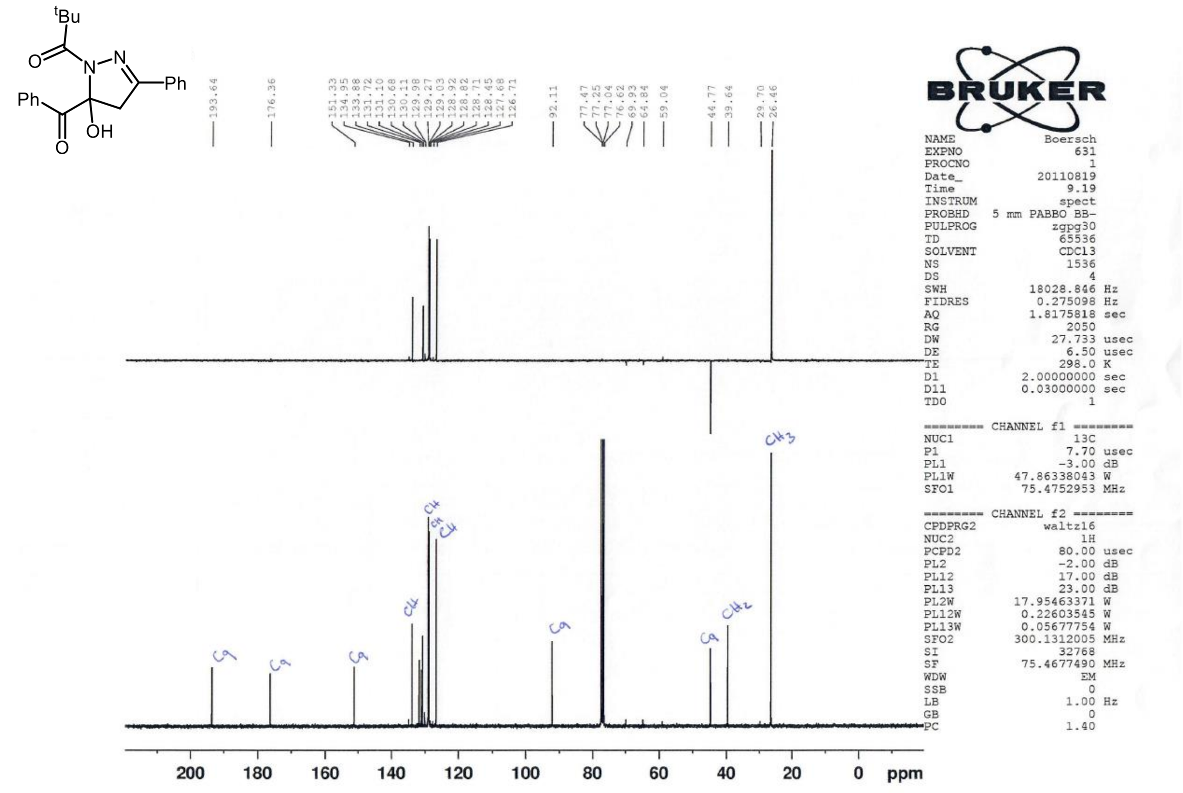

5.11 1-(5-Benzoyl-5-hydroxy-3-phenyl-4,5-dihydro-1*H*-pyrazol-1-yl)butan-1-one (5k)

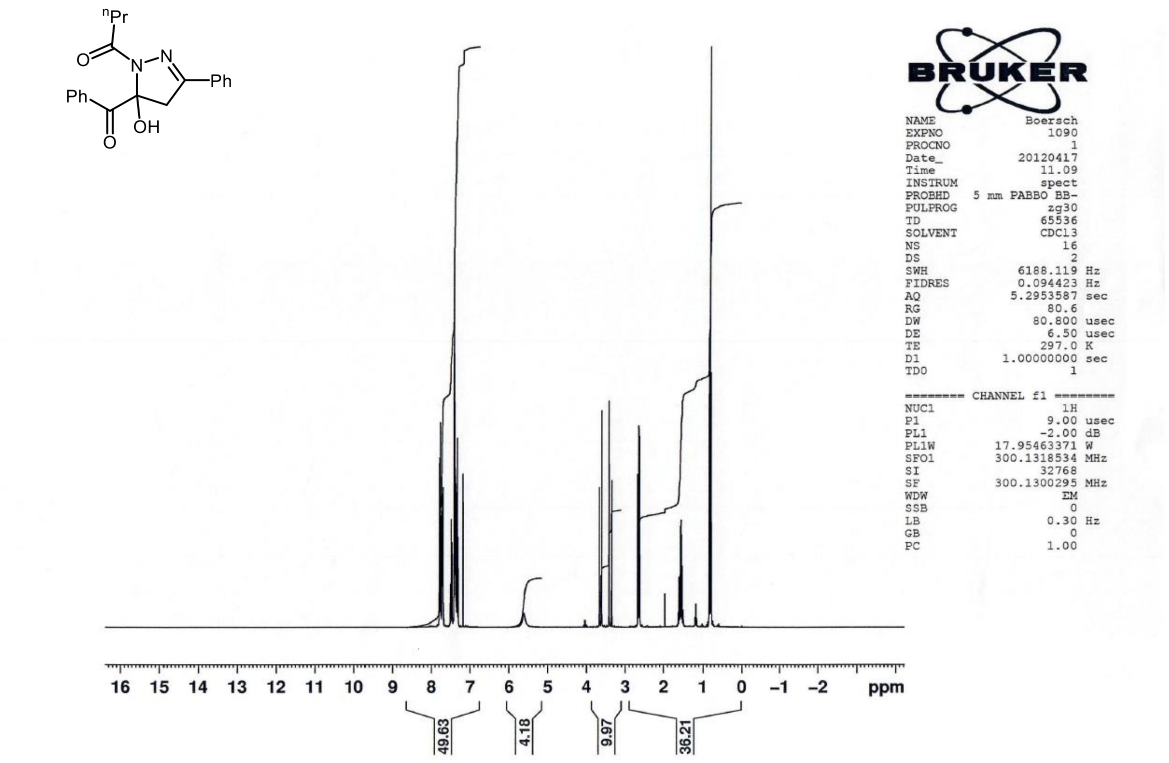

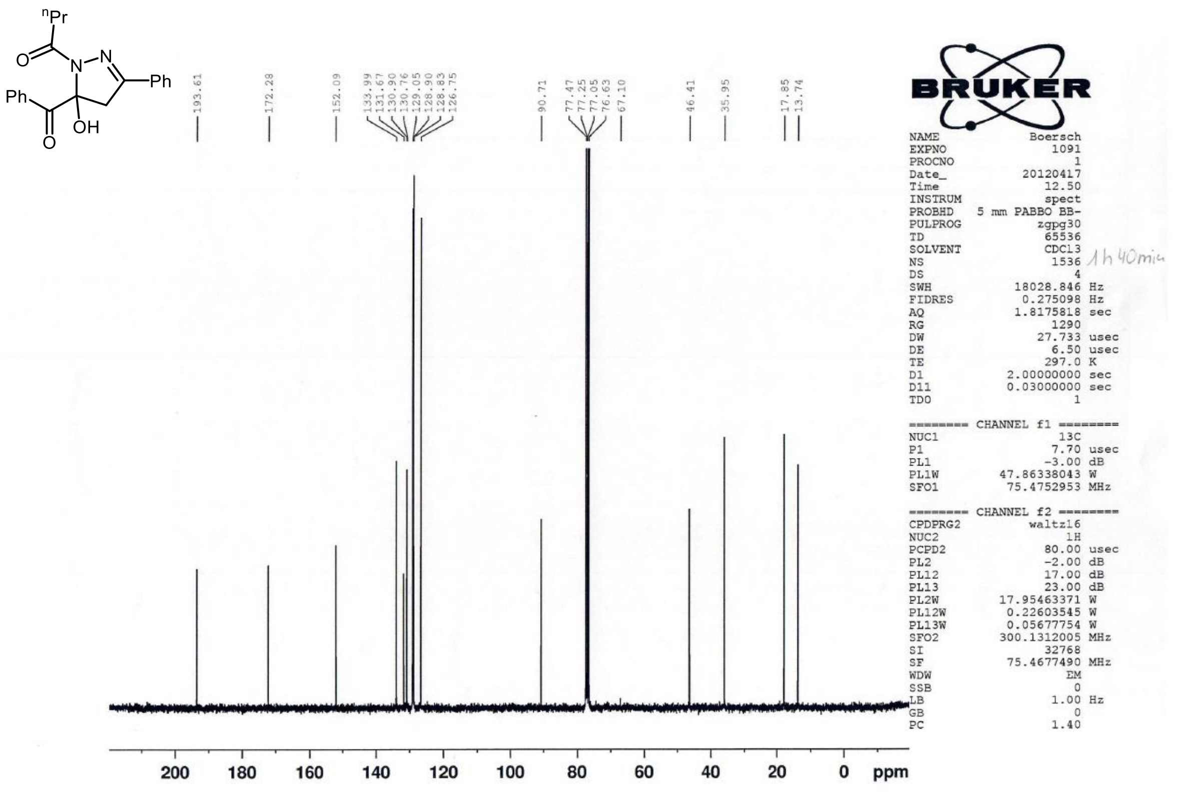

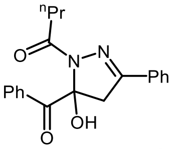

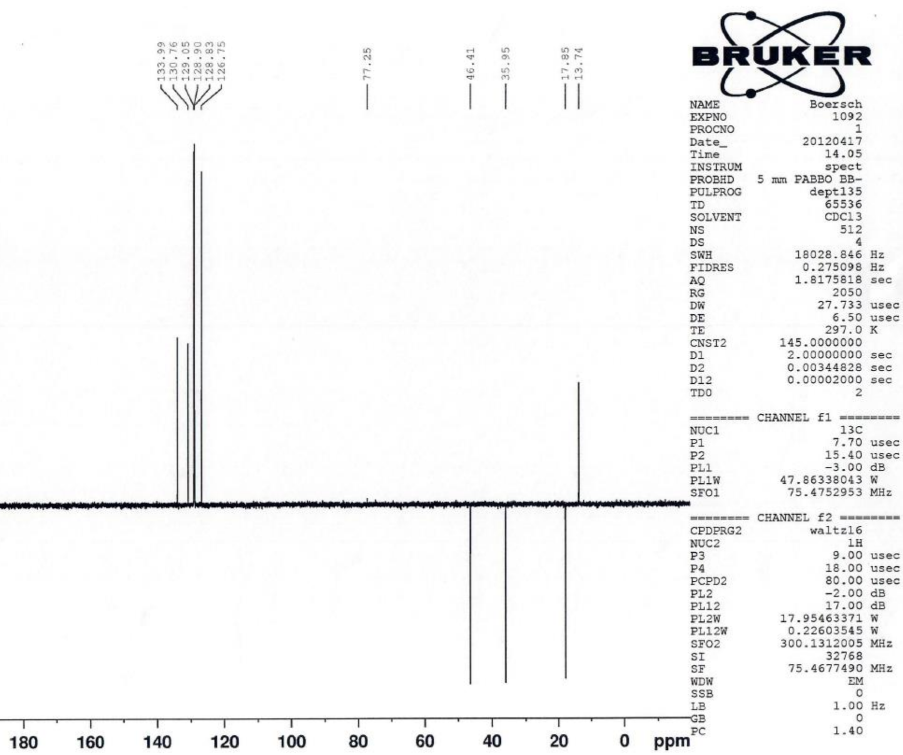
133.99
130.76
129.05
128.90
128.83
126.75
77.25
46.41
35.95
17.85
13.74
BRUKER
NAME Boersch
EXPNO 1092
PROCNO 1
Date_ 20120417
Time 14.05
INSTRUM spect
PROBHD 5 mm PABBO BB-
PULPROG dept135
TD 65536
SOLVENT CDCl3
NS 512
DS 4
SWH 18028.846 Hz
FIDRES 0.275098 Hz
AQ 1.8175818 sec
RG 2050
DW 27.733 usec
DE 6.50 usec
TE 297.0 K
CNST2 145.0000000
D1 2.00000000 sec
D2 0.00344828 sec
D12 0.00002000 sec
TD0 2
======== CHANNEL f1 ========
NUC1 13C
P1 7.70 usec
P2 15.40 usec
PL1 -3.00 dB
PL1W 47.86338043 W
SFO1 75.4752953 MHz
======== CHANNEL f2 ========
CPDPRG2 waltz16
NUC2 1H
P3 9.00 usec
P4 18.00 usec
PCPD2 80.00 usec
PL2 -2.00 dB
PL12 17.00 dB
PL2W 17.95463371 W
PL12W 0.22603545 W
SFO2 300.1312005 MHz
SI 32768
SF 75.4677490 MHz
WDW EM
SSB 0
LB 1.00 Hz
GB 0
PC 1.40
200 180 160 140 120 100 80 60 40 20 0 ppm

5.12 (5-Hydroxy-3-(4-methoxyphenyl)-4,5-dihydro-1*H*-pyrazol-1,5-diyl)bis(phenylmethanone) (5l)

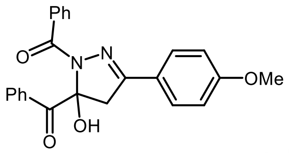

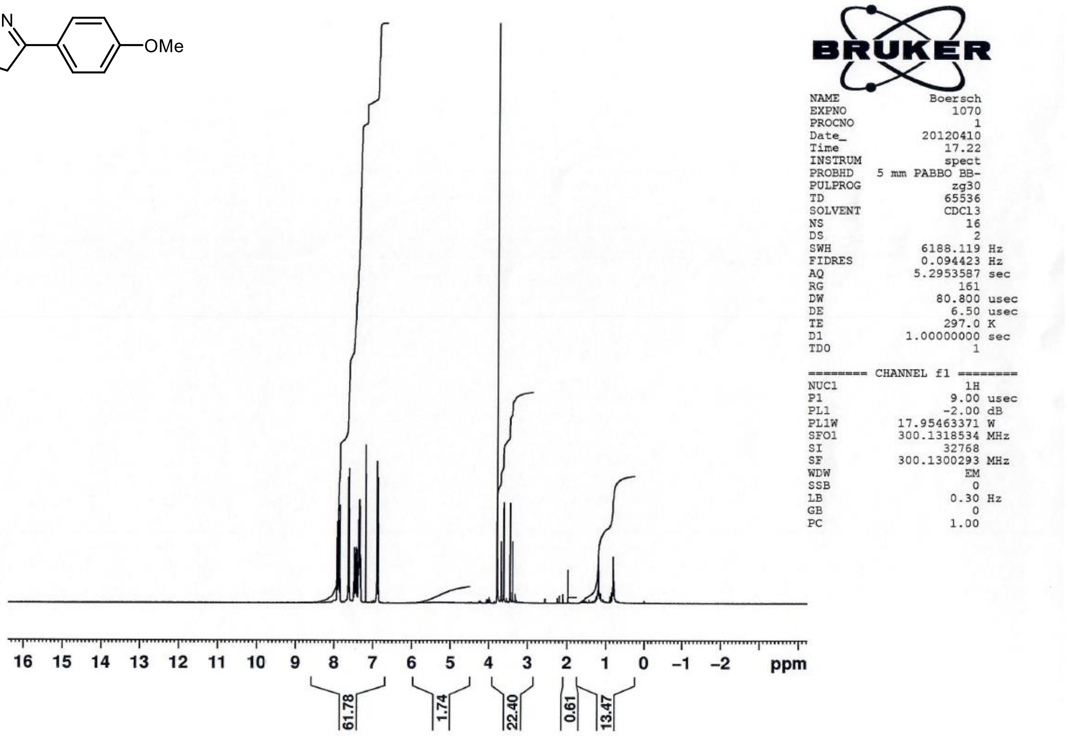

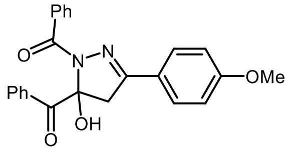

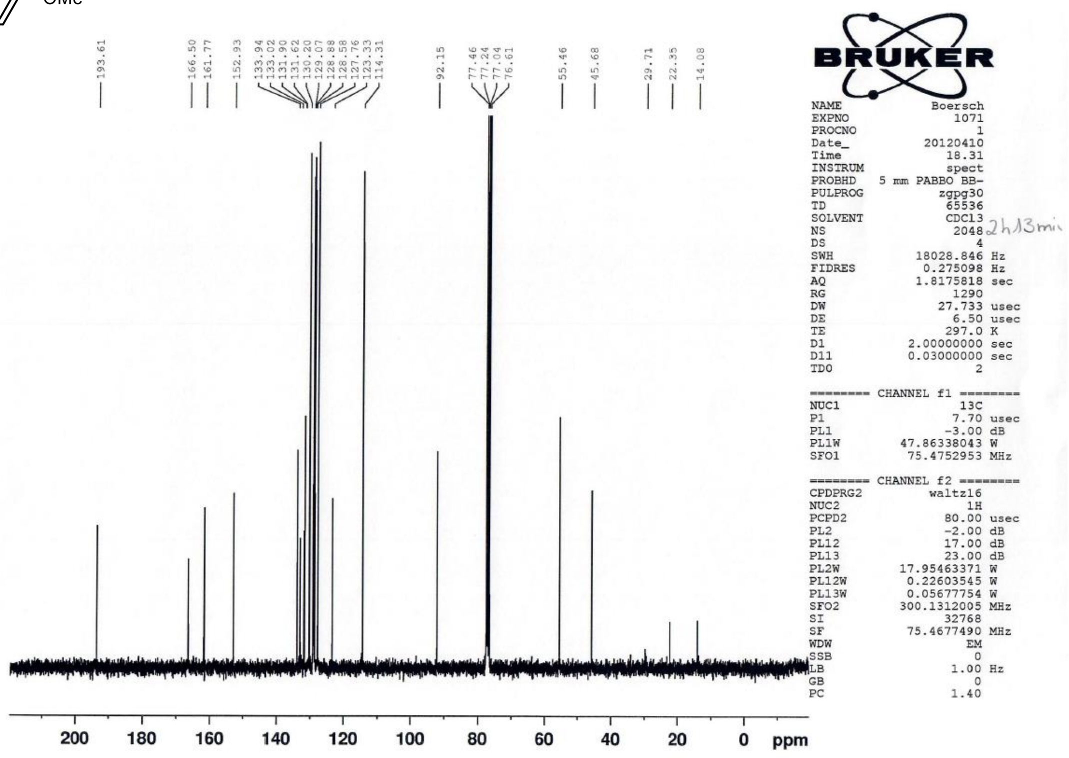

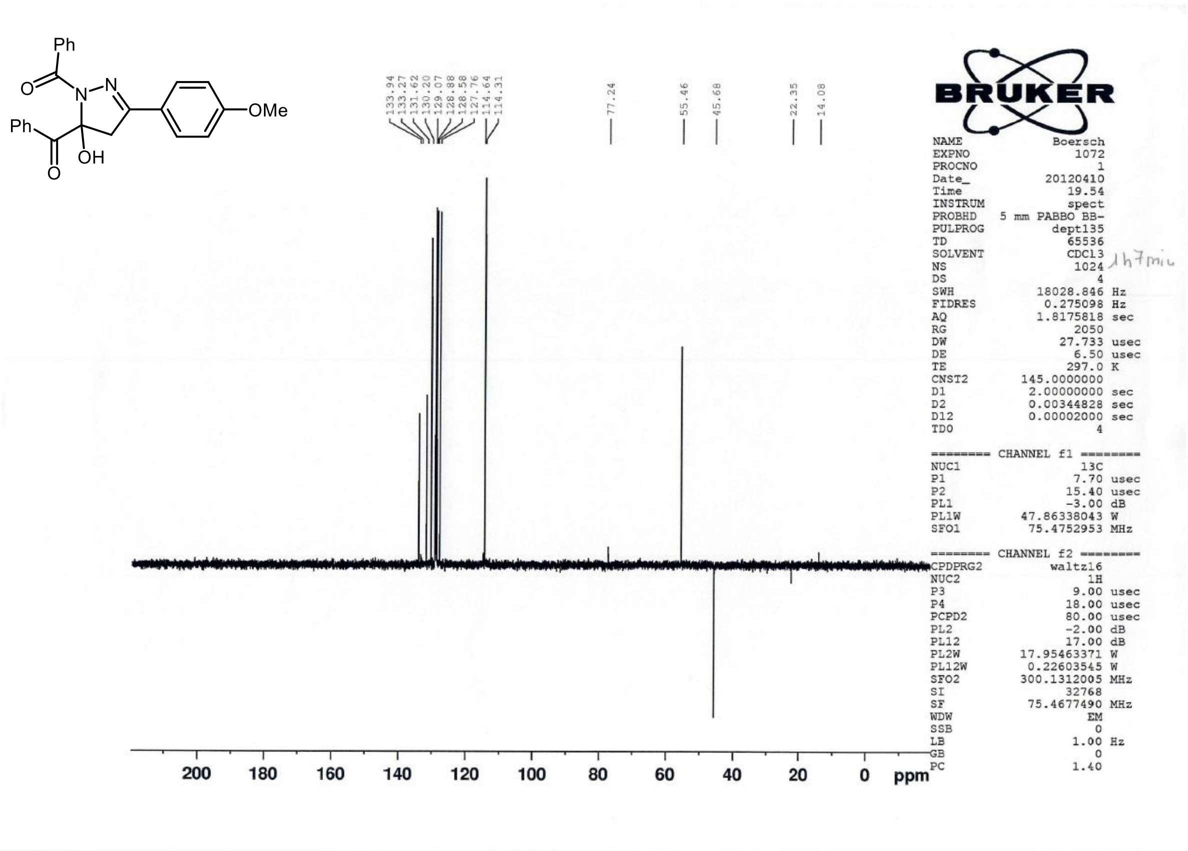
Ph
O
N
N
OMe
Ph
O
OH
133.94
133.27
131.62
130.20
129.07
128.88
128.58
127.76
114.64
114.31
77.24
55.46
45.68
22.35
14.08
BRUKER
NAME Boersch
EXPNO 1072
PROCNO 1
Date_ 20120410
Time 19.54
INSTRUM spect
PROBHD 5 mm PABBO BB-
PULPROG dept135
TD 65536
SOLVENT CDCl3
NS 1024
DS 4
SWH 18028.846 Hz
FIDRES 0.275098 Hz
AQ 1.8175818 sec
RG 2050
DW 27.733 usec
DE 6.50 usec
TE 297.0 K
CNST2 145.0000000
D1 2.00000000 sec
D2 0.00344828 sec
D12 0.00002000 sec
TD0 4
======== CHANNEL f1 ========
NUC1 13C
P1 7.70 usec
P2 15.40 usec
PL1 -3.00 dB
PL1W 47.86338043 W
SFO1 75.4752953 MHz
======== CHANNEL f2 ========
CPDPRG2 waltz16
NUC2 1H
P3 9.00 usec
P4 18.00 usec
PCPD2 80.00 usec
PL2 -2.00 dB
PL12 17.00 dB
PL2W 17.95463371 W
PL12W 0.22603545 W
SFO2 300.1312005 MHz
SI 32768
SF 75.4677490 MHz
WDW EM
SSB 0
LB 1.00 Hz
GB 0
PC 1.40
200
180
160
140
120
100
80
60
40
20
0
ppm

5.13 (3-(4-(*tert*-Butyl)phenyl)-5-hydroxy-4,5-dihydro-1*H*-pyrazol-1,5-diyl)bis(phenylmethanone) (5m)

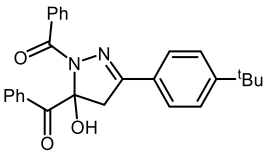

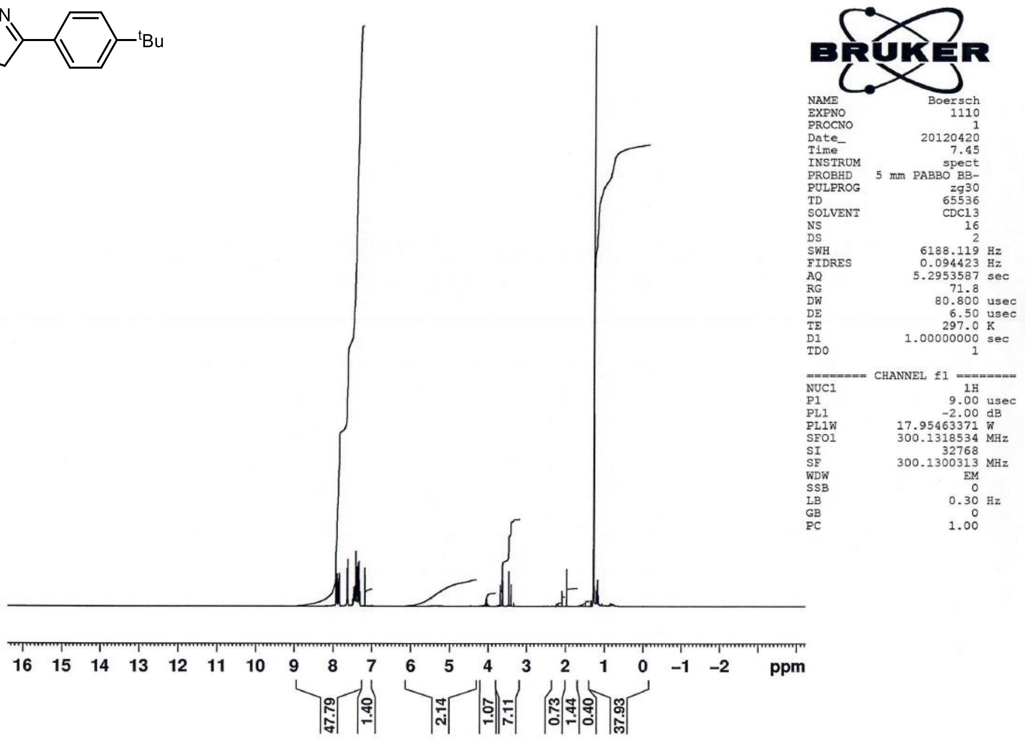

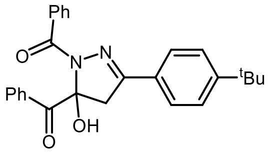

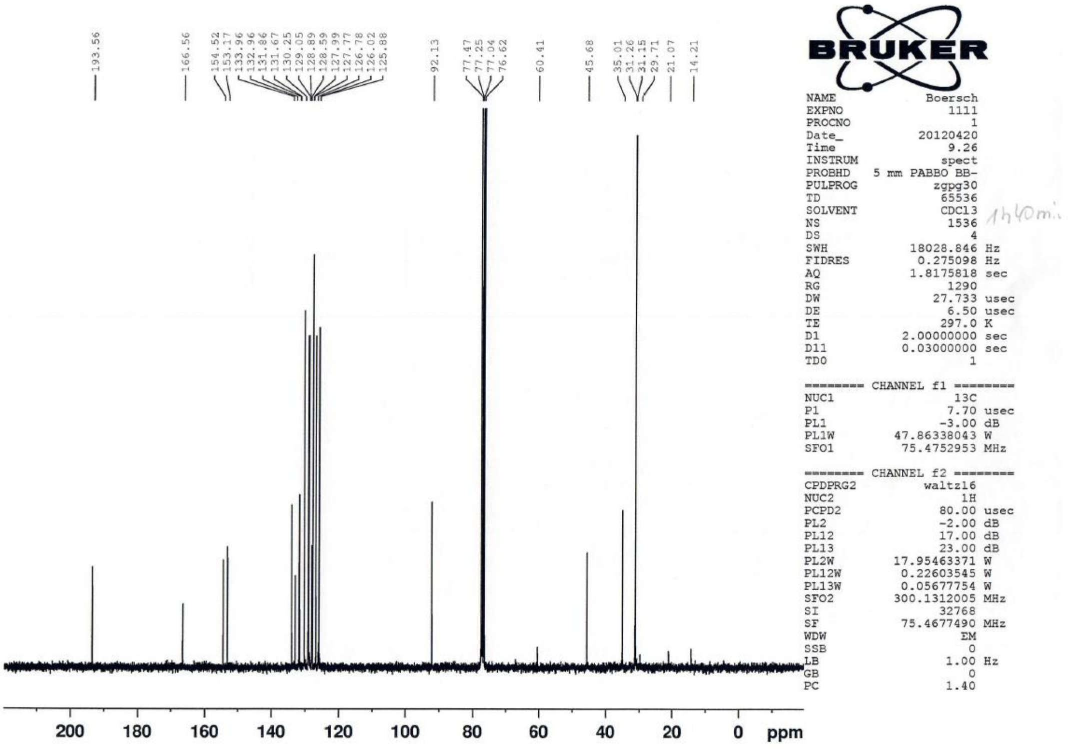

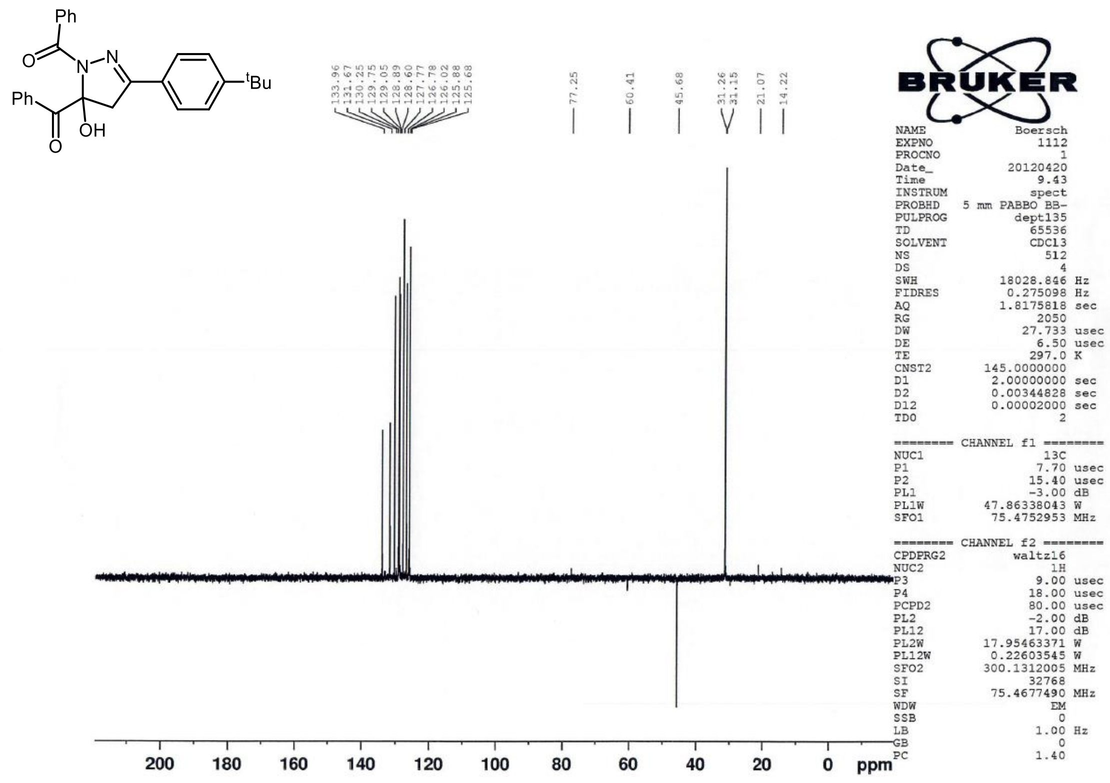
Ph
O
N
N
Ph
O
OH
tBu
133.96
131.67
130.25
129.75
129.05
128.89
128.60
127.77
127.78
126.02
125.88
125.68
77.25
60.41
45.68
31.26
31.15
21.07
14.22
BRUKER
NAME Boersch
EXPNO 1112
PROCNO 1
Date_ 20120420
Time 9.43
INSTRUM spect
PROBHD 5 mm PABBO BB-
PULPROG dept135
TD 65536
SOLVENT CDCl3
NS 512
DS 4
SWH 18028.846 Hz
FIDRES 0.275098 Hz
AQ 1.8175818 sec
RG 2050
DW 27.733 usec
DE 6.50 usec
TE 297.0 K
CNST2 145.0000000
D1 2.00000000 sec
D2 0.00344828 sec
D12 0.00002000 sec
TD0 2
======== CHANNEL f1 ========
NUC1 13C
P1 7.70 usec
P2 15.40 usec
PL1 -3.00 dB
PL1W 47.86338043 W
SFO1 75.4752953 MHz
======== CHANNEL f2 ========
CPDPRG2 waltz16
NUC2 1H
P3 9.00 usec
P4 18.00 usec
PCPD2 80.00 usec
PL2 -2.00 dB
PL12 17.00 dB
PL2W 17.95463371 W
PL12W 0.22603545 W
SFO2 300.1312005 MHz
SI 32768
SF 75.4677490 MHz
WDW EM
SSB 0
LB 1.00 Hz
GB 0
PC 1.40
200
180
160
140
120
100
80
60
40
20
0
ppm

5.14 (3-(4-Fluorophenyl)-5-hydroxy-4,5-dihydro-1*H*-pyrazol-1,5-diyl)bis(phenylmethanone) (5n)

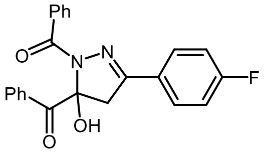

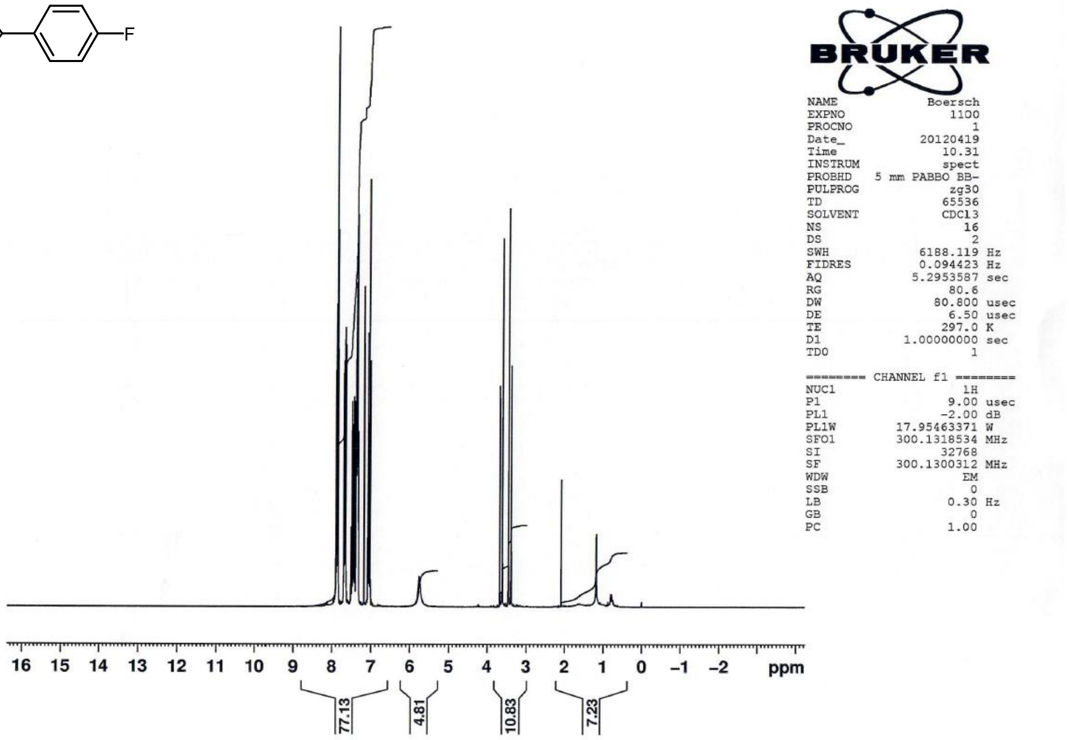

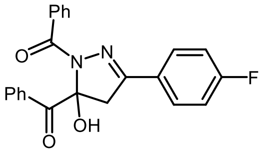

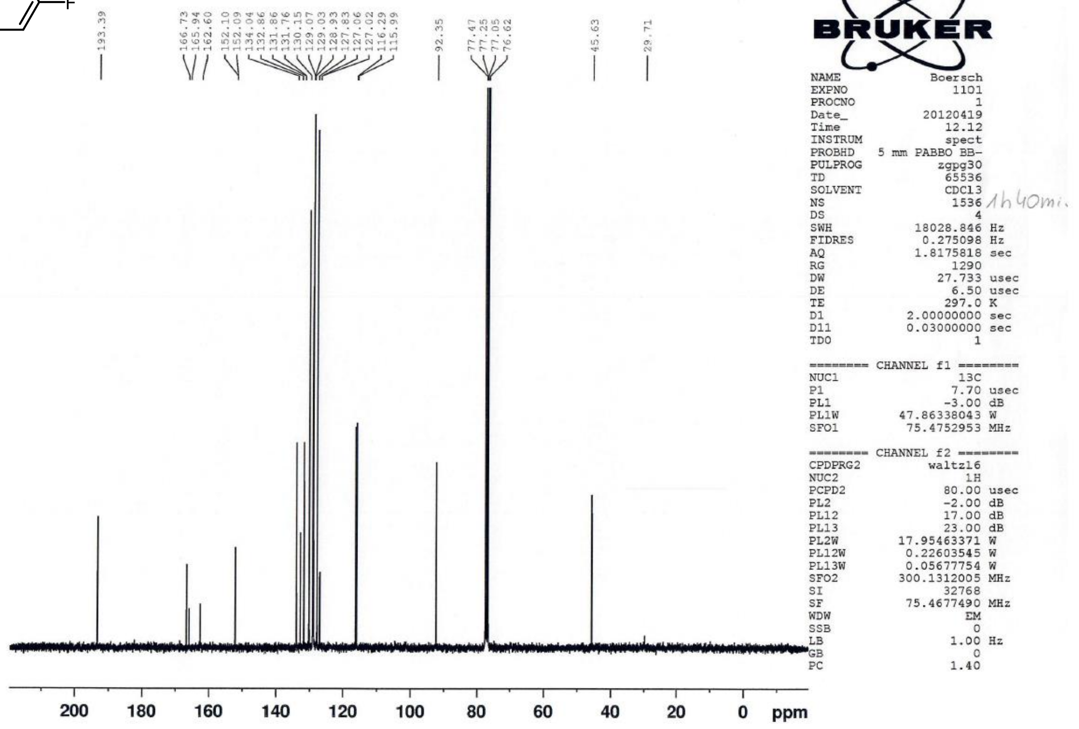

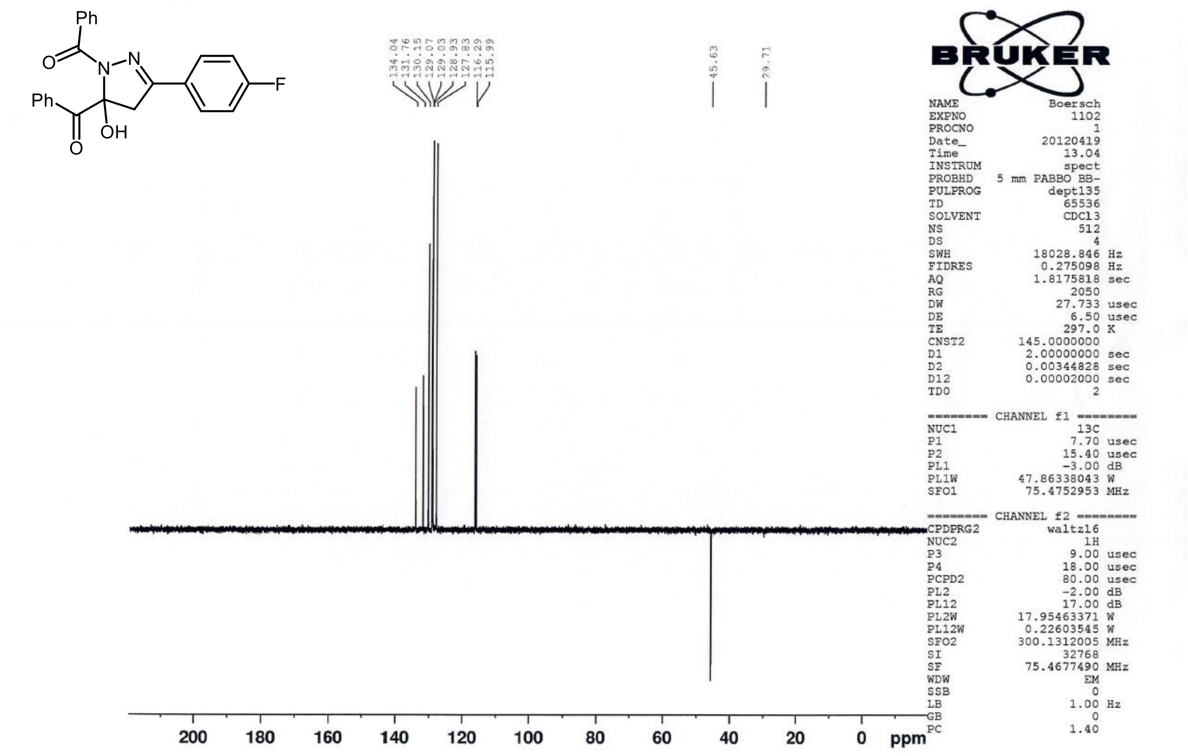

Ph
O
N
N
F
Ph
OH
O
134.04
131.76
130.15
129.07
129.03
128.93
127.83
116.29
115.99
45.63
29.71
BRUKER
NAME Boersch
EXPNO 1102
PROCNO 1
Date_ 20120419
Time 13.04
INSTRUM spect
PROBHD 5 mm PABBO BB-
PULPROG dept135
TD 65536
SOLVENT CDCl3
NS 512
DS 4
SWH 18028.846 Hz
FIDRES 0.275098 Hz
AQ 1.8175818 sec
RG 2050
DW 27.733 usec
DE 6.50 usec
TE 297.0 K
CNST2 145.0000000
D1 2.00000000 sec
D2 0.00344828 sec
D12 0.00002000 sec
TD0 2
======== CHANNEL f1 ========
NUC1 13C
P1 7.70 usec
P2 15.40 usec
PL1 -3.00 dB
PL1W 47.86338043 W
SFO1 75.4752953 MHz
======== CHANNEL f2 ========
CPDPRG2 waltz16
NUC2 1H
P3 9.00 usec
P4 18.00 usec
PCPD2 80.00 usec
PL2 -2.00 dB
PL12 17.00 dB
PL2W 17.95463371 W
PL12W 0.22603545 W
SFO2 300.1312005 MHz
SI 32768
SF 75.4677490 MHz
WDW EM
SSB 0
LB 1.00 Hz
GB 0
PC 1.40
200
180
160
140
120
100
80
60
40
20
0
ppm

5.15 4-(1,5-Dibenzoyl-5-hydroxy-4,5-dihydro-1*H*-pyrazol-3-yl)benzonitrile (5o)

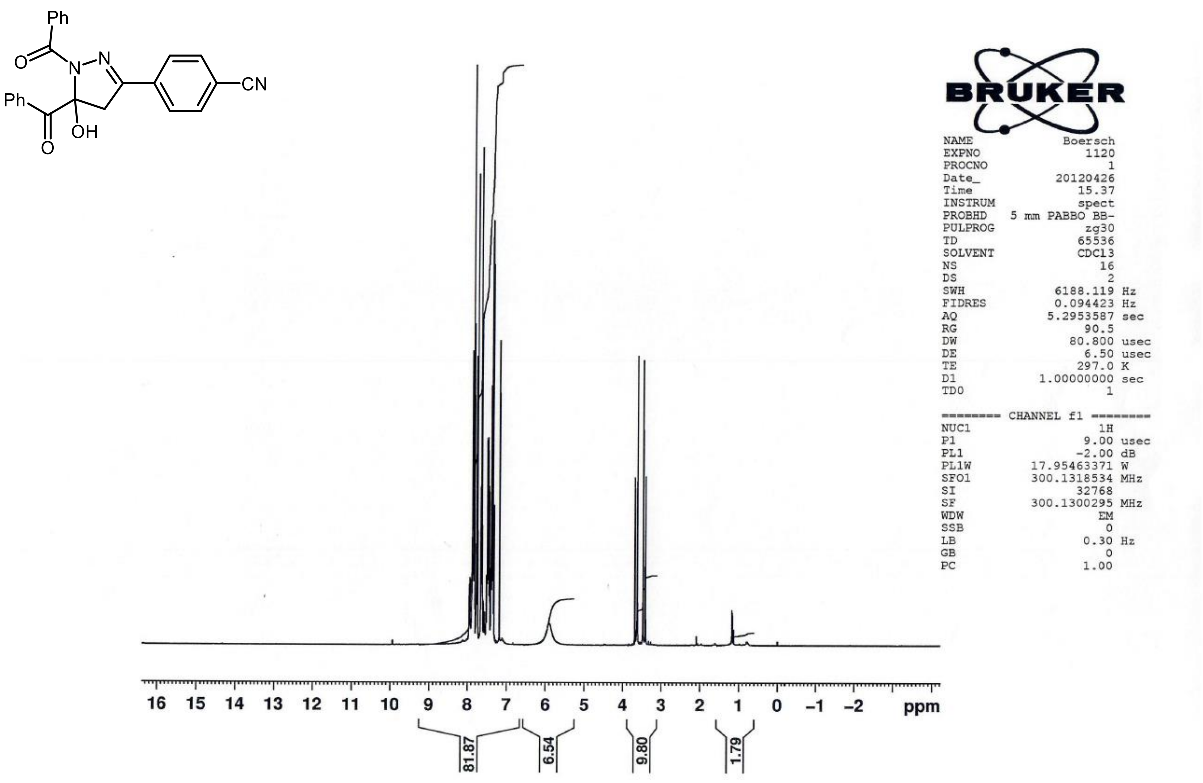

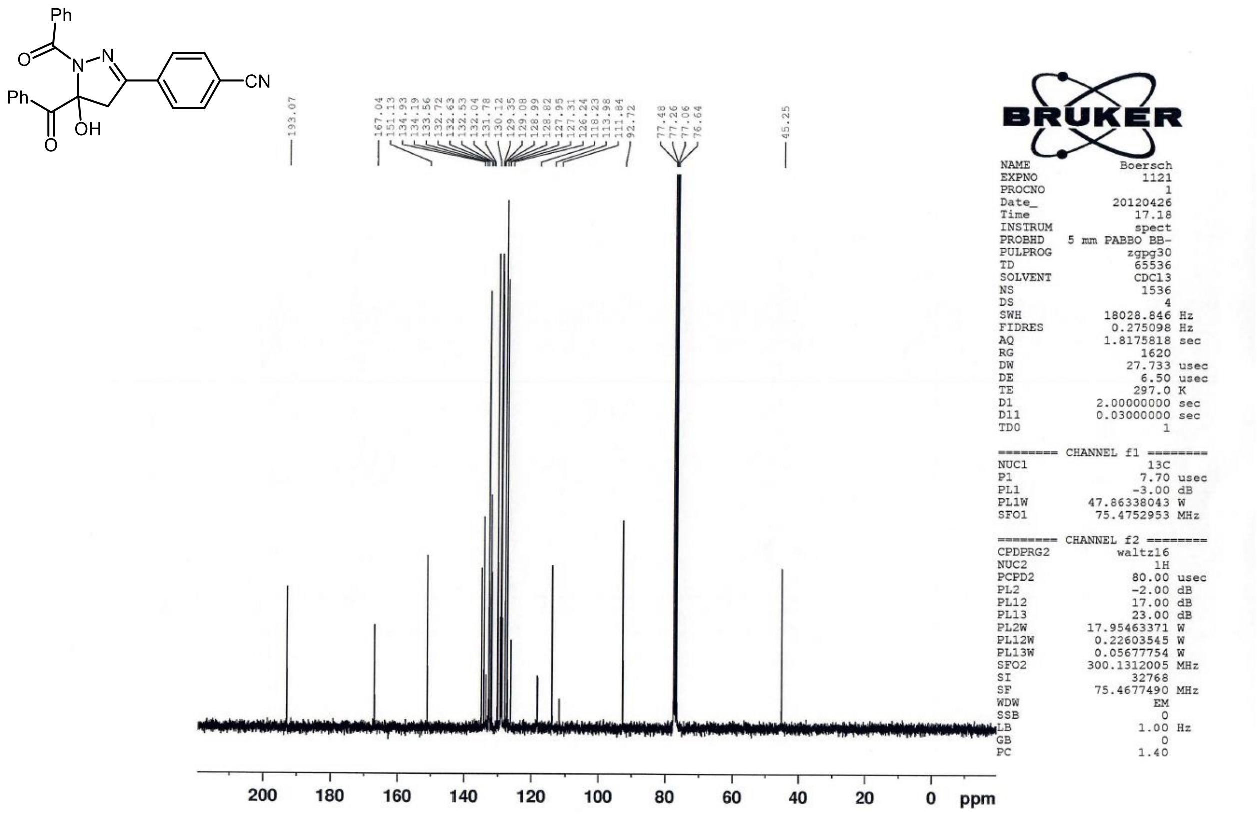

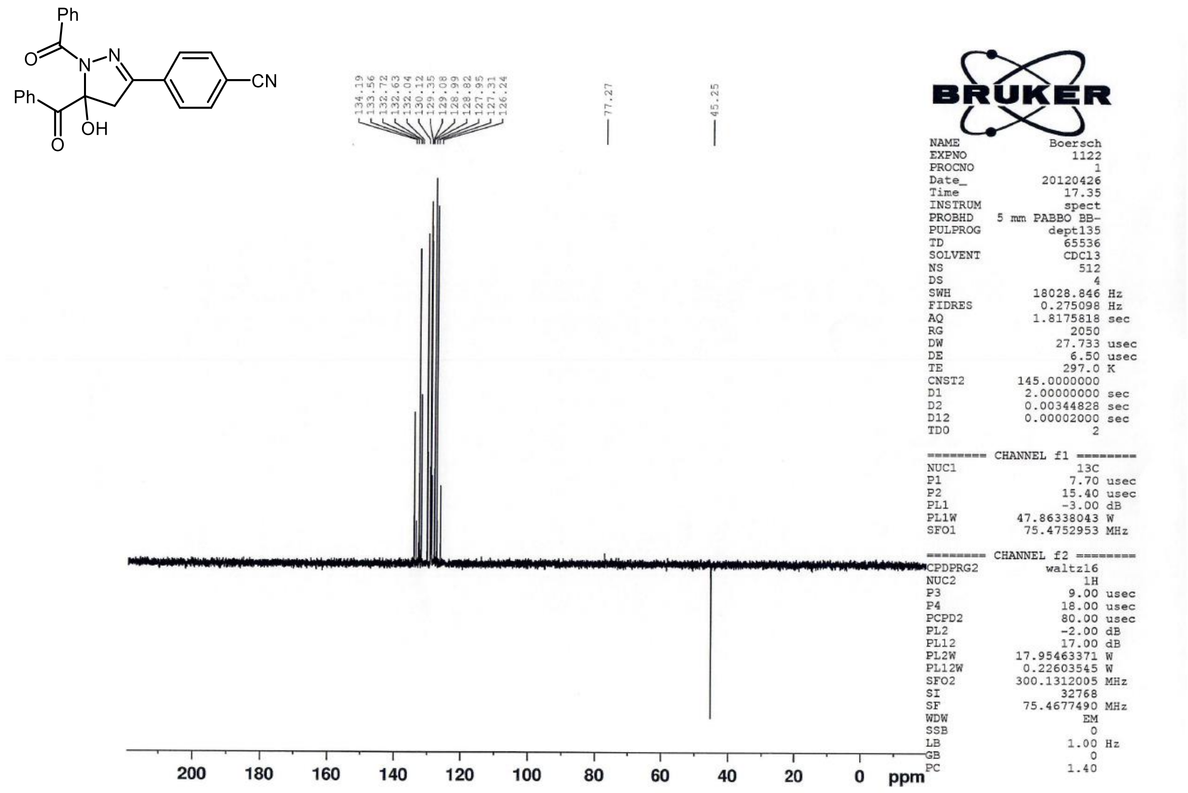
Ph
O
N
N
CN
Ph
O
OH
134.19
133.56
132.72
132.63
132.04
130.12
129.35
129.08
128.99
128.82
127.95
127.31
126.24
77.27
45.25
BRUKER
NAME Boersch
EXPNO 1122
PROCNO 1
Date_ 20120426
Time 17.35
INSTRUM spect
PROBHD 5 mm PABBO BB-
PULPROG dept135
TD 65536
SOLVENT CDCl3
NS 512
DS 4
SWH 18028.846 Hz
FIDRES 0.275098 Hz
AQ 1.8175818 sec
RG 2050
DW 27.733 usec
DE 6.50 usec
TE 297.0 K
CNST2 145.0000000
D1 2.00000000 sec
D2 0.00344828 sec
D12 0.00002000 sec
TD0 2
======== CHANNEL f1 ========
NUC1 13C
P1 7.70 usec
P2 15.40 usec
PL1 -3.00 dB
PL1W 47.86338043 W
SFO1 75.4752953 MHz
======== CHANNEL f2 ========
CPDPRG2 waltz16
NUC2 1H
P3 9.00 usec
P4 18.00 usec
PCPD2 80.00 usec
PL2 -2.00 dB
PL12 17.00 dB
PL2W 17.95463371 W
PL12W 0.22603545 W
SFO2 300.1312005 MHz
SI 32768
SF 75.4677490 MHz
WDW EM
SSB 0
LB 1.00 Hz
GB 0
PC 1.40
200 180 160 140 120 100 80 60 40 20 0 ppm

5.16 (1-Benzoyl-5-hydroxy-3-phenyl-4,5-dihydro-1*H*-pyrazol-5-yl)(mesityl)methanone (5p)

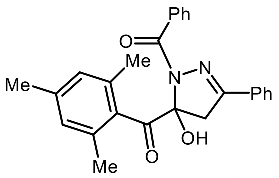

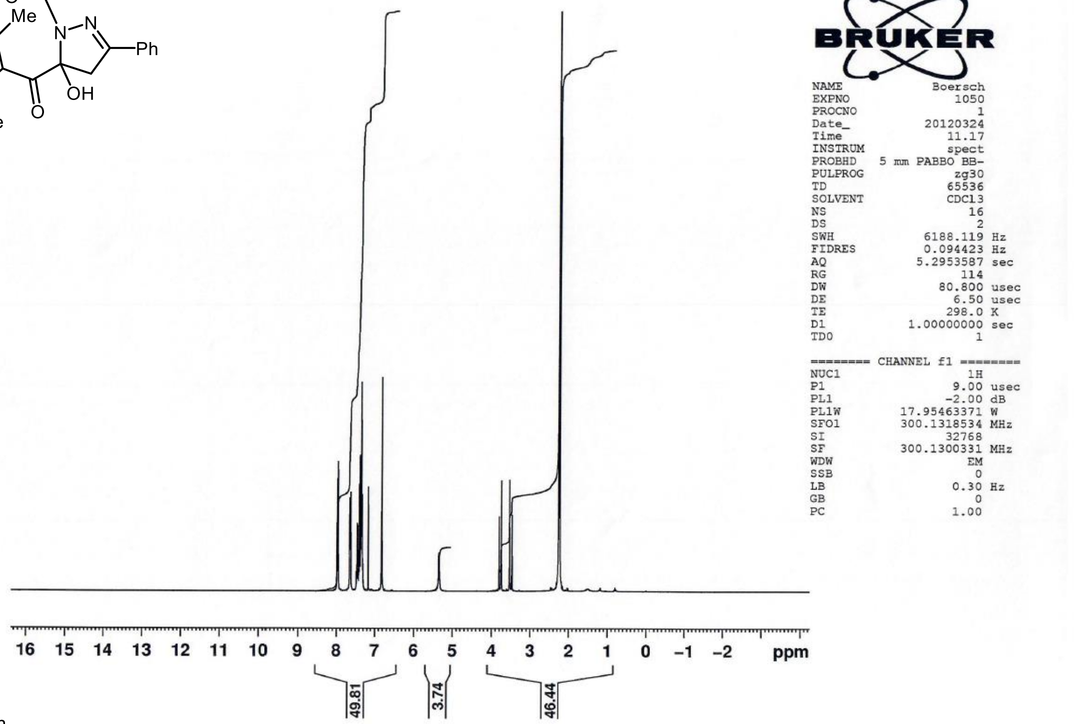

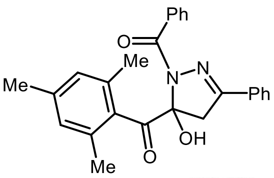

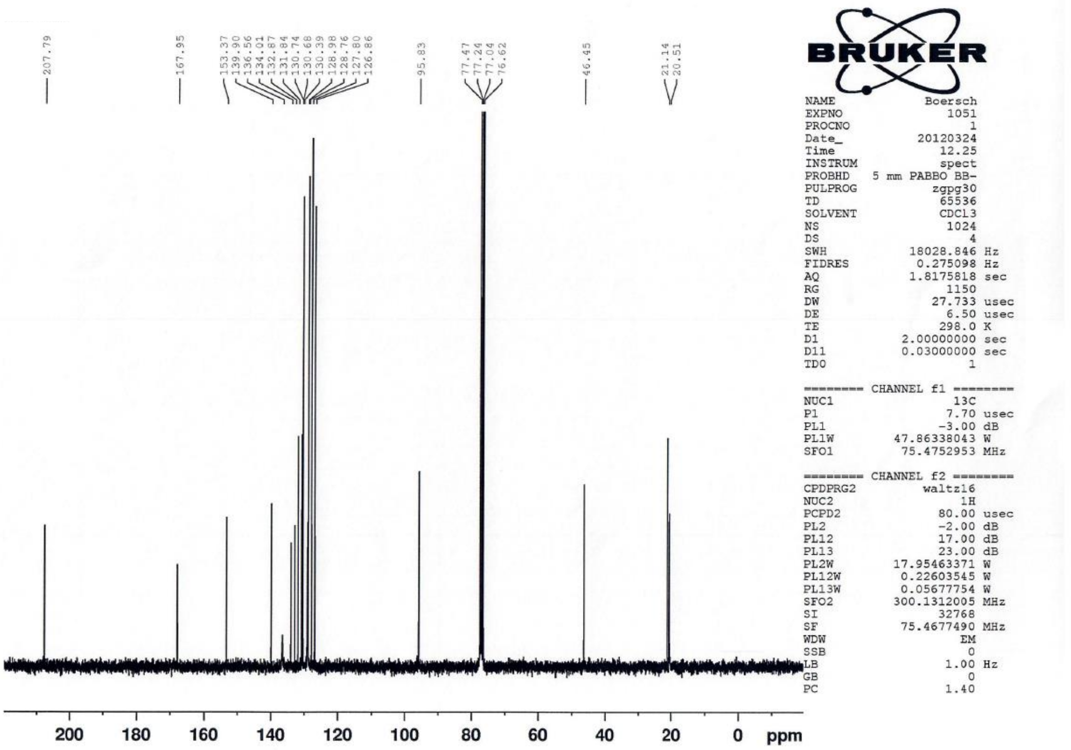

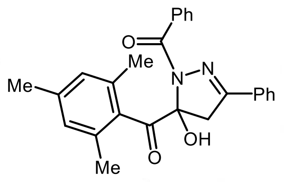

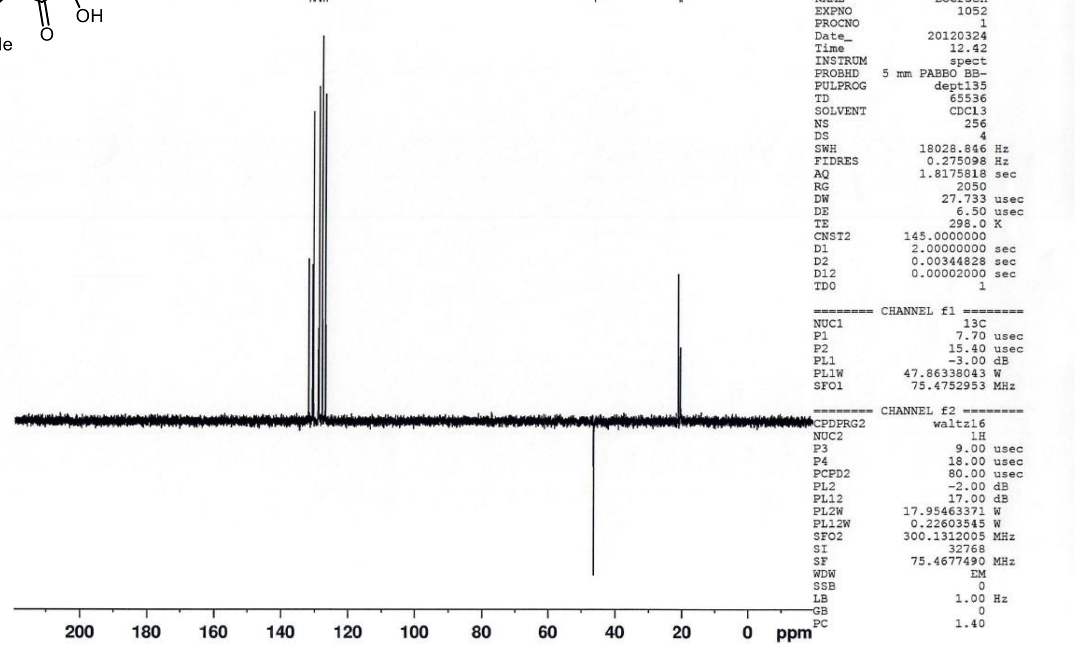
131.84
130.68
130.39
128.97
128.76
127.80
126.86
46.45
21.14
20.51
NAME Boersch
EXPNO 1052
PROCNO 1
Date_ 20120324
Time 12.42
INSTRUM spect
PROBHD 5 mm PABBO BB-
PULPROG dept135
TD 65536
SOLVENT CDCl3
NS 256
DS 4
SWH 18028.846 Hz
FIDRES 0.275098 Hz
AQ 1.8175818 sec
RG 2050
DW 27.733 usec
DE 6.50 usec
TE 298.0 K
CNST2 145.0000000
D1 2.00000000 sec
D2 0.00344828 sec
D12 0.00002000 sec
TD0 1
======== CHANNEL f1 ========
NUC1 13C
P1 7.70 usec
P2 15.40 usec
PL1 -3.00 dB
PL1W 47.86338043 W
SFO1 75.4752953 MHz
======== CHANNEL f2 ========
CPDPRG2 waltz16
NUC2 1H
P3 9.00 usec
P4 18.00 usec
PCPD2 80.00 usec
PL2 -2.00 dB
PL12 17.00 dB
PL2W 17.95463371 W
PL12W 0.22603545 W
SFO2 300.1312005 MHz
SI 32768
SF 75.4677490 MHz
WDW EM
SSB 0
LB 1.00 Hz
GB 0
PC 1.40
200 180 160 140 120 100 80 60 40 20 0 ppm

5.17 (1-Benzoyl-5-hydroxy-3-phenyl-4,5-dihydro-1*H*-pyrazol-5-yl)(thien-2-yl)methanone (5q)

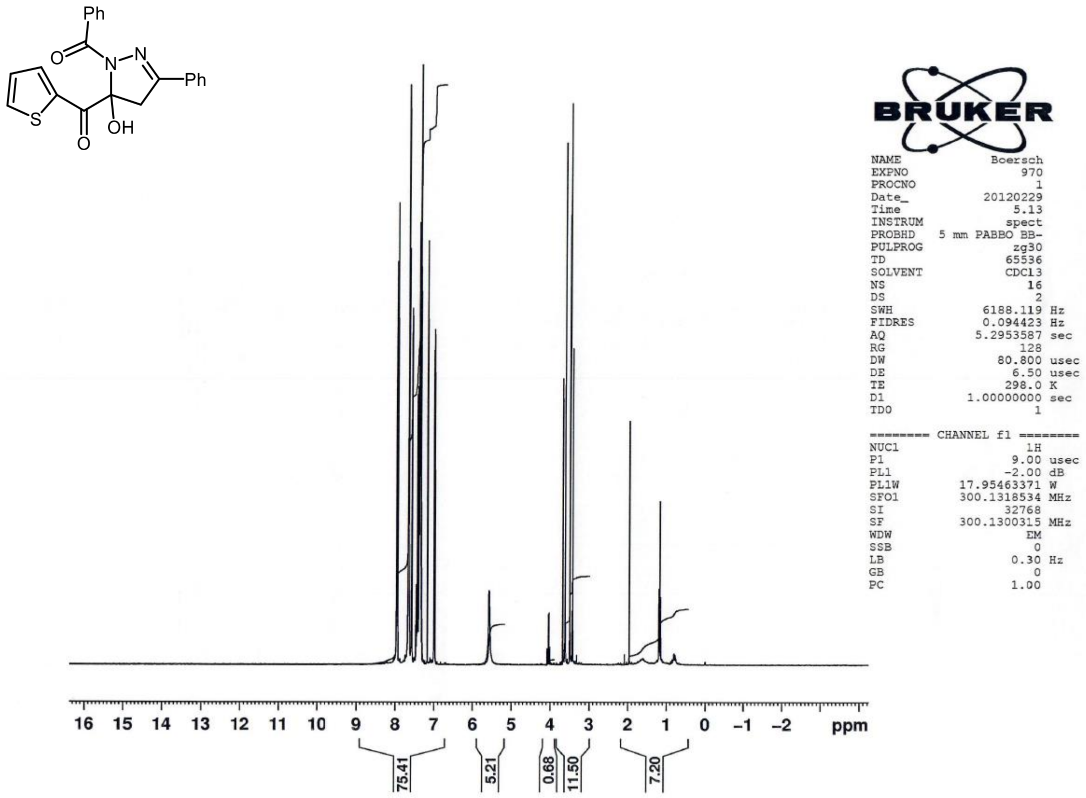

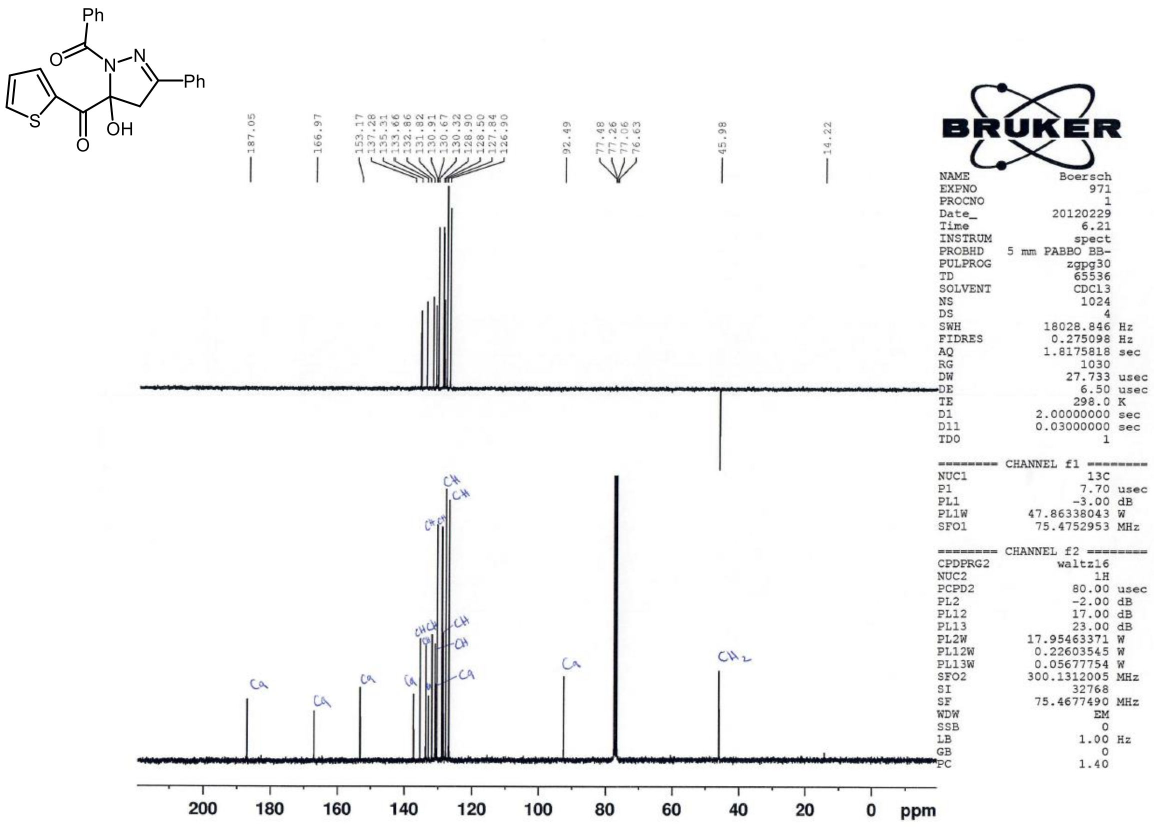

5.18 (5-Hydroxy-3-(4-methoxyphenyl)-1-(thiophen-2-carbonyl)-4,5-dihydro-1*H*-pyrazol-5-yl) (mesityl)methanone (5r)

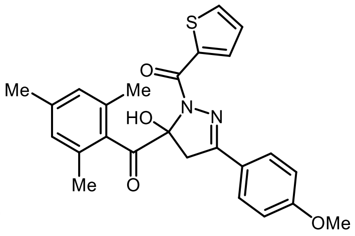

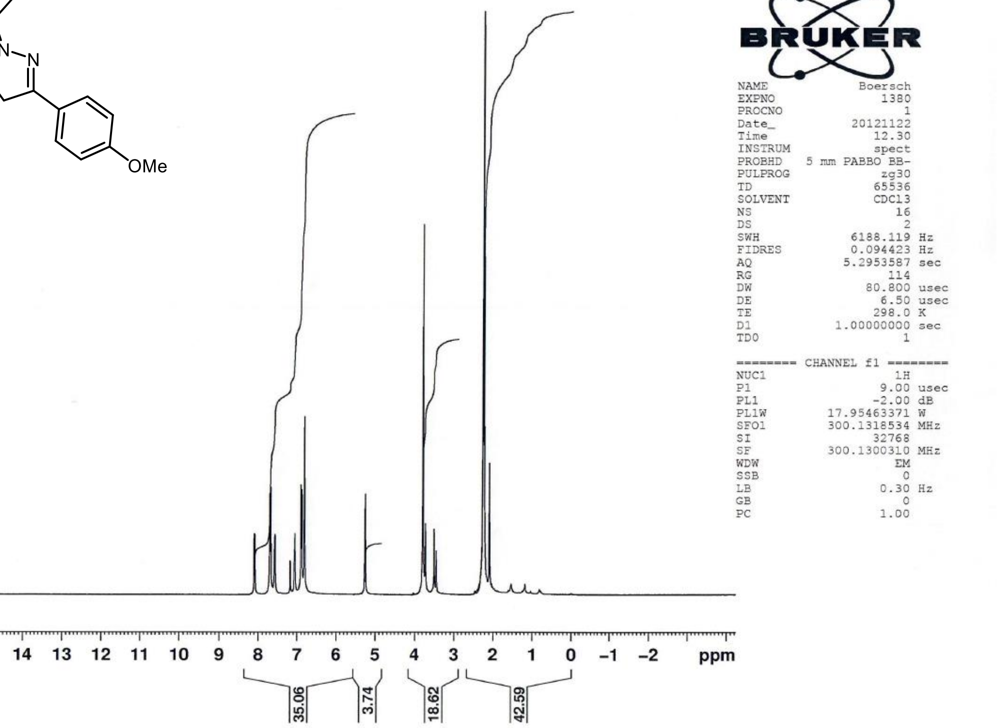

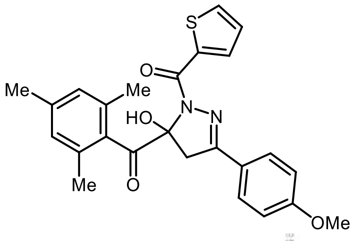

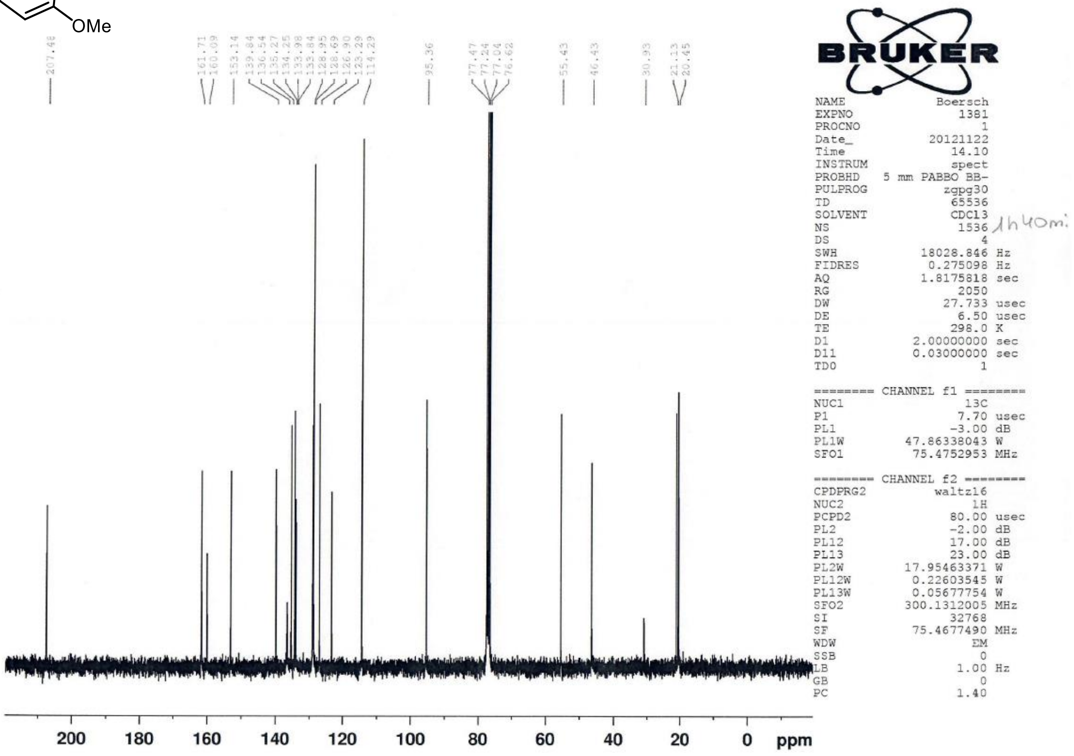

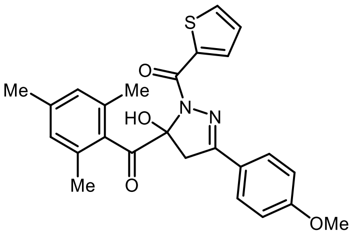

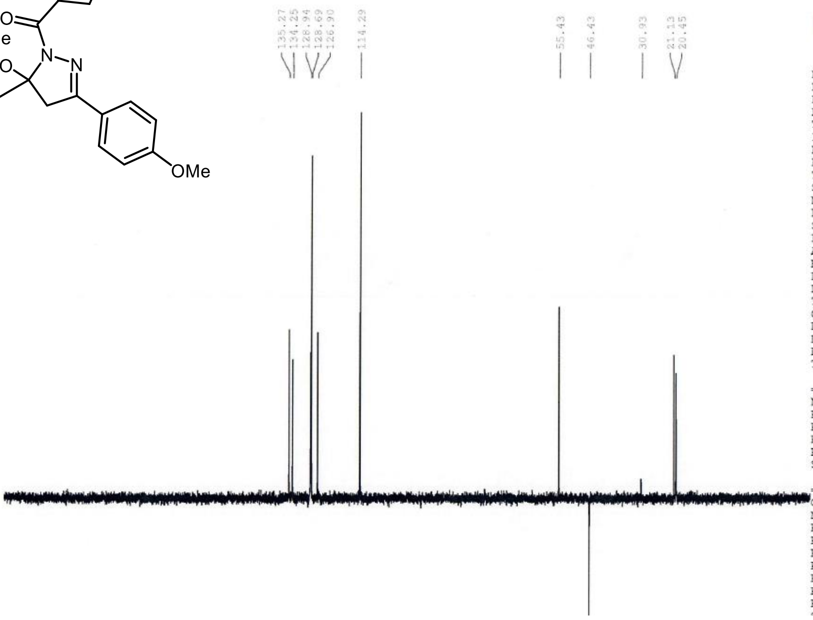

NAME Boersch
EXPNO 1382
PROCNO 1
Date_ 20121122
Time 14.27
INSTRUM spect
PROBHD 5 mm PABBO BB-
PULPROG dept135
TD 65536
SOLVENT CDCl3
NS 512
DS 4
SWH 18028.846 Hz
FIDRES 0.275098 Hz
AQ 1.8175818 sec
RG 2050
DW 27.733 usec
DE 6.50 usec
TE 298.0 K
CNST2 145.0000000
D1 2.00000000 sec
D2 0.00344828 sec
D12 0.00002000 sec
TD0 2

======== CHANNEL f1 ========
NUC1 13C
P1 7.70 usec
P2 15.40 usec
PL1 -3.00 dB
PL1W 47.86338043 W
SFO1 75.4752953 MHz

======== CHANNEL f2 ========
CPDPRG2 waltz16
NUC2 1H
P3 9.00 usec
P4 18.00 usec
PCPD2 80.00 usec
PL2 -2.00 dB
PL12 17.00 dB
PL2W 17.95463371 W
PL12W 0.22603545 W
[illegible]

6 Crystal structures of compounds 5a, 5r, and 6a

All studied crystals were selected directly from a representative sample of the compound. For the data collection the crystal was glued on a thin glass thread. Diffraction data collection for **5a** was performed using the Oxford Xcalibur four-circle diffractometer;[2] for **5r**, an STOE IPDS was used and for **6a** the STOE IPDS2 was used. Data collection followed in all cases the standard procedures. The same is true for the refinement using the SHELX program system.[3] The DIAMOND[4] software was used for the figures showing the crystal structures presented in this paper.

CCDC 1902138 (**5a**), CCDC 1906570 (**5r**), and CCDC 1906571 (**6a**) contain the supplementary crystallographic data for this paper. The data can be obtained free of charge from The Cambridge Crystallographic Data Centre via www.ccdc.cam.ac.uk/structures.

[2] Oxford diffraction. CryAlis^{PRO} Version 1.171.34.44, Oxford, UK, **2010**.
[3] Sheldrick. G. M. *Acta Crystallogr.* **2015**, *C71*, 3-8. doi: 10.1107/S2053229614024218
[4] Brandenburg, K.: *DIAMOND*. Visual Crystal Structure Information System. Version 4.5.2. Crystal Impact, Bonn, Germany, 2018.

6.1 Crystal structure of *tert*-butyl 5-benzoyl-5-hydroxy-3-phenyl-4,5-dihydro-1*H*-pyrazole-1-carboxylate (5a)

Crystal data and the results of the structure refinement for 5a

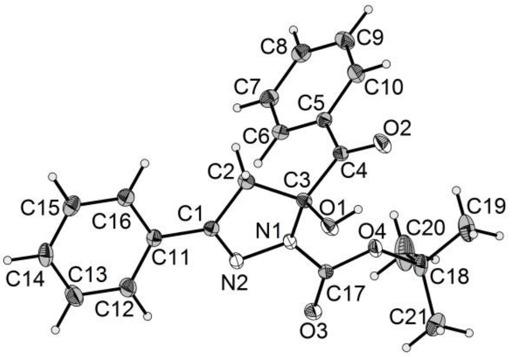

**Identification code**	**CCDC 1902138**
Empirical formula:	$C_{21}H_{22}N_2O_4$
Formula weight:	366.40 g/mol
Temperature:	129 K
Wavelength:	0.71073 Å
Crystal system:	monoclinic
Space group	$P2_1/n$
Unit cell dimensions:	a = 9.9453(3) Å
	b = 18.3337(4) Å
	c = 11.4431(4) Å
	β = 113.377(4)°
Volume:	1915.22(10) Å^3
Z:	4
Density (calculated):	1.271 mg/m^3
Absorption coefficient μ:	0.09 mm^{-1}
Structural factor F(000):	776
Crystal size:	0.5 x 0.4 x 0.3 mm^3
Theta range of data collection:	3.0 to 29.0° *
Index ranges:	-12 ≤ h ≤ 13, -22 ≤ k ≤ 23, -14 ≤ l ≤ 6
Reflections collected:	8382
Independent reflections:	4366 [R_{int} = 0.021]
Goodness-of-fit of F^2:	1.05
Final R indices [$I > 2\sigma(I)$]	$R1$ = 0.0409, $wR2$ = 0.0735
Final R indices [all]:	$R1$ = 0.0565, $wR2$ = 0.0798
Largest diff. Peak and hole:	0.34 und -0.23 eÅ^{-3}

*Completeness >99% up to theta = 25°.

6.2 Crystal structure of (5-hydroxy-3-(4-methoxyphenyl)-1-(thiophen-2-carbonyl)-4,5-dihydro-1*H*-pyrazol-5-yl) (mesityl)methanone (5r)

Crystal data and the results of the structure refinement for 5r

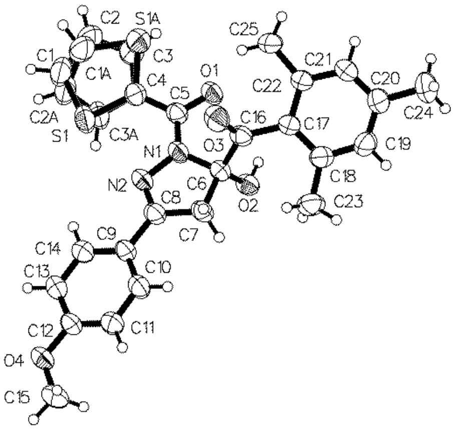

**Identification code:**	**CCDC 1906570**	
Empirical formula:	$C_{25}H_{24}N_2O_4S$	
Formula weight:	448.52 g/mol	
Temperature:	291(2) K	
Wavelength:	0.71073 Å	
Crystal system:	monoclinic	
Space group	$P2_1/c$	
Unit cell dimensions:	a = 11.0332(9) Å	α = 90°
	b = 9.9343(4) Å	β = 93.042(9)°
	c = 20.1694(15) Å	γ = 90°
Volume:	2207.6(3) $Å^3$	
Z:	4	
Density (calculated):	1.349 mg/m^3	
Absorption coefficient μ:	0.182 mm^{-1}	
Structural factor F(000):	944	
Crystal size:	0.4 x 0.4 x 0.4 mm^3	
Theta range of data collection:	2.67 to 25.00°	
Index ranges:	$-13 \le h \le 13$, $-11 \le k \le 11$, $-23 \le l \le 23$	
Reflections collected:	27965	
Independent reflections:	3878 [R_{int} = 0.0661]	
Goodness-of-fit of F^2:	1.017	
Final R indices [I>2σ(I)]	R1 = 0.0392, wR2 = 0.1099	
Final R indices [all]:	R1 = 0.0496, wR2 = 0.1144	
Largest diff. Peak and hole:	0.215 und -0.164 e.$Å^{-3}$	

6.3 Crystal structure of phenyl(3-phenyl-1H-pyrazol-5-yl)methanone (6a)

Crystal data and the results of the structure refinement for 6a

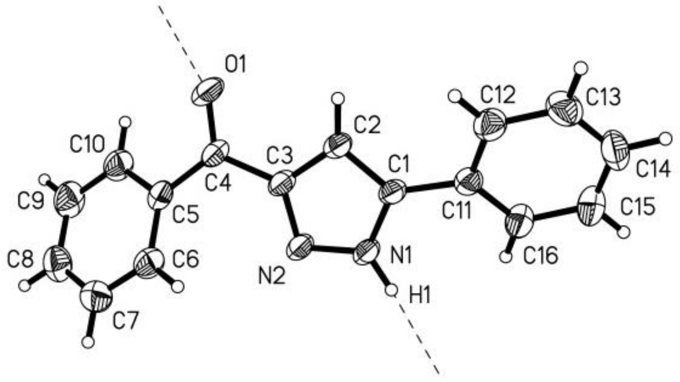

**Identification code**	**CCDC 1906571**	
Empirical formula	C_{16}H12N2O	
Formula weight	248.28	
Temperature	291(2) K	
Wavelength	0.71073 Å	
Crystal system	Monoclinic	
Space group	*Cc*	
Unit cell dimensions	a = 26.1458(17) Å	α = 90°
	b = 6.9379(3) Å	β = 100.656(5)°
	c = 7.1310(4) Å	γ = 90°
Volume	1271.24(12) $Å^3$	
Z	4	
Density (calculated)	1.297 Mg/m	
Absorption coefficient	0.083 mm^{-1}	
F(000)	520	
Crystal size	0.45 x 0.4 x 0.4 mm^3	
Theta range for data collection	3.04 to 24.99°	
Index ranges	-30 ≤ h ≤ 30, -8 ≤ k ≤ 8, -8 ≤ l ≤ 7	
Reflections collected	4609	
Independent reflections	1119 [R_{int} = 0.0635]	
Completeness to theta = 24.99°	99.6%	
Absorption correction	None	
Refinement method	Full-matrix least-squares on F^2	
Data / restraints / parameters	1942 / 2 / 173	
Goodness-of-fit on F^2	1.124	
Final R indices [$I > 2\sigma(I)$]	$R1$ = 0.0414, $wR2$ = 0.1051	
Final R indices [all]:	$R1$ = 0.0447, $wR2$ = 0.1077	
Absolute structure parameter	-4.6(10)	
Largest diff. peak and hole	0.149 and -0.162 e.$Å^{-3}$	
